# Supplementary material for: Tracking the emergence of the Upper Palaeolithic in western Asia and Europe: A Multiple Correspondence Analysis of Protoaurignacian and Southern Ahmarian lithics
Source: PLoS One. 2025 Sep 24;20(9):e0331393. doi: 10.1371/journal.pone.0331393 (PMC12459816; doi:10.1371/journal.pone.0331393)
Supplement: S1 File — (PDF) [file pone.0331393.s001.pdf]

Tracking the emergence of the Upper Palaeolithic in western Asia and Europe: a Multiple Correspondence Analysis of Protoaurignacian and southern Ahmarian lithics

Gennai Jacopo[1¶], Falcucci Armando[2¶], Niochet Vincent[3¶], Peresani Marco[4,5], Richter Jürgen[6], Soressi Marie[3]

[1] Department of Civilisations and Forms of Knowledge, University of Pisa, Pisa, Italy

[2] Department of Geosciences, Prehistory and Archaeological Sciences Research Unit, Eberhard Karls University of Tübingen, Tübingen, Germany

[3] Faculty of Archaeology, Leiden University, Leiden, The Netherlands

[4] Department of Humanities, University of Ferrara, Ferrara, Italy

[5] Institute of Environmental Geology and Geoengineering, National Research Council, Stratigraphic, Milano, Italy

[6] Institute for Prehistory, University of Cologne, Cologne, Germany

¶Corresponding authors

Email: jacopo.gennai@cfs.unipi.it, armando.falcucci@uni-tuebingen.de, v.niochet@arch.leidenuniv.nl, m.a.soressi@arch.leidenuniv.nl

Early Upper Palaeolithic what, where and when

Early Upper Palaeolithic technology overview

The definitions of the Early Upper Palaeolithic industries have come from a long research history spanning decades, if not a century (Bon, 2002; Breuil, 1907; de Sonneville-Bordes, 1955; Delporte et al., 1977; Garrod, 1957; Gilead, 1991; Goring-Morris and Davidzon, 2006; Laplace, 1966; Le Brun-Ricalens, 2005a; Neuviille, 1934). Here below a synthesis of the current understanding from a techno-typological view is provided. Furthermore, an overview of the geographical distribution of the facies is provided. Finally, a synthetic spreadsheet of EUP sites and relative references is provided alongside this text and it is the baseline for Fig 1 of the manuscript.

Ahmarian: Both the Northern and the Southern facies display similarities in the final core shape, as they are mostly narrow-fronted and the reduction progresses frontally (Abulafia et al., 2021; Bar-

Yosef and Belfer-Cohen, 2019; Gennai et al., 2023; Gilead and Bar-Yosef, 1993; Kuhn et al., 2009; Monigal, 2003; Ohnuma, 1988). No major preparations of the core are needed, the striking platform is plain and the abrasion of the overhang is the only customary procedure (Abulafia et al., 2021; Monigal, 2003). Decortication degree varies among sites, in general, both facies leave the core flank cortical. The main difference is the orientation of the knapping, as the Southern facies is strictly unidirectional, while the Northern facies has bidirectional and unidirectional cores. This mirrors in the obtained blanks as bladelets are much more common in the Southern facies and laminar blanks are slender (Goring-Morris and Belfer-Cohen, 2018; Kadowaki et al., 2015). Exclusively bladelet cores are rare, in the Southern facies they may resemble burin-cores, while in the Northern facies they are pyramidal, unidirectional ones (Abulafia et al., 2021; Hussain, 2015). Most retouched blanks are blades or bladelets with direct retouch: the el-Wad point (Abulafia et al., 2021; Bar-Yosef and Belfer-Cohen, 1977; Kuhn et al., 2009; Le Brun-Ricalens et al., 2009). Despite being the fossil guide of the Ahmarian there is very little standardisation about their manufacture as there is no general tendency in retouch position and extent (Gennai et al., 2023; Le Brun-Ricalens et al., 2009). Burins and endscrapers are often fashioned on by-products (Goring-Morris and Belfer-Cohen, 2018; Hussain, 2015; Monigal, 2003; Parow-Souchon et al., 2021).

Proto-Aurignacian: Cores are seldom prepared, instead they show plain striking platforms and a management of convexities embedded in the production (Bon, 2002; Falcucci et al., 2017). The direction of negatives and production is unidirectional (Bataille et al., 2018; Bon, 2002; Falcucci et al., 2017; Roussel and Soressi, 2013). Cores are either showing an intercalated blades-bladelets production (mostly pyramidal convergent core types) or exclusively bladelets production (pyramidal convergent and burin [on blank edge] cores) (Bataille et al., 2018; Bon, 2002; Falcucci et al., 2017; Roussel and Soressi, 2013). This leads to the production of numerous straight or slightly curved bladelets or microbladelets, defined as big bladelets (i.e. *grande lamelle*) between 20-40 mm long. They make up the majority of the retouched blanks as Dufour sub-type Dufour or bilateral obversely retouched bladelets (Bon, 2002; Bordes, 2005; Laplace, 1966). Blades are thin and slender because they are essentially produced in contemporaneity with bladelets. They can be retouched in simple endscrapers or dihedral burins. Therefore the big, scalarly retouched Aurignacian blades are rare and so the carinated endscrapers

Early Aurignacian: Blade cores undergo a swift preparation of lateral unifacial crests, the striking platform is usually facetted. Bladelet cores are generally unprepared, apart from laterally-struck flakes used to maintain the flaking surface convexities in carinated cores (*éclats de cintrage* (Bon, 2002) *éclats de ravivage* (Le Brun-Ricalens, 2005b)). The direction of negatives and production is unidirectional. Blade cores are prismatic with at least a straight parallel side bordering the flaking surface and whose intersection acts as the main way to maintain the lateral convexity of the core (*cintrage*). Bladelet cores can be diminished or small prismatic core, all alike blade cores, or more typically carinated cores. Carinated cores are produced on big flakes, large blades or chunks. The

long sides are shaped in a crest, achieving a triangular cross-section, which acts as the distal convexity (*carénage*). One of the short sides is transformed in the "front" of the endscraper, the flaking surface, maintenance of the lateral convexities is done through twisted bladelets and major reshaping through the above-mentioned flakes (Bon, 2002; Bordes, 2005; Chiotti and Cretin, 2011). The front is relatively large (25 mm (Pelegrin and O'Farrell, 2005)). Blades then are usually thick (10-16 mm (Bon, 2002)) and curved in profile. This results in a bigger presence of blade-retouched tools, in particular the Aurignacian blades, strangled blades and carinated scrapers on blade. Bladelets are then chiefly straight or slightly curved, their length is rarely exceeding 30 mm (i.e. *petite lamelle*). They are seldom retouched, but when they do they are classified as Dufour sub-type Dufour bladelets (Bon, 2002).

The geographical distribution of the EUP facies

Within the **Levant**, the presence of the Ahmarian is neatly split according to two geographical areas: the desertic Southern one and the Mediterranean one. The two facies of the Ahmarian are found exclusively in one of each and no co-occurrence in the same site is recorded. The Northern Ahmarian is typical of cave sites in the Mediterranean area, while the Southern Ahmarian is found in the desert open-air contexts of the Southern Levant (Goring-Morris and Belfer-Cohen, 2018; Richter et al., 2020). The only possible presence of both facies in the same stratigraphical sequence is Ksar Akil, where the Northern Ahmarian is found in layers XX – XVI and assemblages reminding of the Southern Ahmarian are found in layers XIII – IX (Bergman et al., 2017).

No EUP site is signalled in **Anatolia**, the nearest ones are those in the **Caucasian** highlands. These sites display a generally Upper Palaeolithic techno-typology, with mostly unidirectional knapping of slender blades and bladelets (Golovanova and Doronichev, 2012). The production of the two types of blank might be intercalated (Bar-Yosef et al., 2011; Pleurdeau et al., 2016) or distinct (Kandel et al., 2017). Even though some connections with the Ahmarian have been attempted or implied (Bar-Yosef et al., 2011; Golovanova and Doronichev, 2012), no explicit facies attribution is expressed.

In **Southeastern Europe** no Early Aurignacian is typically found, except for the split-based bone points of Istallos-kő and Pes-kő although associated lithics are not diagnostic (Markó, 2015), and only the Protoaurignacian facies (or the Kozarnikian) is commonly found (Bataille, 2016; Chu et al., 2022; Tsanova et al., 2012). Further East, at Kostenki both assemblages resembling Protoaurignacian and Early Aurignacian are found, however, links with the Western Aurignacian are discussed (Bataille et al., 2019; Dinnis et al., 2019; Sinitsyn, 2003).

In **Central Europe** no Protoaurignacian is found, except for the likely mixed assemblage of Krems-Hundssteig (Broglio and Laplace, 1966), instead the earliest Aurignacian facies is the Early Aurignacian that both in Geissenkloesterle and Willendorf (Higham et al., 2012; Nigst et al., 2014). In Central Europe, the analysis of Hohle Fels also shows another *facies*, the Swabian Aurignacian,

featuring a strong focus on small and slender bladelets, mostly obtained from carinated burins (Bataille and Conard, 2021, 2018).

The **Italian peninsula** features both Protoaurignacian and Early Aurignacian *facies*, with a higher occurrence of the former (Dini et al., 2012; Falcucci et al., 2024a, 2024b, 2017; Gambassini, 1997; Kuhn and Stiner, 1998; Palma di Cesnola, 2006). Grotta del Fossellone siliceous pebbles were mostly transformed in carinated cores and are associated with split-based and massive-based osseous points (Degano et al., 2019; Segre and Blanc, 1953). At Grotta di Castelcivita, the Early Aurignacian is sandwiched between the Protoaurignacian layer and the Campanian Ignimbrite tephra (Falcucci et al., 2024a). At Grotta della Cala, a rich Early Aurignacian assemblage was recently described. This features two previously unrecognized split-based antler points and many carinated cores for producing miniaturised bladelets (Falcucci et al., in preparation). Despite previous work at Grotta di Fumane not finding substantial differences between the earlier Protoaurignacian and the later Aurignacian (Falcucci et al., 2020), a recent lithic taphonomic and technological reassessment has allowed Falcucci and colleagues to identify an Early Aurignacian assemblage (layer D3b alpha) on top of the Protoaurignacian from layers A2–A1 (Falcucci et al., 2024b).

In the **Liguro-Provençal Basin**, the Early Aurignacian is reported at sites such as Riparo Mochi and Grotte de l'Observatoire above the Protoaurignacian mostly on the grounds of osseous technology (Porraz et al., 2010; Riel-Salvatore and Negrino, 2018; Tejero and Grimaldi, 2015). Despite limited information, an Early Aurignacian assemblage is reported at Esquicho Grapaou in Southeastern France (Barshay-Szmidt et al., 2020; Bazile, 2005). This region, however, does not consistently feature Early Aurignacian assemblages. Most of the sites are attributed to the Protoaurignacian (Barshay-Szmidt et al., 2020; Porraz et al., 2010; Slimak et al., 2002).

Southwestern and central France are the richest areas for EUP sites, here the Protoaurignacian occurs before than the Early Aurignacian and the two facies are well distinguished from the techno-typological view (Bon, 2002; Bordes, 2005; Roussel and Soressi, 2013).

Northern Iberia and the Pyrenees area follow Southwestern France's signature, even though the new technological data suggest a much more nuanced technological picture (Barshay-Szmidt et al., 2018; Cabrera Valdés et al., 2002; Deschamps and Flas, 2019; Maíllo Fernández, 2005; Ortega Cobos et al., 2005; Santamaría Álvarez, 2013). A renewed analysis of Northern Iberia's Aurignacian assemblages failed to find meaningful differences between assemblages attributed to one of the two *facies* (Bataille et al., 2018; Tafelmaier, 2017).

Early Upper Palaeolithic dating

Radiometric dating is extremely valuable for constructing a chronological framework and tracing technological and dispersal developments. The most commonly used radiometric dating methods in the Early Upper Palaeolithic (EUP) timeframe are radiocarbon dating, which measures the ratio of

$^{14}C/^{12}C$ in organic samples, and luminescence dating methods, such as Thermoluminescence (TL) and Optically Stimulated Luminescence (OSL), which determine the last exposure of inorganic samples to light or intense heat (Durcan, 2021; Hajdas et al., 2021). Radiometric dating methods have inherent limitations, particularly when applied to the relatively short-lived EUP technocomplexes. The radiocarbon dating limit is currently 55,000 years due to the half-life of ^{14}C (Hajdas et al., 2021). Additionally, contamination of organic samples can lead to discrepancies in radiocarbon dating results (Higham, 2011; Higham et al., 2006). More aggressive pretreatment protocols, such as ABOx-Sc (Acid-Base-Oxidation-Stepped combustion) and ultrafiltration, have been shown, at least in European contexts, to produce older dates than traditional protocols like ABA (Acid-Base-Acid) or AAA (Acid-Alkali-Acid) (Higham, 2011; Higham et al., 2009, 2006). However, a comparison of the ABA and ABOx-Sc methods at Kebara revealed that the former, rather than the latter, yielded older dates (Rebollo et al., 2011). In bone dating, collagen yield is crucial for achieving reliable determinations, and older samples or those from contexts with poor organic preservation are likely to yield less collagen (Barshay-Szmidt et al., 2018; Brock et al., 2012). Radiocarbon dates always include an error margin, which is typically ±20–30 years for samples younger than 10,000 years (Hajdas et al., 2021). For older samples, such as those from the EUP timeframe, the error margin can reach hundreds or even thousands of years. Furthermore, all radiocarbon dates from the EUP require calibration against high-resolution, continuously deposited reference data to yield a calendrical date. Efforts to refine and improve the calibration curve with new reference datasets are ongoing (Hajdas et al., 2021; Reimer, 2022). Calibrated dates provide a probability range, typically at 95% or 68% confidence intervals (Hajdas et al., 2021). A 95% probability range offers greater confidence but results in a broader interval, increasing the likelihood of overlap with other dates. Beyond calibration, Bayesian statistical methods have been introduced in the last two decades. These methods apply prior conditions, such as stratigraphic sequences, to produce refined chronological estimates(Bronk Ramsey, 2009). The resulting modelled dates offer an enhanced chronological framework for archaeological sites, allowing for the correction of problematic dates and the estimation of undated contexts that fall between dated ones. While useful at the intra-site level, Bayesian modelling is not applicable at the inter-site level, as prior assumptions cannot be reliably made for different sites. TL and OSL dating extend much further than radiocarbon dating, up to 200,000 years. However, the typical error margin for OSL is often 10% of the measurement, while for TL it is 15–20% (Durcan, 2021; Nelson et al., 2015). This results in larger errors than radiocarbon dating, making these methods less suitable for resolving finer chronological timeframes(Higham et al., 2024; Moroni et al., 2019). Nevertheless, in some cases, luminescence dating has provided more reliable estimates than radiocarbon (Jacobs et al., 2015). Also, luminescence dating does not need calibration as it provides already a calendrical date and it does not rely on organic preservation. Nevertheless, proving the association between the dated sample (often soil) and the archaeological artefacts requires the absence of major postdepositional processes.

Unlike radiocarbon dating, luminescence dating does not require calibration, as it directly produces a calendrical date and is not dependent on organic preservation. However, demonstrating the association between the dated sample (often soil) and archaeological artefacts requires ensuring that major post-depositional processes have not disrupted the context.

Considering these factors, caution must be exercised when comparing dates at intra-site, inter-site, regional, or continental scales. Differences in preservation conditions, dating methods, sampling and pretreatment protocols, calibration curves, and statistical modelling can all impact the results.

The compiled radiocarbon dates for contexts belonging to the Early Upper Palaeolithic (EUP) reveal overlapping timespans for the four technocomplexes (see SI File 2 – EUP_Dates and SI Fig. 1). Despite this overlap, distinct patterns emerge. The Northern Ahmarian shows two primary peaks, one around 45–43 ka cal BP and another around 42–40.5 ka cal BP. The Southern Ahmarian begins around 42 ka cal BP, with the main distribution of dates concentrated between 40–37.5 ka cal BP. The Protoaurignacian has a main distribution of dates between 42–39 ka cal BP, while the Early Aurignacian shows dates concentrated between 41–39 ka cal BP.

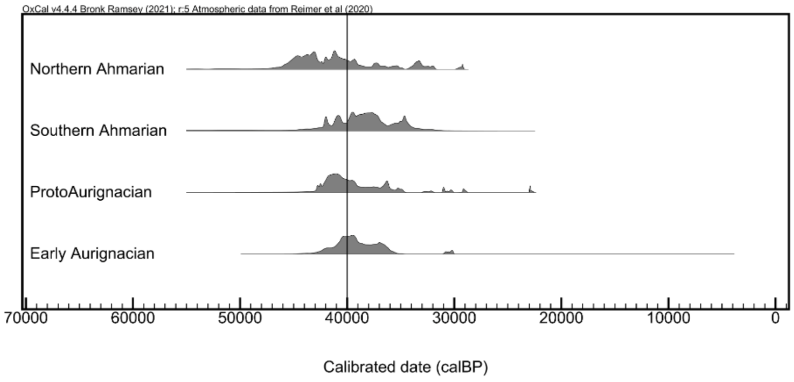

SI Fig 1. ***Compilation of available radiocarbon dates of Northern Ahmarian, Southern Ahmarian, Protoaurignacian and Early Aurignacian contexts****. The intervals are obtained with the command Sum() in OxCal 4.4.4.*

References

Abulafia, T., Goder-Goldberger, M., Berna, F., Barzilai, O., Marder, O., 2021. A technotypological analysis of the Ahmarian and Levantine Aurignacian assemblages from Manot Cave (area C) and the interrelation with site formation processes. Journal of Human Evolution 160, 102707. https://doi.org/10.1016/j.jhevol.2019.102707

Alex, B., Barzilai, O., Hershkovitz, I., Marder, O., Berna, F., Caracuta, V., Abulafia, T., Davis, L., Goder-Goldberger, M., Lavi, R., Mintz, E., Regev, L., Bar-Yosef Mayer, D., Tejero, J.-M., Yeshurun, R., Ayalon, A., Bar-Matthews, M., Yasur, G., Frumkin, A., Latimer, B., Hans, M.G., Boaretto, E., 2017. Radiocarbon chronology of Manot Cave, Israel and Upper Paleolithic dispersals. Sci. Adv. 3, e1701450. https://doi.org/10.1126/sciadv.1701450

Barshay-Szmidt, C., Bazile, F., Brugal, J.-P., 2020. First AMS 14C dates on the Protoaurignacian in Mediterranean France: The site of Esquicho-Grapaou (Russan-Ste-Anastasie, Gard). Journal of Archaeological Science: Reports 33, 102474. https://doi.org/10.1016/j.jasrep.2020.102474

Barshay-Szmidt, C., Normand, C., Flas, D., Soulier, M.-C., 2018. Radiocarbon dating the Aurignacian sequence at Isturitz (France): Implications for the timing and development of the

Protoaurignacian and Early Aurignacian in western Europe. Journal of Archaeological Science: Reports 17, 809–838. https://doi.org/10.1016/j.jasrep.2017.09.003
Bar-Yosef, O., Belfer-Cohen, A., 2019. The Upper Paleolithic Industries of Kebara Cave, in: Meignen, L., Bar-Yosef, O. (Eds.), Kebara Cave, Mt. Carmel, Israel: The Middle and Upper Paleolithic Archaeology: Part 2, American School of Prehistoric Research. Peabody Museum of Archaeology and Ethnology, Harvard University, pp. 309–401.
Bar-Yosef, O., Belfer-Cohen, A., 1977. The Lagaman Industry, in: Bar-Yosef, O., Phillips, J.L. (Eds.), PREHISTORIC INVESTIGATIONS IN GEBEL MAGHARA, NORTHERN SINAI. pp. 42–85.
Bar-Yosef, O., Belfer-Cohen, A., Mesheviliani, T., Jakeli, N., Bar-Oz, G., Boaretto, E., Goldberg, P., Kvavadze, E., Matskevich, Z., 2011. Dzudzuana: an Upper Palaeolithic cave site in the Caucasus foothills (Georgia). Antiquity 85, 331–349. https://doi.org/10.1017/S0003598X0006779X
Bataille, G., 2016. Extracting the “Proto” from the Aurignacian. Distinct Production Sequences of Blades and Bladelets in the Lower Aurignacian Phase of Siuren I, Units H and G (Crimea). Mitteilungen der Gesellschaft für Urgeschichte 25, 49–83.
Bataille, G., Conard, N.J., 2021. Burin-core technology in Aurignacian horizons IIIa and IV of Hohle Fels Cave (Southwestern Germany). Quartär – Internationales Jahrbuch zur Erforschung des Eiszeitalters und der Steinzeit 7-49 Seiten. https://doi.org/10.7485/QU65_1
Bataille, G., Conard, N.J., 2018. Blade and bladelet production at Hohle Fels Cave, AH IV in the Swabian Jura and its importance for characterizing the technological variability of the Aurignacian in Central Europe. PLoS ONE 13, e0194097. https://doi.org/10.1371/journal.pone.0194097
Bataille, G., Falcucci, A., Tafelmaier, Y., Conard, N.J., 2019. Technological differences between Kostenki 17/II (Spitsynskaya industry, Central Russia) and the Protoaurignacian: Reply to Dinnis et al. (2019). Journal of Human Evolution 102685. https://doi.org/10.1016/j.jhevol.2019.102685
Bataille, G., Tafelmaier, Y., Weniger, G.-C., 2018. Living on the edge – A comparative approach for studying the beginning of the Aurignacian. Quaternary International 474, 3–29. https://doi.org/10.1016/j.quaint.2018.03.024
Bazile, F., 2005. La composante lamellaire dans l’Aurignacien Initial de la France Méditerranéenne, in: Le Brun-Ricalens, F. (Ed.), Productions lamellaires attribuées à l’Aurignacien: actes du XIVe congrès de l’UISPP, Université de Liège, 2-8 septembre 2001, section 6, symposium C6.7, ArchéoLogiques. Musée National d’Histoire et d’Art, Luxèmbourg, pp. 325–336.
Bergman, C.A., Williams, J., Douka, K., Schyle, D., 2017. The Palaeolithic Sequence of Ksar ‘Akil, Lebanon, in: Enzel, Y., Bar-Yosef, O. (Eds.), Quaternary of the Levant. Cambridge University Press, pp. 267–276. https://doi.org/10.1017/9781316106754.030
Boaretto, E., Hernandez, M., Goder-Goldberger, M., Aldeias, V., Regev, L., Caracuta, V., McPherron, S.P., Hublin, J.-J., Weiner, S., Barzilai, O., 2021. The absolute chronology of Boker Tachtit (Israel) and implications for the Middle to Upper Paleolithic transition in the Levant. Proc Natl Acad Sci USA 118, e2014657118. https://doi.org/10.1073/pnas.2014657118
Bon, F., 2002. L’ Aurignacien entre mer et océan: réflexion sur l’unité des phases anciennes de l’Aurignacien dans le sud le la France, Mémoire / Société Préhistorique Française. Soc. Préhistorique Française, Paris.
Bordes, J.-G., 2005. La séquence aurignacienne du nord de l’Aquitaine: variabilité des productions lamellaires à Caminade-Est, Roc-de-Combe, Le Piage et Corbiac-Vignoble II, in: Le Brun-Ricalens, F. (Ed.), Productions lamellaires attribuées à l’Aurignacien: actes du XIVe congrès de l’UISPP, Université de Liège, 2-8 septembre 2001, section 6, symposium C6.7, Archéologiques. Musée National d’Histoire et d’Art, Luxembourg, pp. 123–154.
Bosch, M.D., Mannino, M.A., Prendergast, A.L., O’Connell, T.C., Demarchi, B., Taylor, S.M., Niven, L., van der Plicht, J., Hublin, J.-J., 2015. New chronology for Ksâr ‘Akil (Lebanon) supports Levantine route of modern human dispersal into Europe. Proc Natl Acad Sci USA 112, 7683–7688. https://doi.org/10.1073/pnas.1501529112
Breuil, H., 1907. La question aurignacienne. Étude critique de stratigraphie comparee. Revue préhistorique 6 & 7, 1–47.
Brock, F., Wood, R., Higham, T.F.G., Ditchfield, P., Bayliss, A., Ramsey, C.B., 2012. Reliability of Nitrogen Content (%N) and Carbon:Nitrogen Atomic Ratios (C:N) as Indicators of Collagen

Preservation Suitable for Radiocarbon Dating. Radiocarbon 54, 879–886. https://doi.org/10.1017/S0033822200047524
Broglio, A., Laplace, G., 1966. Etudes de typologie analytique des complexes leptolithiques de l'Europe centrale. I. Les complexes aurignacoïdes de la Basse Autriche. Rivista di Scienze Preistoriche XXI, 61–121.
Bronk Ramsey, C., 2009. Bayesian Analysis of Radiocarbon Dates. Radiocarbon 51, 337–360. https://doi.org/10.1017/S0033822200033865
Cabrera Valdés, V., de Quirós, F.B., Maíllo Fernández, J.M., Valladas, H., Martinez de La Riva Llorret, M., 2002. El Auriñaciense arcaico de El Castillo (Cantabria). Espacio Tiempo y Forma, I 15, 67–86.
Chiotti, L., Cretin, C., 2011. Les mises en forme de grattoirs carénés / nucléus de l'aurignacien ancien de l'abri Castanet (Sergeac, Dordogne). paleo 69–84. https://doi.org/10.4000/paleo.2077
Chu, W., McLin, S., Wöstehoff, L., Ciornei, A., Gennai, J., Marreiros, J., Doboș, A., 2022. Aurignacian dynamics in Southeastern Europe based on spatial analysis, sediment geochemistry, raw materials, lithic analysis, and use-wear from Românești-Dumbrăvița. Sci Rep 12, 14152. https://doi.org/10.1038/s41598-022-15544-5
de Sonneville-Bordes, D., 1955. La question du Périgordien II. bspf 52, 187–203. https://doi.org/10.3406/bspf.1955.3176
Degano, I., Soriano, S., Villa, P., Pollarolo, L., Lucejko, J.J., Jacobs, Z., Douka, K., Vitagliano, S., Tozzi, C., 2019. Hafting of Middle Paleolithic tools in Latium (central Italy): New data from Fossellone and Sant'Agostino caves. PLoS ONE 14, e0213473. https://doi.org/10.1371/journal.pone.0213473
Delporte, H., Mazière, G., Djindjian, F., 1977. L'Aurignacien de La Ferrassie. Observations préliminaires à la suite de fouilles récentes. bspf 74, 343–361. https://doi.org/10.3406/bspf.1977.8457
Deschamps, M., Flas, D., 2019. Paléolithique moyen récent et Paléolithique supérieur initial en contexte pyrénéen : les industries lithiques de la grotte de Gatzarria (Ossas-Suhare, Pyrénées-Atlantiques) et leurs implications régionales, in: Deschamps, M., Costamagno, S., Milcent, P.-Y., Pétillon, J.-M., Renard, C., Valdeyron, N. (Eds.), La conquête de la montagne : des premières occupations humaines à l'anthropisation du milieu. Éditions du Comité des travaux historiques et scientifiques, pp. 1–25. https://doi.org/10.4000/books.cths.6302
Dini, M., Baills, H., Conforti, J., Tozzi, C., 2012. Le Protoaurignacien de la Grotte La Fabbrica (Grosseto, Italie) dans le contexte de l'arc nord méditerranéen. L'Anthropologie 116, 550–574. https://doi.org/10.1016/j.anthro.2012.10.003
Dinnis, R., Bessudnov, A., Reynolds, N., Devièse, T., Pate, A., Sablin, M., Sinitsyn, A., Higham, T., 2019. New data for the Early Upper Paleolithic of Kostenki (Russia). Journal of Human Evolution 127, 21–40. https://doi.org/10.1016/j.jhevol.2018.11.012
Douka, K., 2013. Exploring "the great wilderness of prehistory": The Chronology of the Middle to the Upper Paleolithic Transition in the Northern Levant. Mitteilungen der Gesellschaft für Urgeschichte 22, 11–40.
Douka, K., Bergman, C.A., Hedges, R.E.M., Wesselingh, F.P., Higham, T.F.G., 2013. Chronology of Ksar Akil (Lebanon) and Implications for the Colonization of Europe by Anatomically Modern Humans. PLOS ONE 8, e72931. https://doi.org/10.1371/journal.pone.0072931
Durcan, J.A., 2021. Luminescence Dating, in: Encyclopedia of Geology. Elsevier, pp. 164–174. https://doi.org/10.1016/B978-0-12-409548-9.12105-0
Falcucci, A., Arrighi, S., Spagnolo, V., Rossini, M., Higgins, O.A., Muttillo, B., Martini, I., Crezzini, J., Boschin, F., Ronchitelli, A., Moroni, A., 2024a. A pre-Campanian Ignimbrite techno-cultural shift in the Aurignacian sequence of Grotta di Castelcivita, southern Italy. Sci Rep 14, 12783. https://doi.org/10.1038/s41598-024-59896-6
Falcucci, A., Conard, N.J., Peresani, M., 2020. Breaking through the Aquitaine frame: A re-evaluation on the significance of regional variants during the Aurignacian as seen from a key record in southern Europe. Journal of Anthropological Sciences 98, 42.
Falcucci, A., Conard, N.J., Peresani, M., 2017. A critical assessment of the Protoaurignacian lithic technology at Fumane Cave and its implications for the definition of the earliest Aurignacian. PLoS ONE 12, e0189241. https://doi.org/10.1371/journal.pone.0189241

Falcucci, A., Giusti, D., Zangrossi, F., de Lorenzi, M., Ceregatti, L., Peresani, M., 2024b. Refitting the context: A reconsideration of cultural change among early Homo sapiens at Fumane Cave through blade break connections, spatial taphonomy, and lithic technology. J Paleo Arch 8. https://doi.org/10.1007/s41982-024-00203-0

Gambassini, P., 1997. Le industrie paleolitiche di Castelcivita, in: Il Paleolitico di Castelcivita : culture e ambiente. Electa Napoli, Napoli, pp. 92–145.

Garrod, D., 1957. Notes sur le Paléolithique Supérieur du Moyen Orient. bspf 54, 439–446. https://doi.org/10.3406/bspf.1957.7854

Gennai, J., Schemmel, M., Richter, J., 2023. Pointing to the Ahmarian. Lithic Technology and the El-Wad Points of Al-Ansab 1. J Paleo Arch 6, 6. https://doi.org/10.1007/s41982-022-00131-x

Gilead, I., 1991. The Upper Paleolithic period in the Levant. J World Prehist 5, 105–154. https://doi.org/10.1007/BF00974677

Gilead, I., Bar-Yosef, O., 1993. Early Upper Paleolithic Sites in the Qadesh Barnea Area, NE Sinai. Journal of Field Archaeology 20, 265–280.

Golovanova, L.V., Doronichev, V.B., 2012. The Early Upper Paleolithic of the Caucasus in the West Eurasian Context, in: Otte, M., Shidrang, S., Flas, D. (Eds.), L'Aurignacien de La Grotte Yafteh et Son Contexte (Fouilles 2005-2008) /The Aurignacian of Yafteh Cave and Its Context (2005-2008 Excavations). Universite de Liege, Liege, pp. 137–160.

Goring-Morris, N., Belfer-Cohen, A., 2018. The Ahmarian in the Context of the Earlier Upper Palaeolithic in the Near East, in: Nishiaki, Y., Akazawa, T. (Eds.), The Middle and Upper Paleolithic Archeology of the Levant and Beyond. Springer Singapore, Singapore, pp. 87–104. https://doi.org/10.1007/978-981-10-6826-3_7

Goring-Morris, N., Davidzon, A., 2006. Straight to the point: Upper Paleolithic Ahmarian lithic technology in the Levant. Anthropologie XLIV, 93–111.

Hajdas, I., Ascough, P., Garnett, M.H., Fallon, S.J., Pearson, C.L., Quarta, G., Spalding, K.L., Yamaguchi, H., Yoneda, M., 2021. Radiocarbon dating. Nat Rev Methods Primers 1, 62. https://doi.org/10.1038/s43586-021-00058-7

Higham, T., 2011. European Middle and Upper Palaeolithic radiocarbon dates are often older than they look: problems with previous dates and some remedies. Antiquity 85, 235–249. https://doi.org/10.1017/S0003598X00067570

Higham, T., Basell, L., Jacobi, R., Wood, R., Ramsey, C.B., Conard, N.J., 2012. Testing models for the beginnings of the Aurignacian and the advent of figurative art and music: The radiocarbon chronology of Geißenklösterle. Journal of Human Evolution 62, 664–676. https://doi.org/10.1016/j.jhevol.2012.03.003

Higham, T., Brock, F., Peresani, M., Broglio, A., Wood, R., Douka, K., 2009. Problems with radiocarbon dating the Middle to Upper Palaeolithic transition in Italy. Quaternary Science Reviews 28, 1257–1267. https://doi.org/10.1016/j.quascirev.2008.12.018

Higham, T., Frouin, M., Douka, K., Ronchitelli, A., Boscato, P., Benazzi, S., Crezzini, J., Spagnolo, V., McCarty, M., Marciani, G., Falcucci, A., Rossini, M., Arrighi, S., Dominici, C., Devièse, T., Schwenninger, J.-L., Martini, I., Moroni, A., Boschin, F., 2024. Chronometric data and stratigraphic evidence support discontinuity between Neanderthals and early Homo sapiens in the Italian Peninsula. Nat Commun 15, 8016. https://doi.org/10.1038/s41467-024-51546-9

Higham, T.F.G., Jacobi, R.M., Ramsey, C.B., 2006. AMS Radiocarbon Dating of Ancient Bone Using Ultrafiltration. Radiocarbon 48, 179–195. https://doi.org/10.1017/S0033822200066388

Hussain, S.T., 2015. Betwixt seriality and sortiment: rethinking early Ahmarian blade technology in Al-Ansab 1, in: Schyle, D., Richter, J. (Eds.), Pleistocene Archaeology of the Petra Area in Jordan, Band 5. Leidorf, Rahden/Westf, pp. 131–147.

Jacobs, Z., Li, B., Jankowski, N., Soressi, M., 2015. Testing of a single grain OSL chronology across the Middle to Upper Palaeolithic transition at Les Cottés (France). Journal of Archaeological Science 54, 110–122. https://doi.org/10.1016/j.jas.2014.11.020

Kadowaki, S., Omori, T., Nishiaki, Y., 2015. Variability in Early Ahmarian lithic technology and its implications for the model of a Levantine origin of the Protoaurignacian. Journal of Human Evolution 82, 67–87. https://doi.org/10.1016/j.jhevol.2015.02.017

Kandel, A.W., Gasparyan, B., Allué, E., Bigga, G., Bruch, A.A., Cullen, V.L., Frahm, E., Ghukasyan, R., Gruwier, B., Jabbour, F., Miller, C.E., Taller, A., Vardazaryan, V., Vasilyan, D., Weissbrod,

L., 2017. The earliest evidence for Upper Paleolithic occupation in the Armenian Highlands at Aghitu-3 Cave. Journal of Human Evolution 110, 37–68. https://doi.org/10.1016/j.jhevol.2017.05.010

Kuhn, S.L., Stiner, M.C., 1998. The Earliest Aurignacian of Riparo Mochi (Liguria, Italy). Current Anthropology 39, S175–S189. https://doi.org/10.1086/204694

Kuhn, S.L., Stiner, M.C., Güleç, E., Özer, I., Yılmaz, H., Baykara, I., Açıkkol, A., Goldberg, P., Molina, K.M., Ünay, E., Suata-Alpaslan, F., 2009. The early Upper Paleolithic occupations at Üçağızlı Cave (Hatay, Turkey). Journal of Human Evolution 56, 87–113. https://doi.org/10.1016/j.jhevol.2008.07.014

Laplace, G., 1966. Recherches sur l'origine et l'évolution des complexes leptolithiques. École Française de Rome, Rome.

Le Brun-Ricalens, F., 2005a. Productions lamellaires attribuées à l'Aurignacien: actes du XIVe congrès de l'UISPP, Université de Liège, 2-8 septembre 2001, section 6, symposium C6.7, Archéologiques. Musée national d'histoire et d'art, Luxembourg.

Le Brun-Ricalens, F., 2005b. Reconnaissance d' un "conept technoculture" de l'Aurignacien Ancien?, in: Le Brun-Ricalens, F. (Ed.), Productions lamellaires attribuées à l'Aurignacien: actes du XIVe congrès de l'UISPP, Université de Liège, 2-8 septembre 2001, section 6, symposium C6.7, Archéologiques. Musée National d'Histoire et d'Art, Luxembourg, pp. 157–190.

Le Brun-Ricalens, F., Bordes, J.-G., Eizenberg, L., 2009. A crossed-glance between southern European and Middle-Near Eastern early Upper Palaeolithic lithic technocomplexes. Existing models, new perspectives, in: Camps, M., Szmidt, C.C. (Eds.), The Mediterranean from 50 000 to 25 000 BP: Turning Points and New Directions. Oxford, pp. 11–33.

Maíllo Fernández, J.M., 2005. La production lamellaire de l'Aurignacien de la grotte Morin (Cantabrie, Espagne), in: Le Brun-Ricalens, F. (Ed.), Productions lamellaires attribuées à l'Aurignacien: actes du XIVe congrès de l'UISPP, Université de Liège, 2-8 septembre 2001, section 6, symposium C6.7, Archéologiques. Musée National d'Histoire et d'Art, Luxembourg, pp. 339–357.

Markó, A., 2015. Istállóskő revisited: Lithic artefacts and assemblages, sixty years after. Acta Archaeologica Academiae Scientiarum Hungaricae 66, 5–38. https://doi.org/10.1556/072.2015.66.1.1

Monigal, K., 2003. Technology, Economy, and Mobility at the Beginning of the Levantine Upper Palaeolithic, in: Goring-Morris, N., Belfer-Cohen, A. (Eds.), More than Meets the Eye: Studies on Upper Palaeolithic Diversity in the Near East. Oxbow Books ; David Brown Book Co., Oxford : Oakville, CT, pp. 118–133. https://doi.org/10.2307/j.ctvh1dwcq

Moroni, A., Boschian, G., Crezzini, J., Montanari-Canini, G., Marciani, G., Capecchi, G., Arrighi, S., Aureli, D., Berto, C., Freguglia, M., Araujo, A., Scaramucci, S., Hublin, J.J., Lauer, T., Benazzi, S., Parenti, F., Bonato, M., Ricci, S., Talamo, S., Segre, A.G., Boschin, F., Spagnolo, V., 2019. Late Neandertals in central Italy. High-resolution chronicles from Grotta dei Santi (Monte Argentario - Tuscany). Quaternary Science Reviews 217, 130–151. https://doi.org/10.1016/j.quascirev.2018.11.021

Nelson, M.S., Gray, H.J., Johnson, J.A., Rittenour, T.M., Feathers, J.K., Mahan, S.A., 2015. User Guide for Luminescence Sampling in Archaeological and Geological Contexts. Adv. archaeol. pract. 3, 166–177. https://doi.org/10.7183/2326-3768.3.2.166

Neuviille, R., 1934. LE PRÉHISTORIQUE DE PALESTINE. Revue Biblique 43, 237–259.

Nigst, P.R., Haesaerts, P., Damblon, F., Frank-Fellner, C., Mallol, C., Viola, B., Götzinger, M., Niven, L., Trnka, G., Hublin, J.-J., 2014. Early modern human settlement of Europe north of the Alps occurred 43,500 years ago in a cold steppe-type environment. Proc Natl Acad Sci USA 111, 14394–14399. https://doi.org/10.1073/pnas.1412201111

Ohnuma, K., 1988. Ksar Akil, Lebanon. A Technological Analysis of the Earlier Palaeolithic Levels of Ksar Akil. Volume III: Levels XXV-XIV. BAR Publishing, Oxford, England.

Ortega Cobos, D., Soler Masferrer, N., Maroto Genover, J., 2005. La production des lamelles pendant l'Aurignacien Archaique dans la grotte de l'Arbreda: organisation de la production, variabilité des méthodes et des objectifs, in: Le Brun-Ricalens, F. (Ed.), Productions lamellaires attribuées à l'Aurignacien: actes du XIVe congrès de l'UISPP, Université de Liège, 2-8 septembre 2001, section 6, symposium C6.7, Archéologiques. Musée National d'Histoire et d'Art, Luxembourg, pp. 359–373.

Palma di Cesnola, A., 2006. L'Aurignacien et le Gravettien ancien de la grotte Paglicci au Mont Gargano. L'Anthropologie 110, 355–370. https://doi.org/10.1016/j.anthro.2006.06.011

Parow-Souchon, H., Hussain, S.T., Richter, J., 2021. Early Ahmarian Lithic Techno-Economy and Mobility at Al-Ansab 1, Wadi Sabra, Southern Jordan. Journal of The Israel Prehistoric Society 6–64.

Pelegrin, J., O'Farrell, M., 2005. Les lamelles retouchées ou utilisées de Castanet, in: Le Brun-Ricalens, F. (Ed.), Productions lamellaires attribuées à l'Aurignacien: actes du XIVe congrès de l'UISPP, Université de Liège, 2-8 septembre 2001, section 6, symposium C6.7, Archéologiques. Musée National d'Histoire et d'Art, Luxembourg, pp. 103–121.

Phillips, J.L., 1988. The Upper Paleolithic of the Wadi Feiran, Southern Sinai. paleo 14, 183–200. https://doi.org/10.3406/paleo.1988.4467

Phillips, J.L., Saca, I.N., 2002. Recent excavations at the site of Erq-el-Ahmar. Antiquity 76, 17–18. https://doi.org/10.1017/S0003598X0008964X

Pleurdeau, D., Moncel, M.-H., Pinhasi, R., Yeshurun, R., Higham, T., Agapishvili, T., Bokeria, M., Muskhelishvili, A., Le Bourdonnec, F.-X., Nomade, S., Poupeau, G., Bocherens, H., Frouin, M., Genty, D., Pierre, M., Pons-Branchu, E., Lordkipanidze, D., Tushabramishvili, N., 2016. Bondi Cave and the Middle-Upper Palaeolithic transition in western Georgia (south Caucasus). Quaternary Science Reviews 146, 77–98. https://doi.org/10.1016/j.quascirev.2016.06.003

Porraz, G., Simon, P., Pasquini, A., 2010. Identité technique et comportements économiques des groupes proto-aurignaciens à la grotte de l'Observatoire (principauté de Monaco). galip 52, 33–59. https://doi.org/10.3406/galip.2010.2470

Rebollo, N.R., Weiner, S., Brock, F., Meignen, L., Goldberg, P., Belfer-Cohen, A., Bar-Yosef, O., Boaretto, E., 2011. New radiocarbon dating of the transition from the Middle to the Upper Paleolithic in Kebara Cave, Israel. Journal of Archaeological Science 38, 2424–2433. https://doi.org/10.1016/j.jas.2011.05.010

Reimer, P.J., 2022. EVOLUTION OF RADIOCARBON CALIBRATION. Radiocarbon 64, 523–539. https://doi.org/10.1017/RDC.2021.62

Richter, J., Litt, T., Lehmkuhl, F., Hense, A., Hauck, T.C., Leder, D.F., Miebach, A., Parow-Souchon, H., Sauer, F., Schoenenberg, J., Al-Nahar, M., Hussain, S.T., 2020. Al-Ansab and the Dead Sea: Mid-MIS 3 archaeology and environment of the early Ahmarian population of the Levantine corridor. PLoS ONE 15, 1–36. https://doi.org/10.1371/journal.pone.0239968

Riel-Salvatore, J., Negrino, F., 2018. Proto-Aurignacian Lithic Technology, Mobility, and Human Niche Construction: A Case Study from Riparo Bombrini, Italy, in: Robinson, E., Sellet, F. (Eds.), Lithic Technological Organization and Paleoenvironmental Change. Springer International Publishing, Cham, pp. 163–187.

Roussel, M., Soressi, M., 2013. Une nouvelle séquence du Paléolithique supérieur ancien aux marges sud-ouest du Bassin parisien : les Cottés dans la Vienne, in: Bodu, P., Chehmana, L., Klaric, L., Mevel, L., Soriano, S., Teyssandier, N. (Eds.), Le Paléolithique Supérieur Ancien de l'Europe Du Nord-Ouest. Réflexions et Synthèses à Partir d'un Projet Collectif de Recherches Sur Le Paléolithique Supérieur Ancien Du Bassin Parisien, Journées SPF, Sens, 15-18 Avril (2009). Mémoire de la Société préhistorique française, Paris, pp. 283–298.

Santamaría Álvarez, D., 2013. La transición del Paleolítico medio al superior en Asturias: el abrigo de La Viña (La Manzaneda, Oviedo) y la cueva de El Sidrón (Borines, Piloña). Universidad de Oviedo, Oviedo.

Segre, A.G., Blanc, A.C., 1953. Excursion au Mont Circé, in: INQUA, IV International Congress. Roma-Pisa. pp. 87–106.

Sinitsyn, A.A., 2003. A Palaeolithic 'Pompeii' at Kostenki, Russia. Antiquity 77, 9–14. https://doi.org/10.1017/S0003598X00061299

Slimak, L., Pasesse, D., Giraud, Y., 2002. La grotte Mandrin et les premières occupations du Paléolithique supérieur en Occitanie orientale. ETFI 1. https://doi.org/10.5944/etfi.15.2002.4746

Tafelmaier, Y., 2017. Technological variability at the beginning of the Aurignacian in Northern Spain: Implications for the Proto- and Early Aurignacian distinction, Wissenschaftliche Schriften des Neanderthal Museums. Neanderthal Museum, Mettmann.

Tejero, J.-M., Grimaldi, S., 2015. Assessing bone and antler exploitation at Riparo Mochi (Balzi Rossi, Italy): implications for the characterization of the Aurignacian in South-western Europe. Journal of Archaeological Science 61, 59–77. https://doi.org/10.1016/j.jas.2015.05.003

Tsanova, T., Zwyns, N., Eizenberg, L., Teyssandier, N., Le Brun-Ricalens, F., Otte, M., 2012. Le plus petit dénominateur commun : réflexion sur la variabilité des ensembles lamellaires du Paléolithique supérieur ancien d'Eurasie. Un bilan autour des exemples de Kozarnika (Est des Balkans) et Yafteh (Zagros central). L'Anthropologie 116, 469–509. https://doi.org/10.1016/j.anthro.2011.10.005

Weinstein, J.M., 1984. Radiocarbon Dating in the Southern Levant. Radiocarbon 26, 297–366. https://doi.org/10.1017/S0033822200006731
