# Supplementary figures and images for: Tracking the emergence of the Upper Palaeolithic in western Asia and Europe: A Multiple Correspondence Analysis of Protoaurignacian and Southern Ahmarian lithics
Source: PLoS One. 2025 Sep 24;20(9):e0331393. doi: 10.1371/journal.pone.0331393 (PMC12459816; doi:10.1371/journal.pone.0331393)

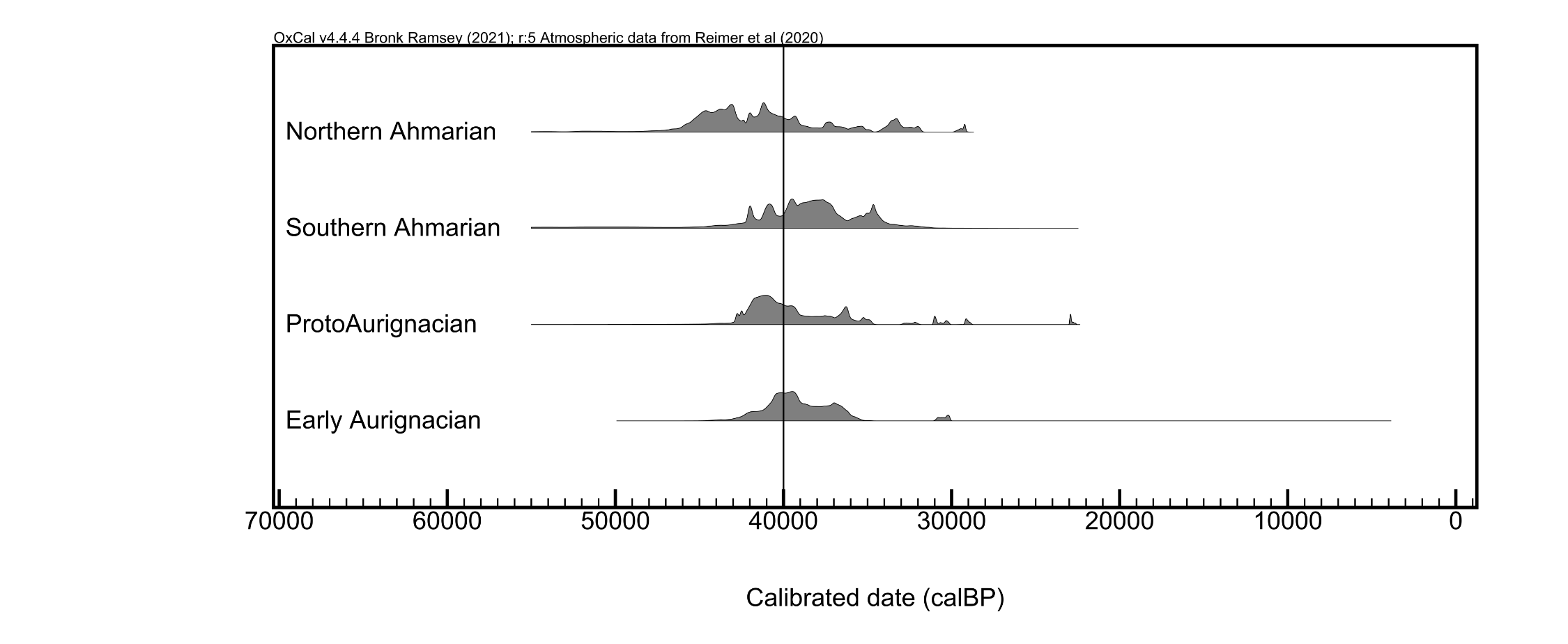

Supplement: S1 Fig — (ZIP) [file pone.0331393.s004.zip › Supporting_Information_Figures/SI_Fig64_EUP_dates.tiff]

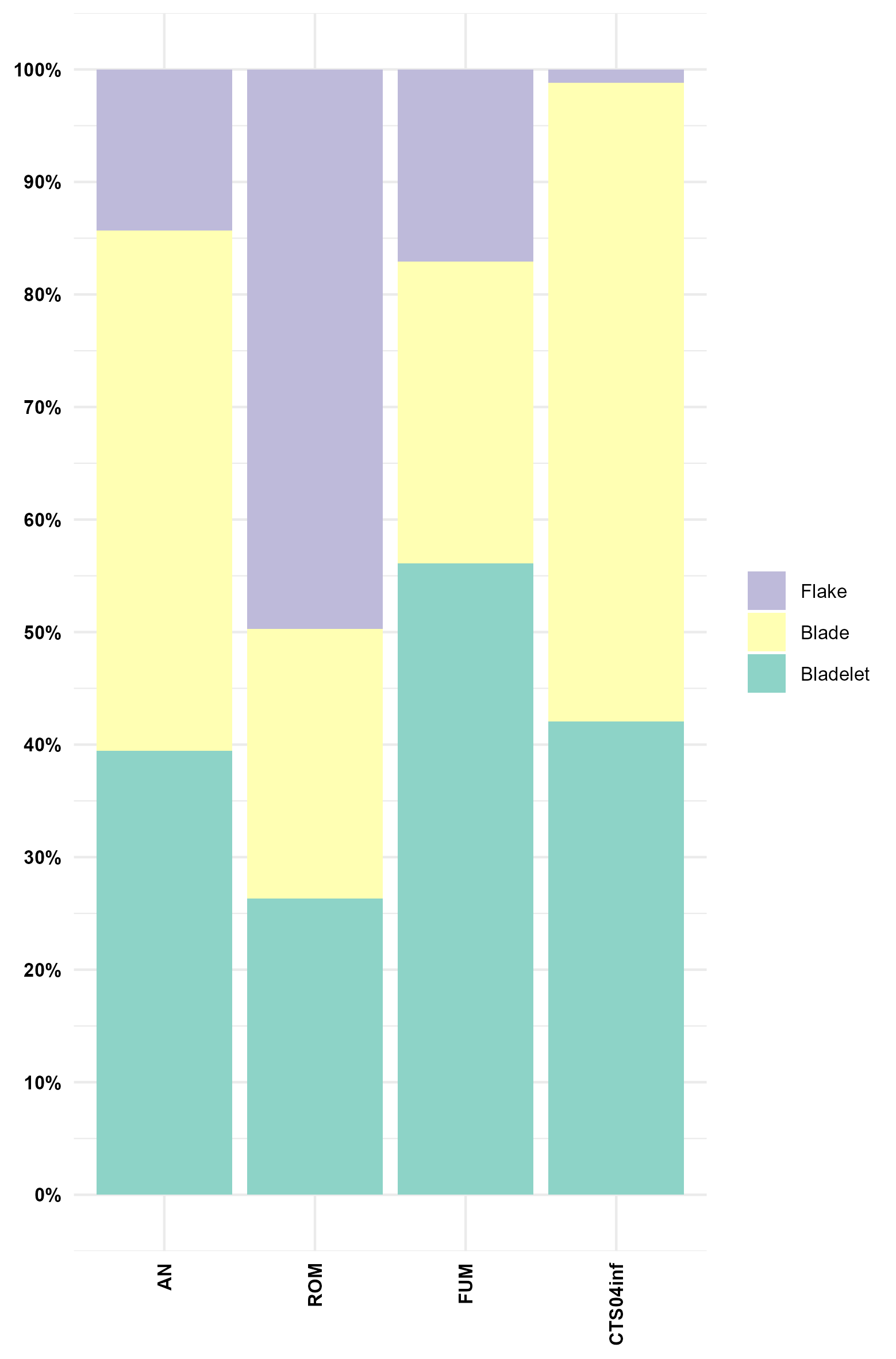

Supplement: S1 Fig — (ZIP) [file pone.0331393.s004.zip › Supporting_Information_Figures/SI_Figures_Exploratory-Plots/SIFig01_Blanks.tiff]

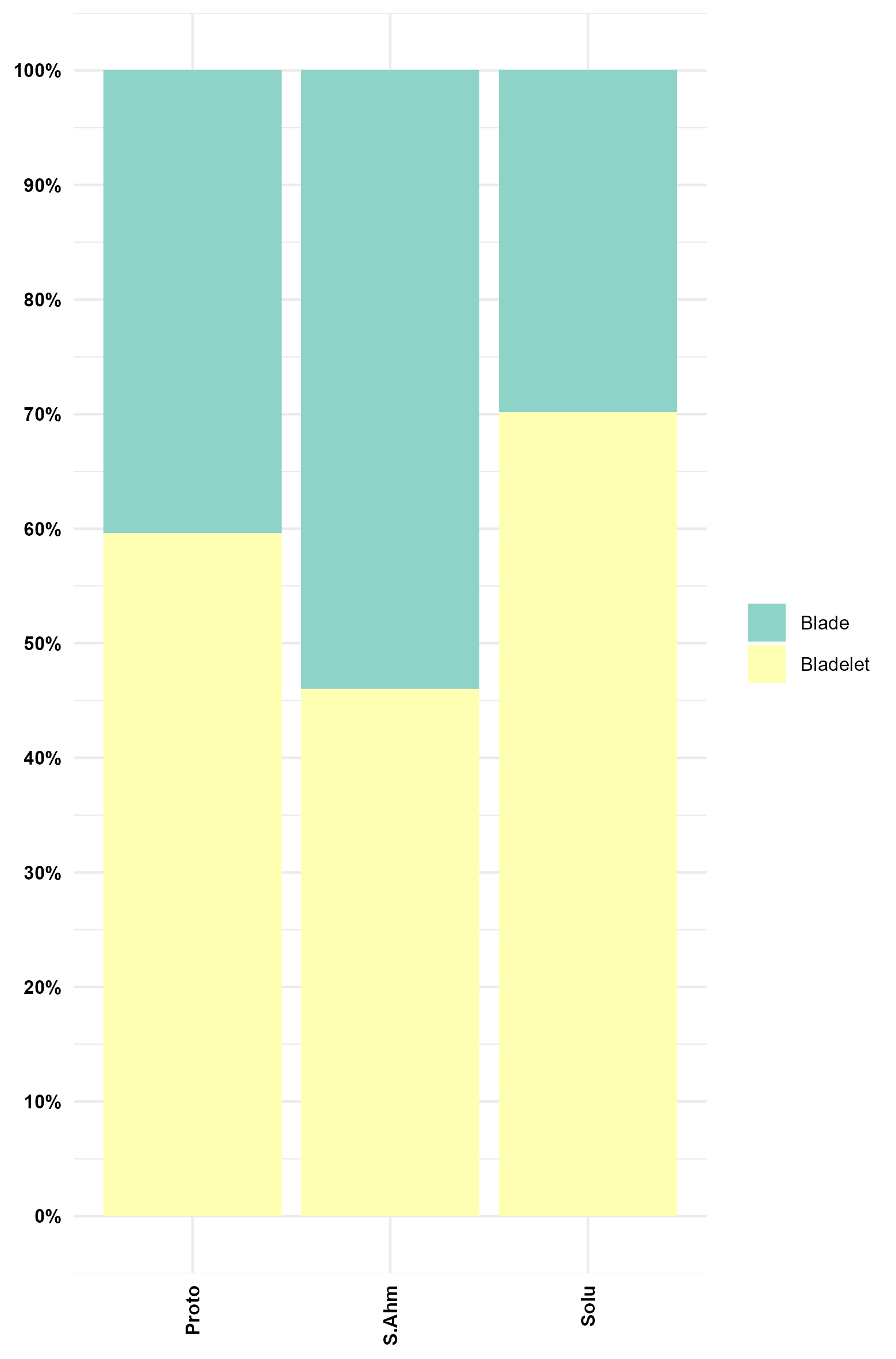

Supplement: S1 Fig — (ZIP) [file pone.0331393.s004.zip › Supporting_Information_Figures/SI_Figures_Exploratory-Plots/SIFig02_BlanksEUP-Solu.tiff]

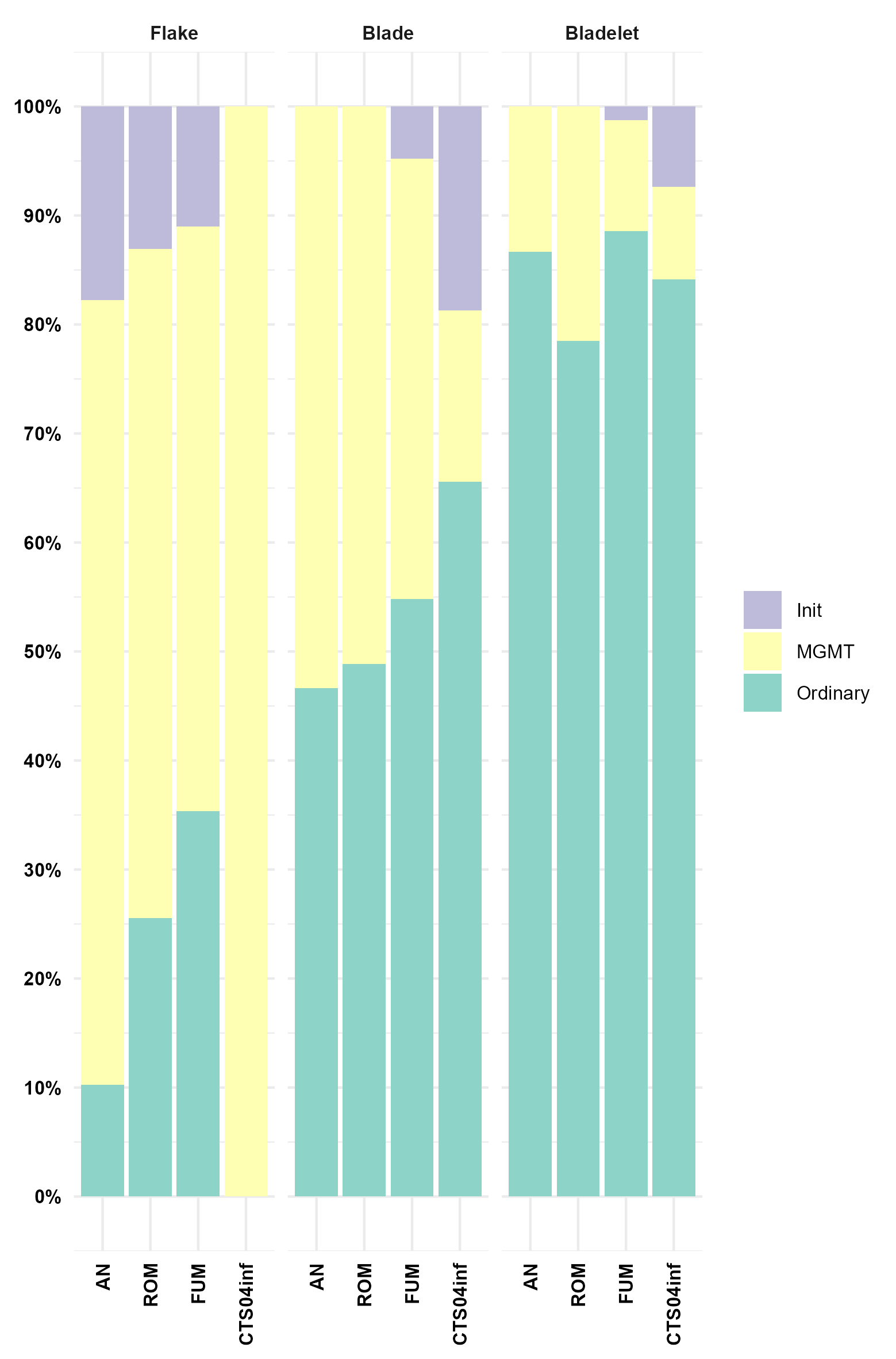

Supplement: S1 Fig — (ZIP) [file pone.0331393.s004.zip › Supporting_Information_Figures/SI_Figures_Exploratory-Plots/SIFig03_TechPhase.tiff]

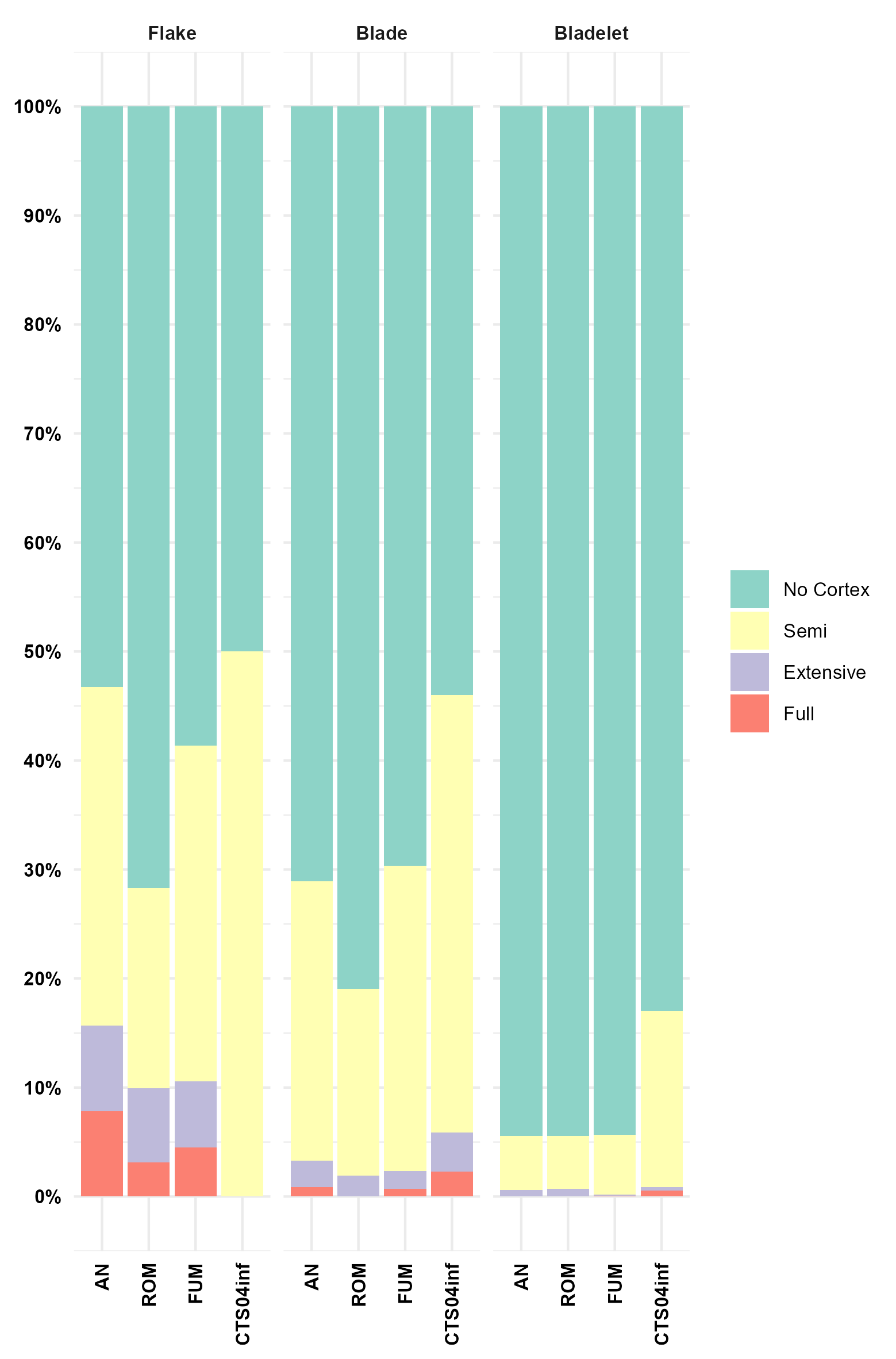

Supplement: S1 Fig — (ZIP) [file pone.0331393.s004.zip › Supporting_Information_Figures/SI_Figures_Exploratory-Plots/SIFig04_Cortex.tiff]

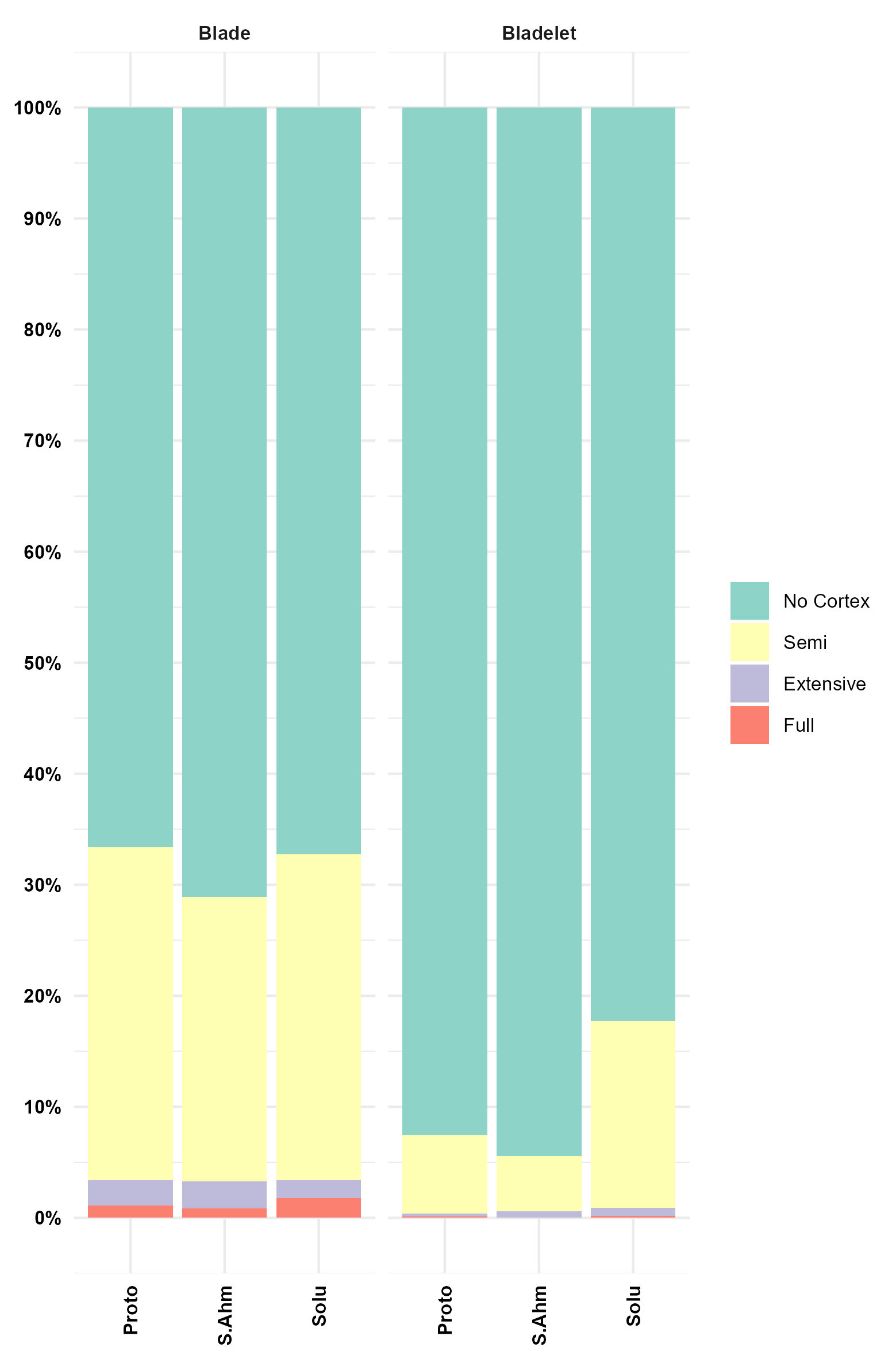

Supplement: S1 Fig — (ZIP) [file pone.0331393.s004.zip › Supporting_Information_Figures/SI_Figures_Exploratory-Plots/SIFig05_CortexEUP-Solu.tiff]

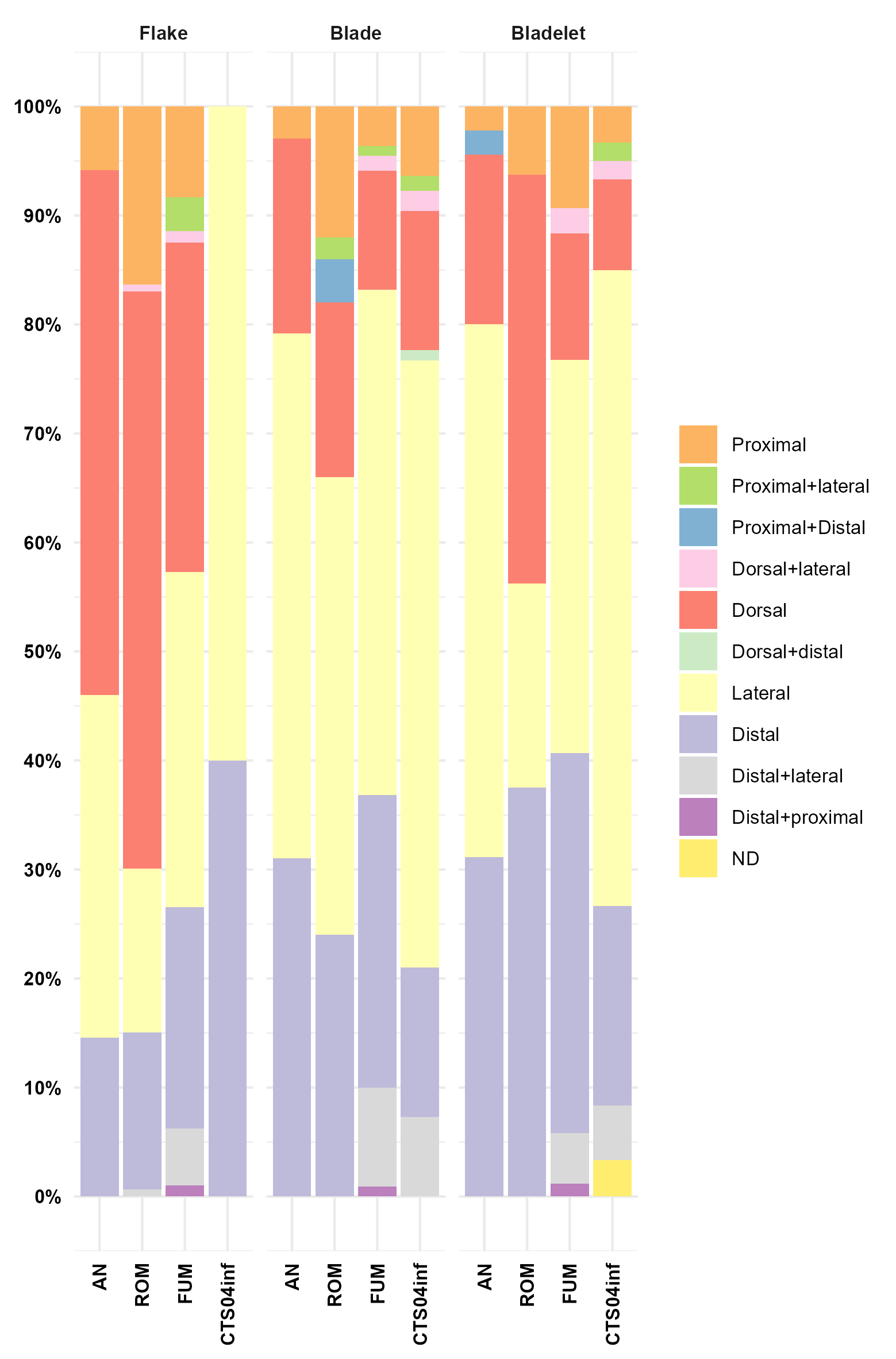

Supplement: S1 Fig — (ZIP) [file pone.0331393.s004.zip › Supporting_Information_Figures/SI_Figures_Exploratory-Plots/SIFig06_CortexPos.tiff]

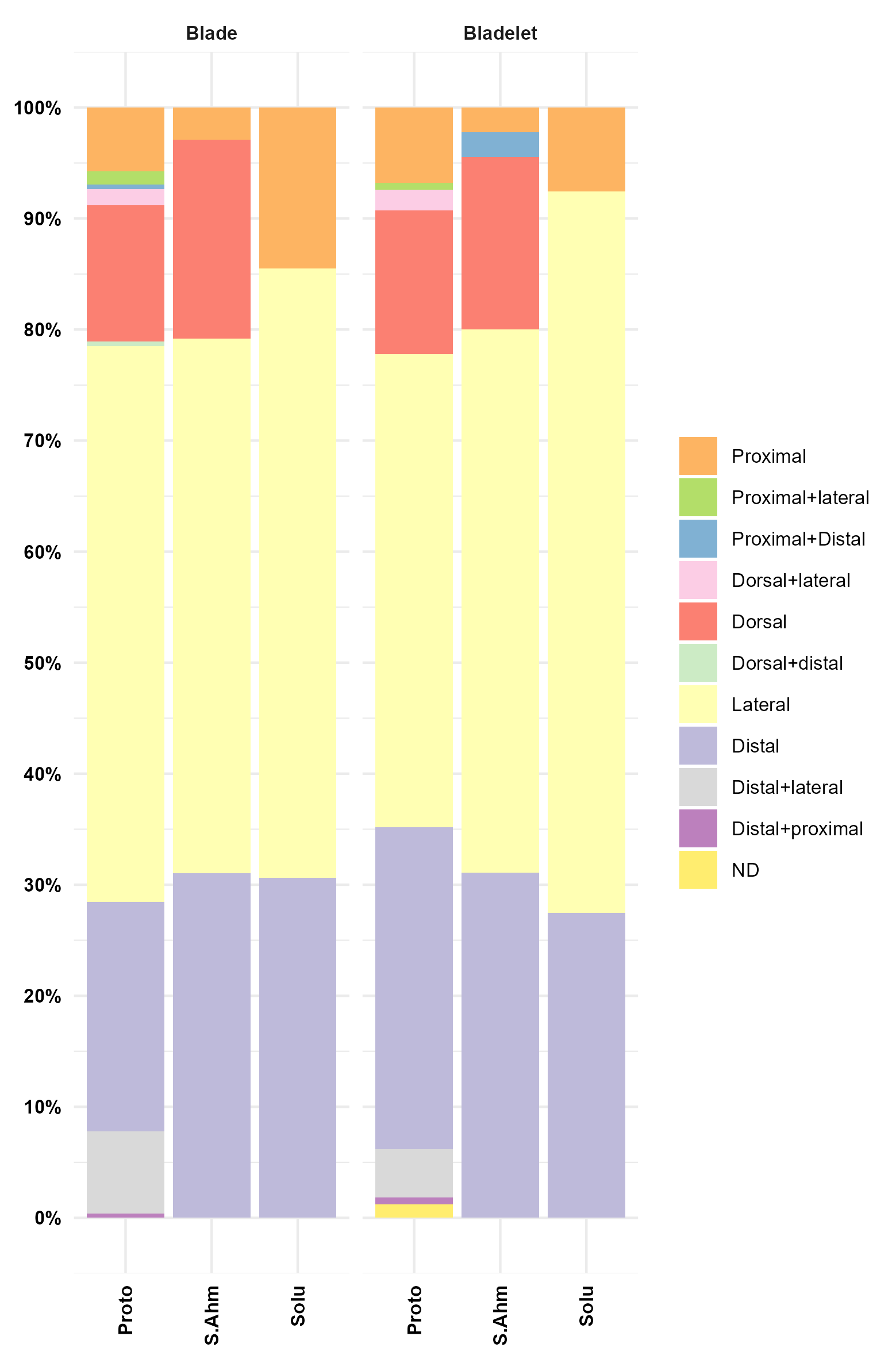

Supplement: S1 Fig — (ZIP) [file pone.0331393.s004.zip › Supporting_Information_Figures/SI_Figures_Exploratory-Plots/SIFig07_CortexPos EUP-Solu.tiff]

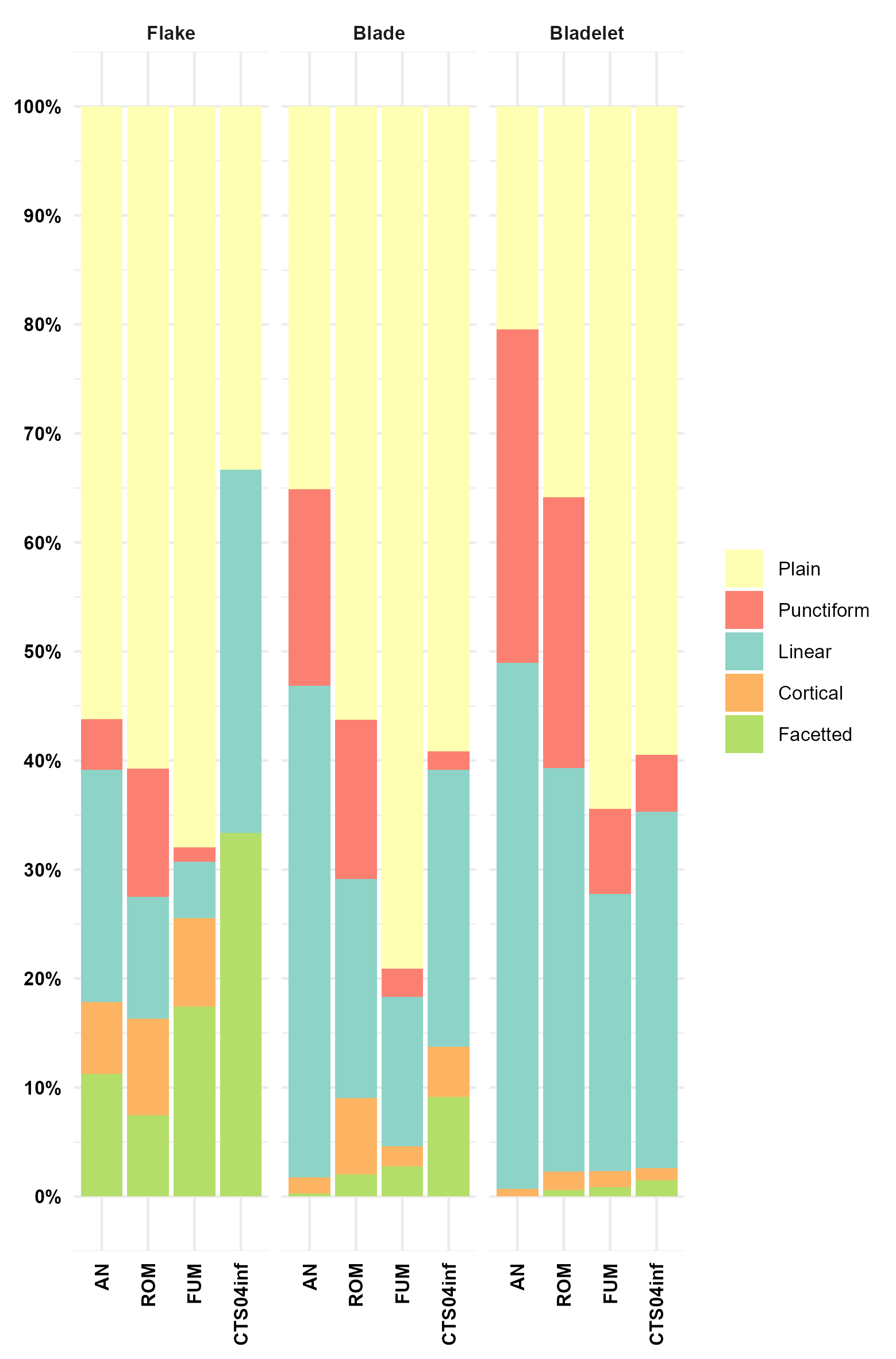

Supplement: S1 Fig — (ZIP) [file pone.0331393.s004.zip › Supporting_Information_Figures/SI_Figures_Exploratory-Plots/SIFig08_Platform.tiff]

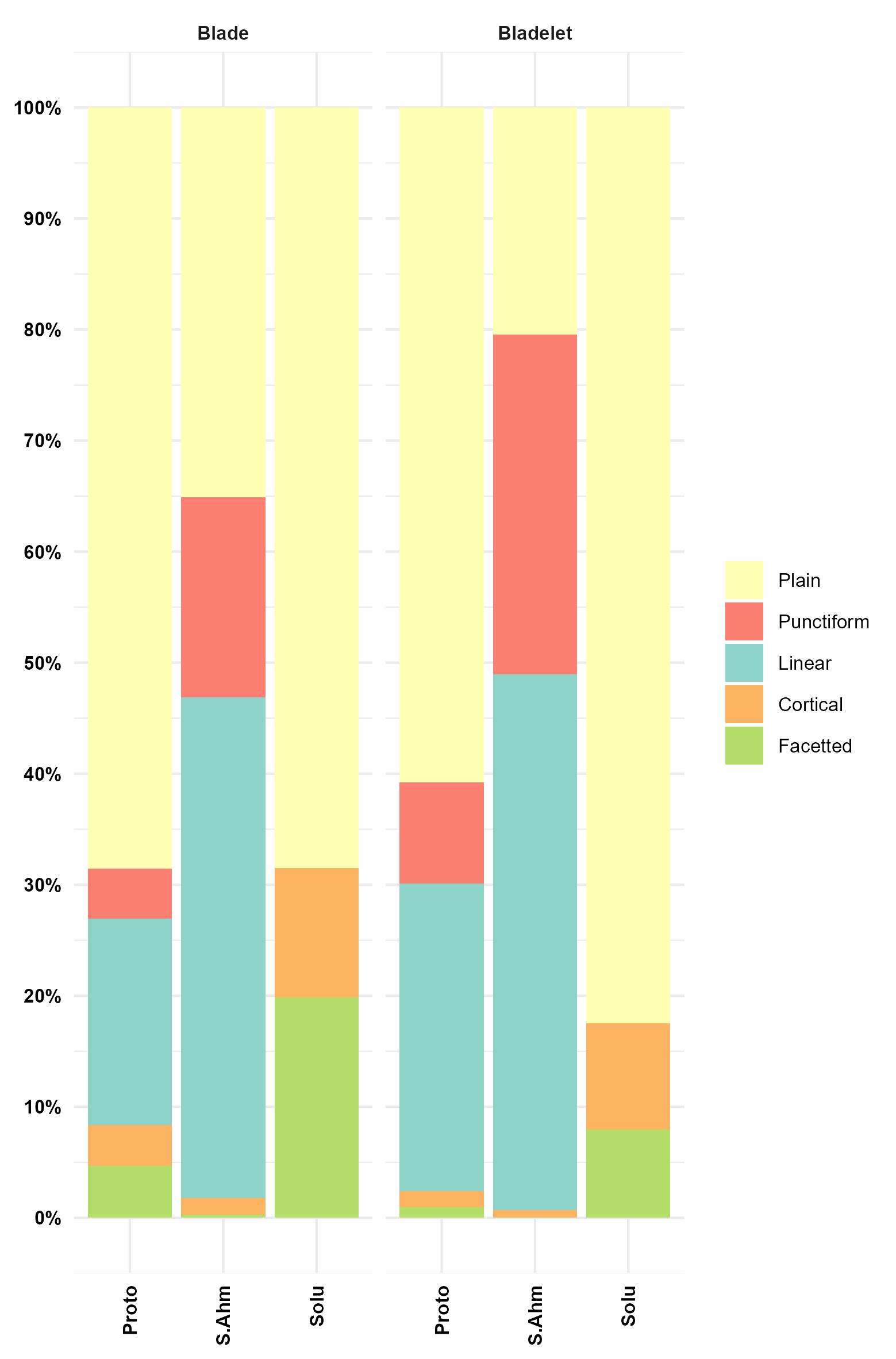

Supplement: S1 Fig — (ZIP) [file pone.0331393.s004.zip › Supporting_Information_Figures/SI_Figures_Exploratory-Plots/SIFig09_Platform EUP-Solu.tiff]

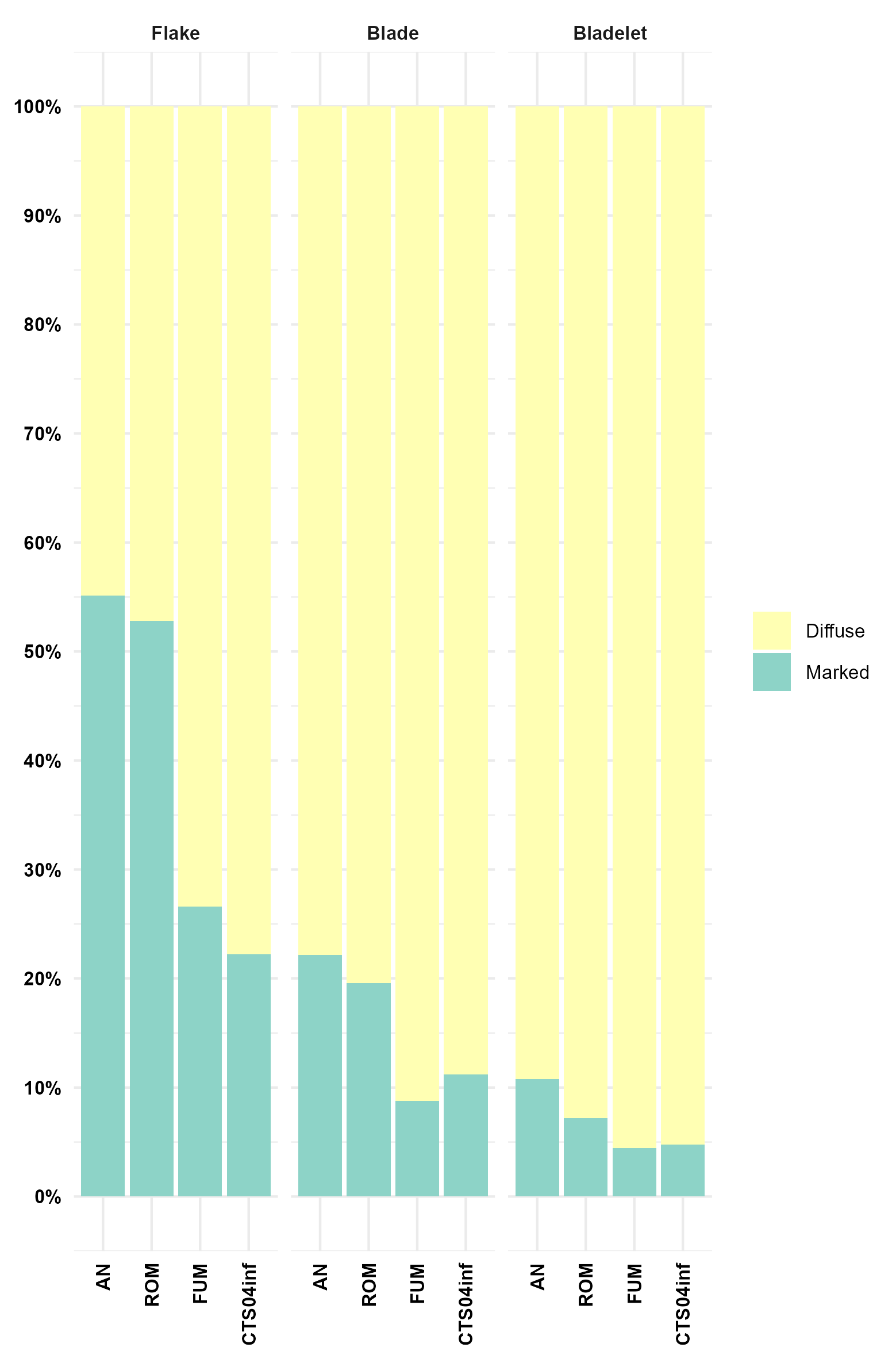

Supplement: S1 Fig — (ZIP) [file pone.0331393.s004.zip › Supporting_Information_Figures/SI_Figures_Exploratory-Plots/SIFig10_Bulb.tiff]

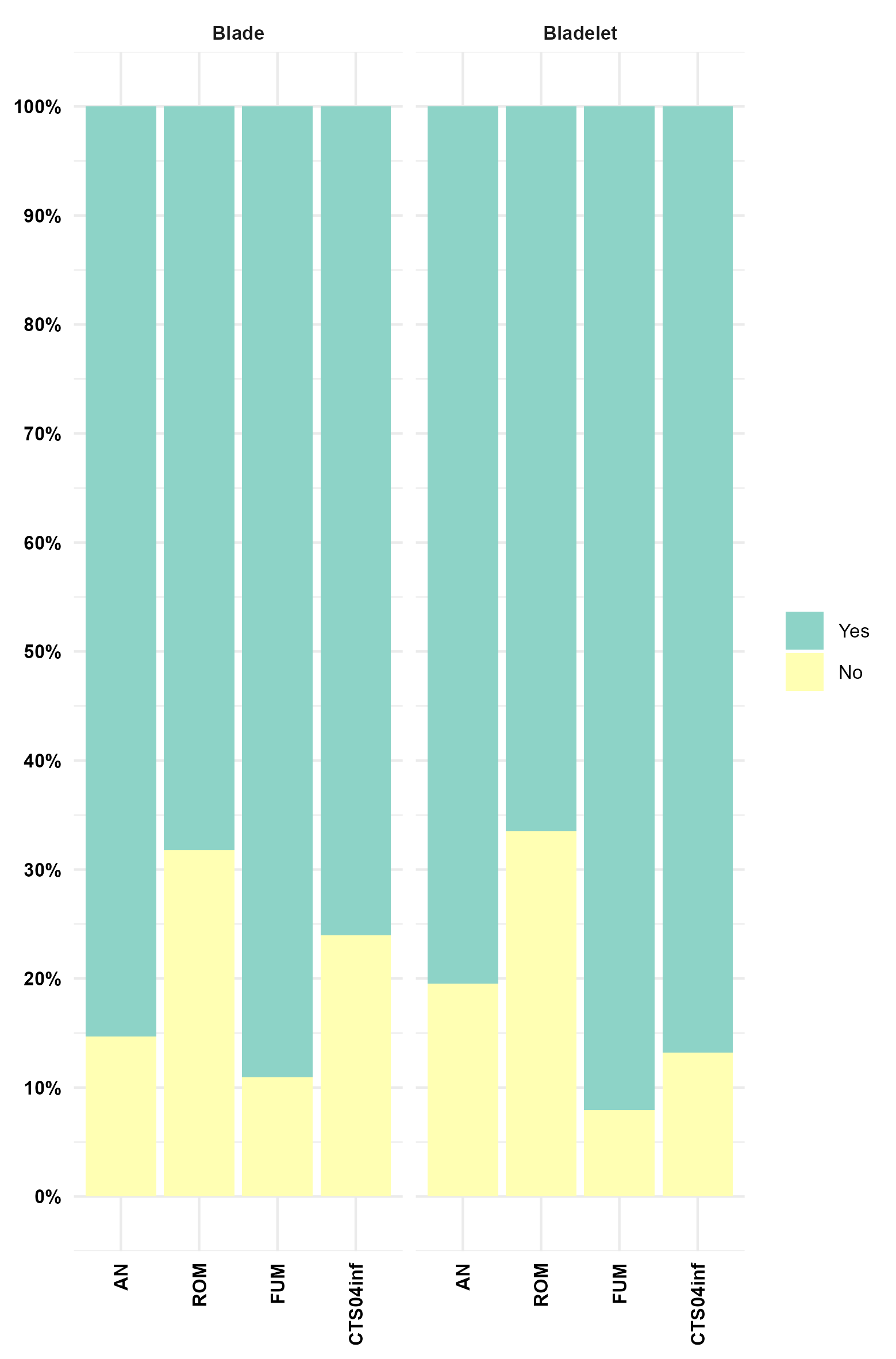

Supplement: S1 Fig — (ZIP) [file pone.0331393.s004.zip › Supporting_Information_Figures/SI_Figures_Exploratory-Plots/SIFig11_Abrasion.tiff]

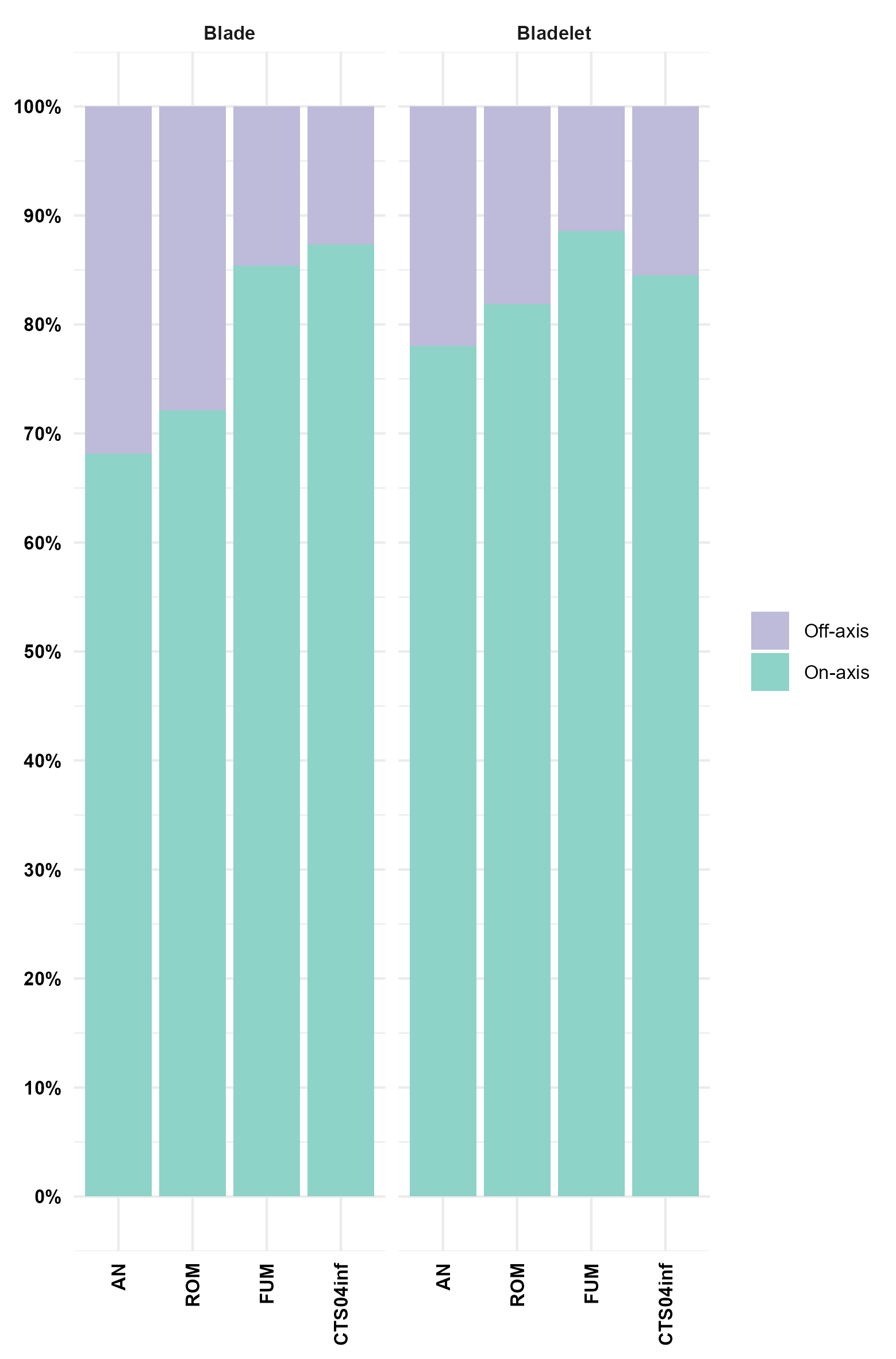

Supplement: S1 Fig — (ZIP) [file pone.0331393.s004.zip › Supporting_Information_Figures/SI_Figures_Exploratory-Plots/SIFig12_Axiality.tiff]

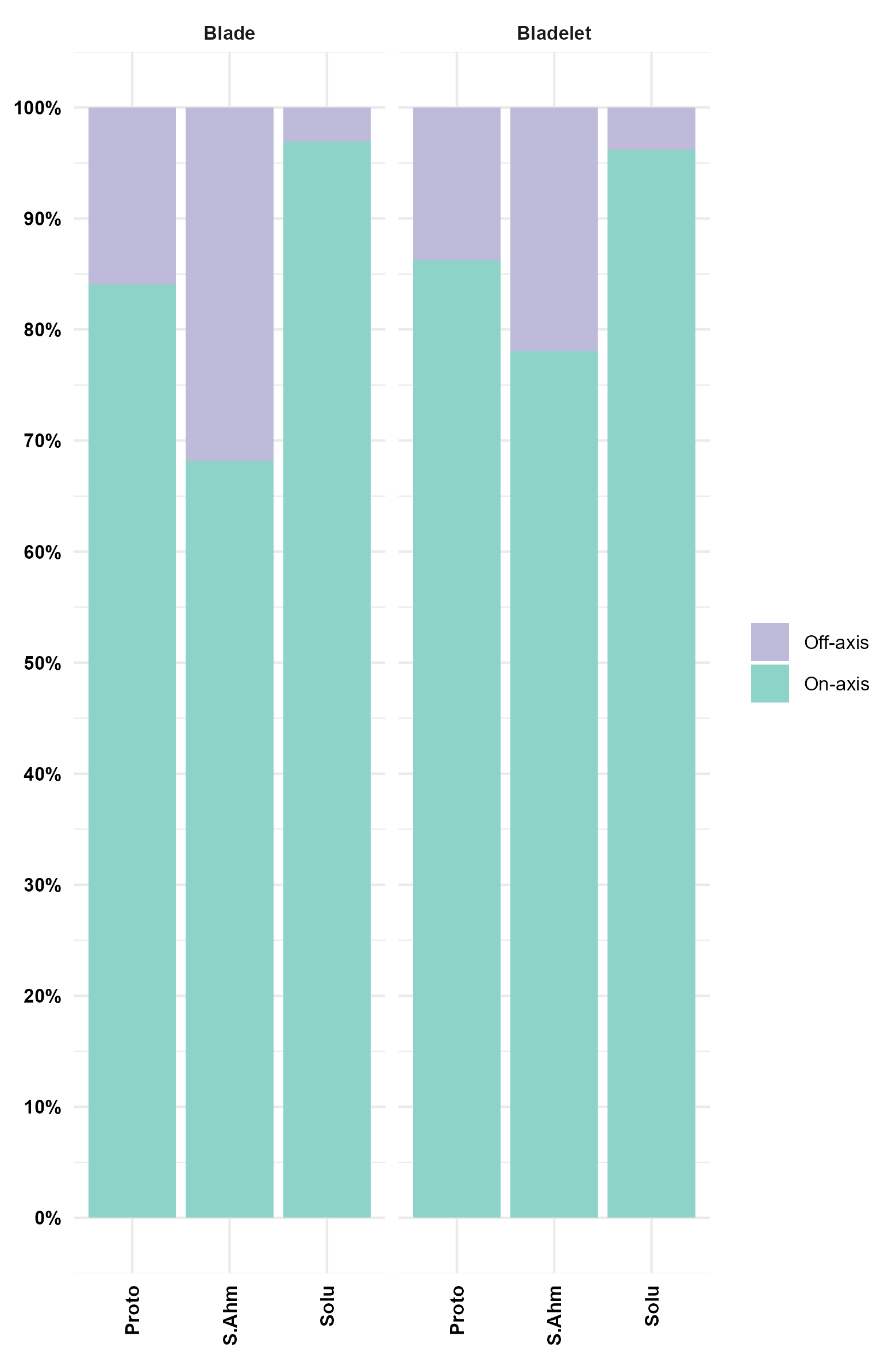

Supplement: S1 Fig — (ZIP) [file pone.0331393.s004.zip › Supporting_Information_Figures/SI_Figures_Exploratory-Plots/SIFig13_Axility EUP-Solu.tiff]

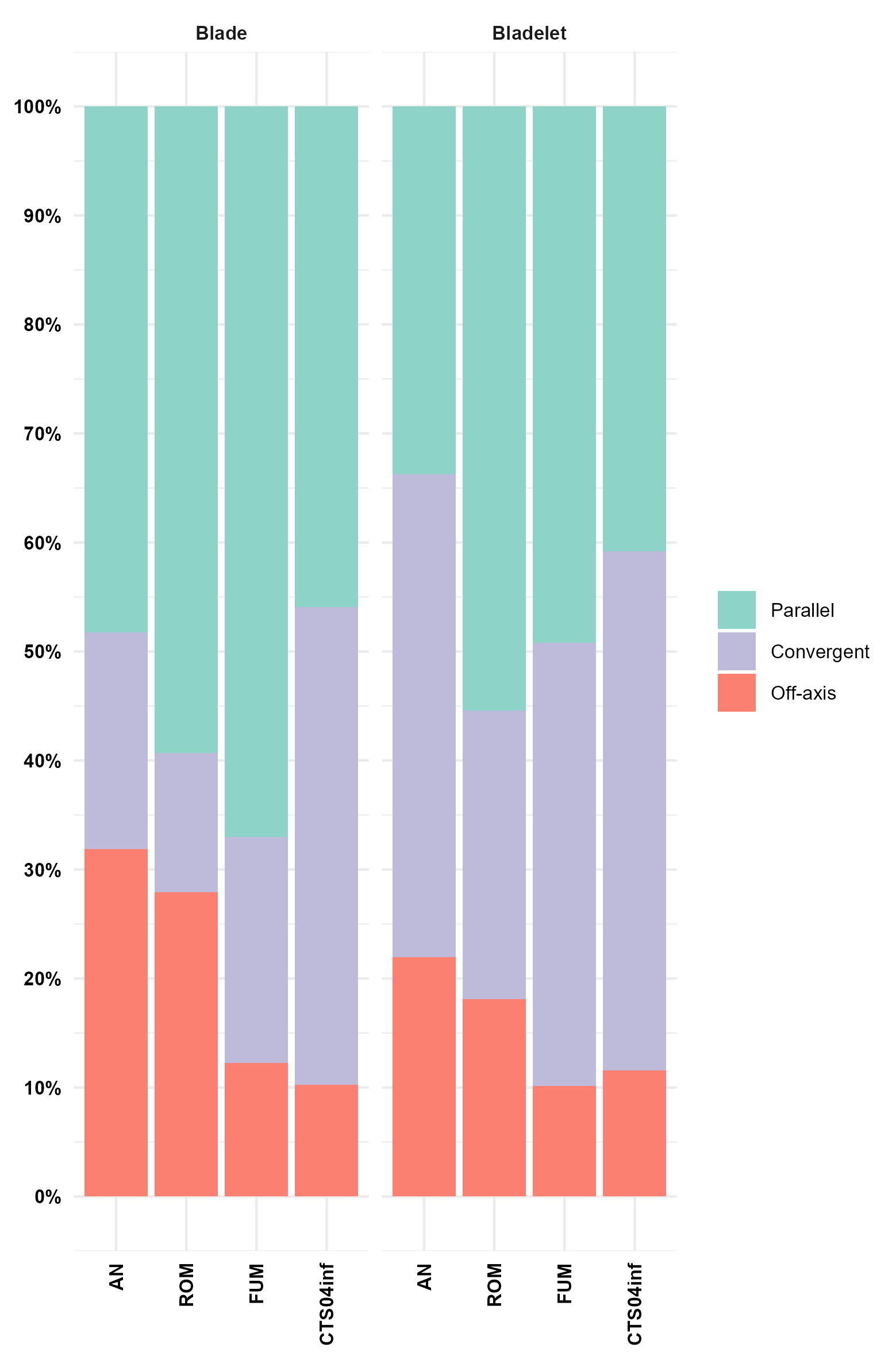

Supplement: S1 Fig — (ZIP) [file pone.0331393.s004.zip › Supporting_Information_Figures/SI_Figures_Exploratory-Plots/SIFig14_OutMorph.tiff]

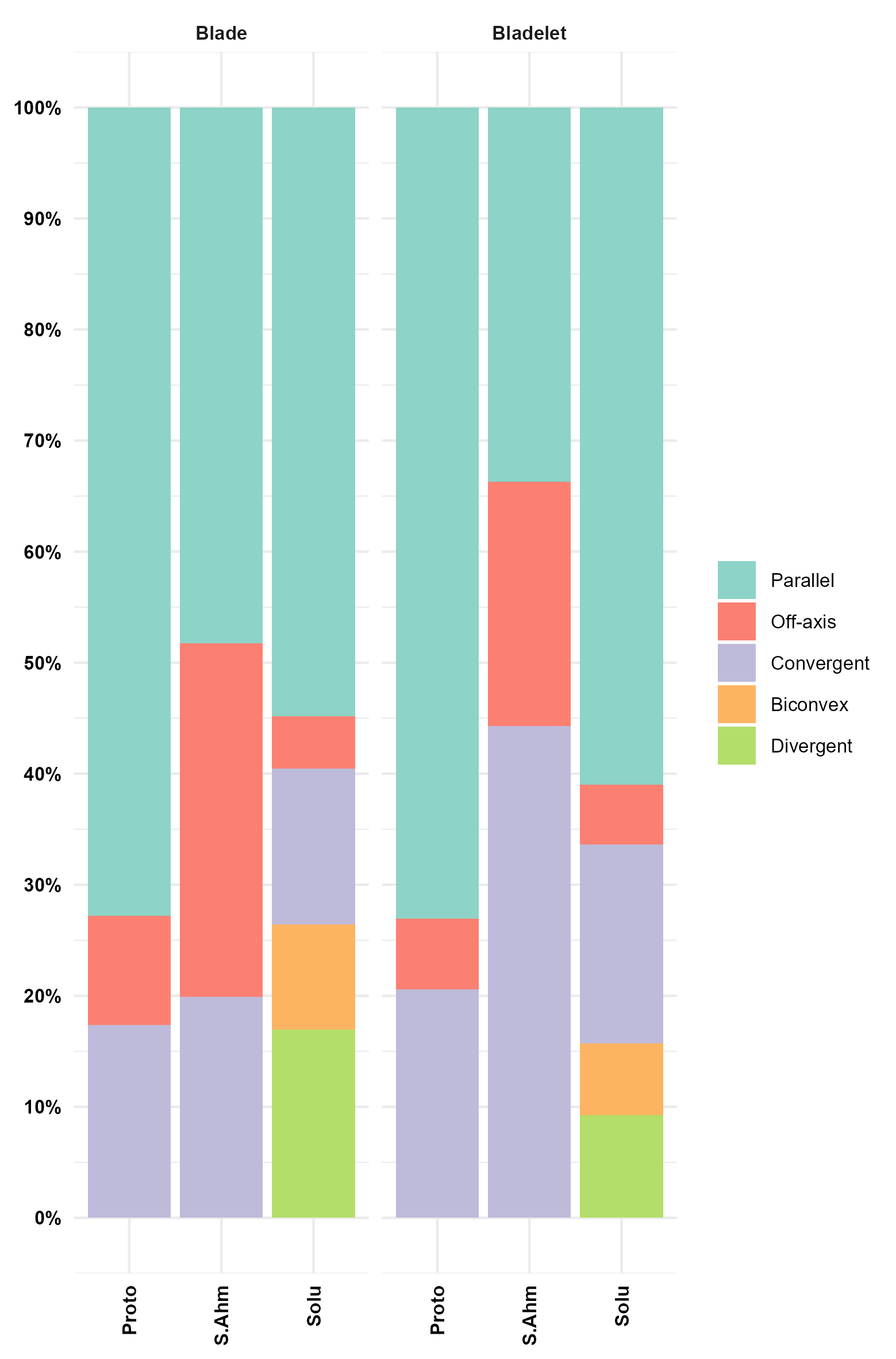

Supplement: S1 Fig — (ZIP) [file pone.0331393.s004.zip › Supporting_Information_Figures/SI_Figures_Exploratory-Plots/SIFig15_OutMorph EUP-Solu.tiff]

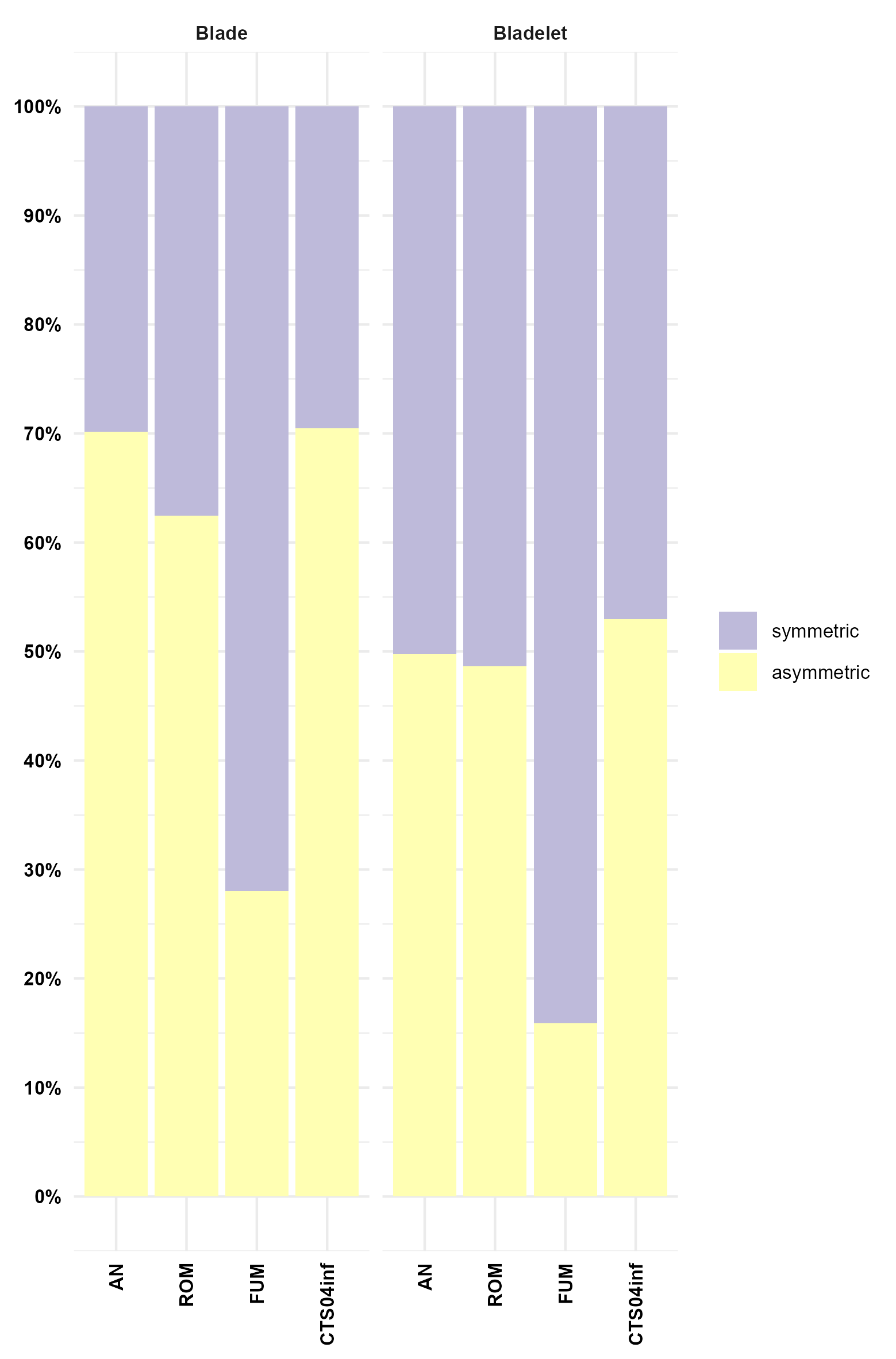

Supplement: S1 Fig — (ZIP) [file pone.0331393.s004.zip › Supporting_Information_Figures/SI_Figures_Exploratory-Plots/SIFig16_Symmetry.tiff]

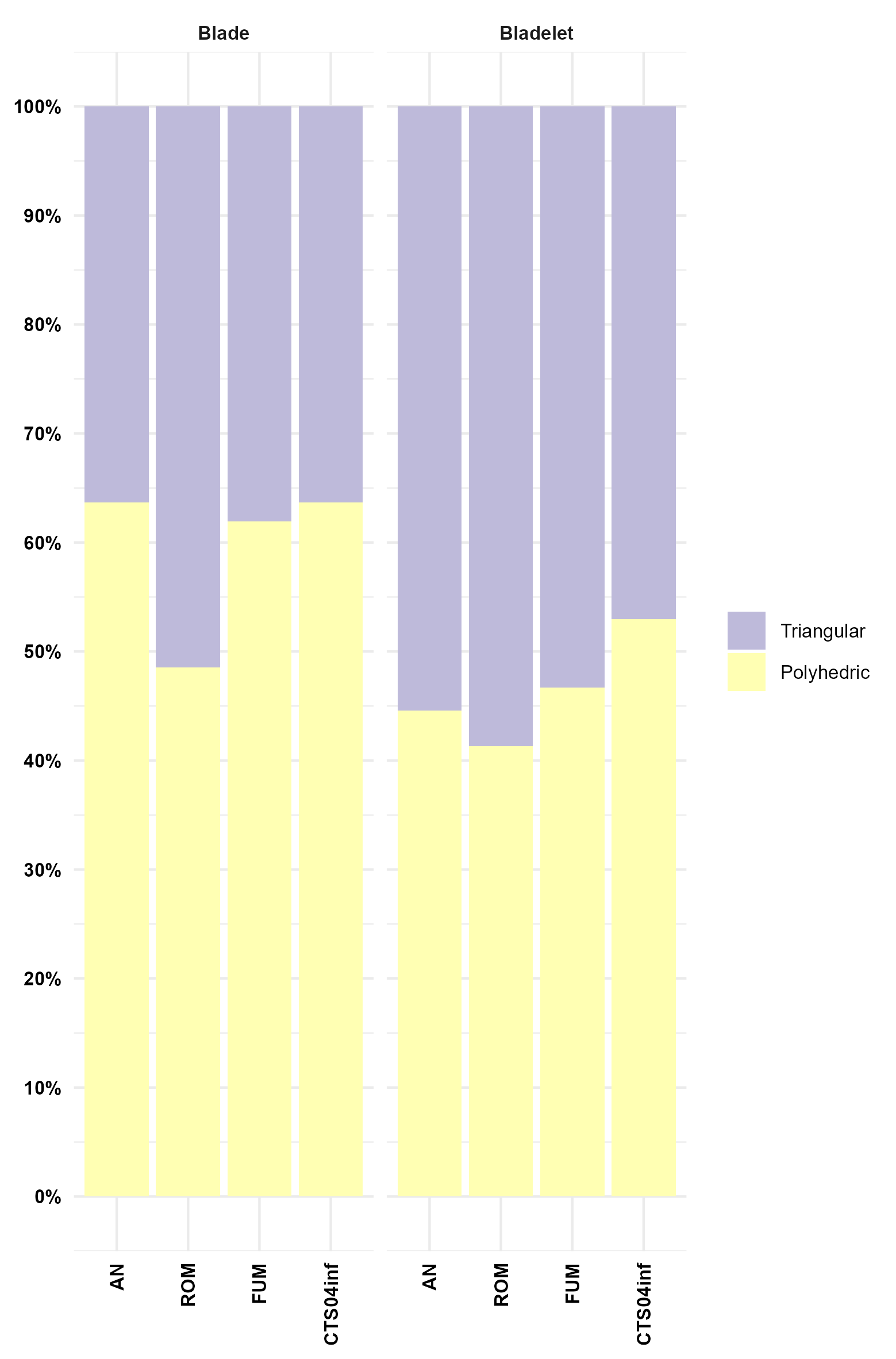

Supplement: S1 Fig — (ZIP) [file pone.0331393.s004.zip › Supporting_Information_Figures/SI_Figures_Exploratory-Plots/SIFig17_CrossSection.tiff]

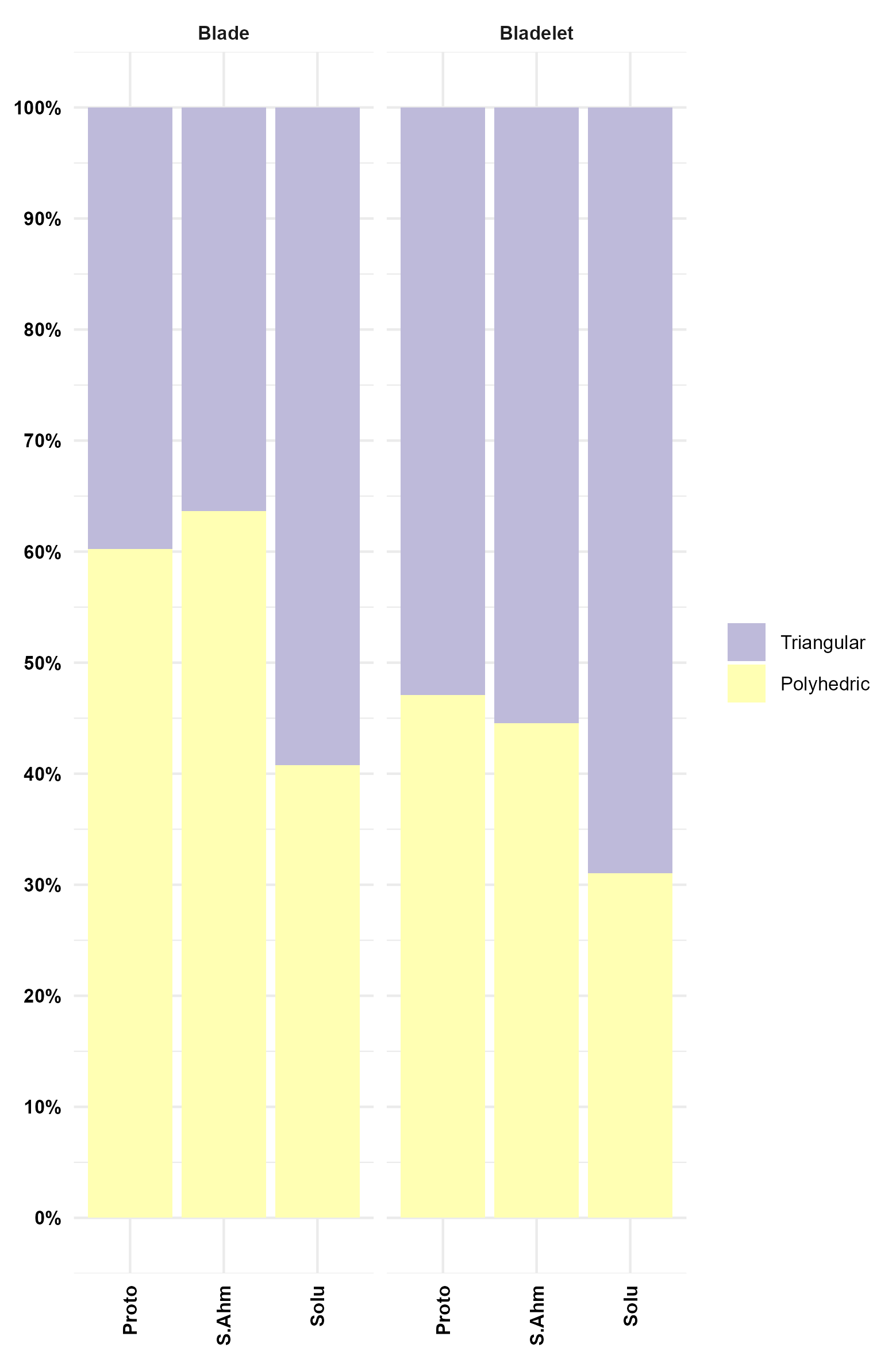

Supplement: S1 Fig — (ZIP) [file pone.0331393.s004.zip › Supporting_Information_Figures/SI_Figures_Exploratory-Plots/SIFig18_CrossSection EUP-Solu.tiff]

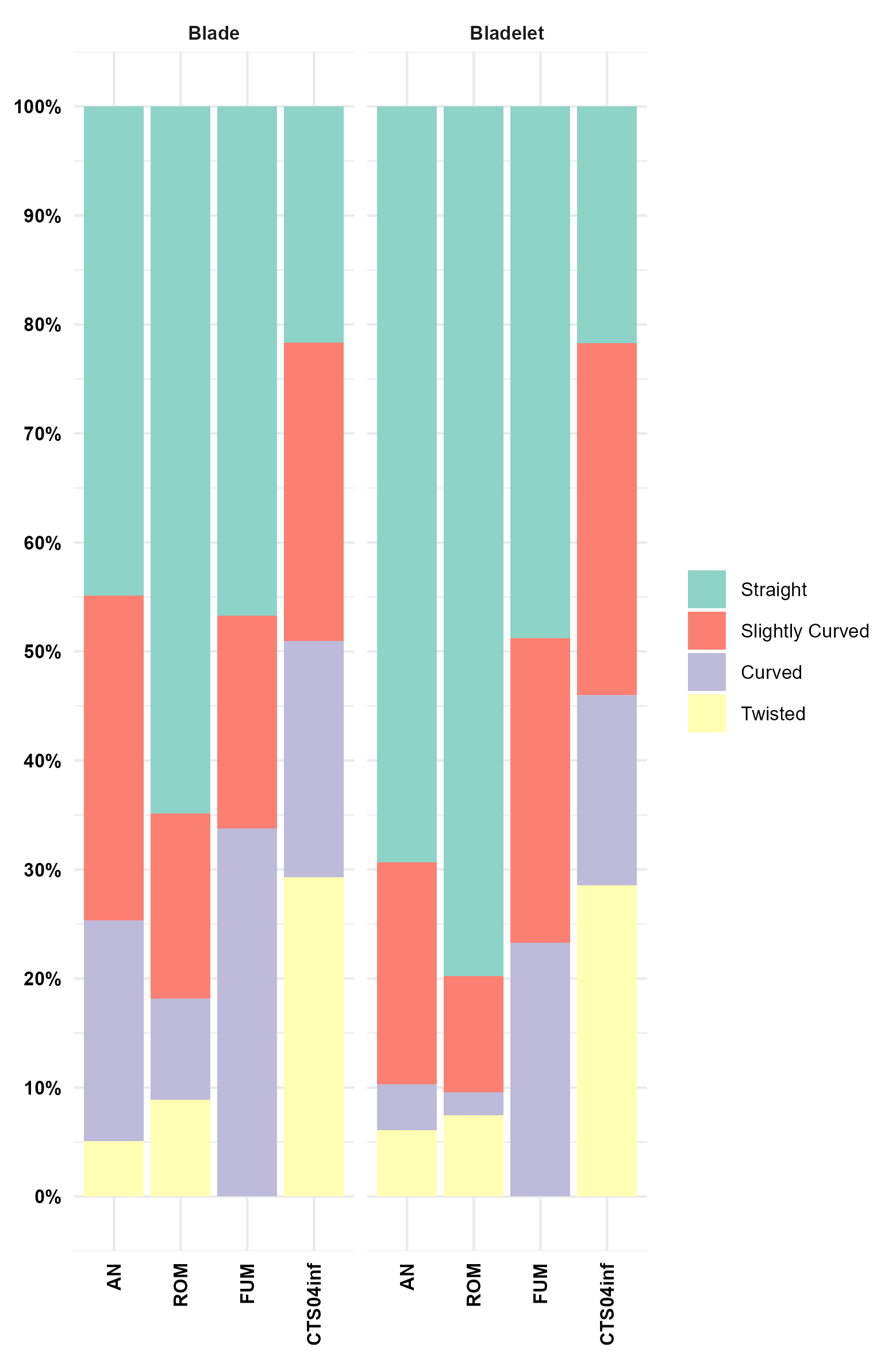

Supplement: S1 Fig — (ZIP) [file pone.0331393.s004.zip › Supporting_Information_Figures/SI_Figures_Exploratory-Plots/SIFig19_Profile.tiff]

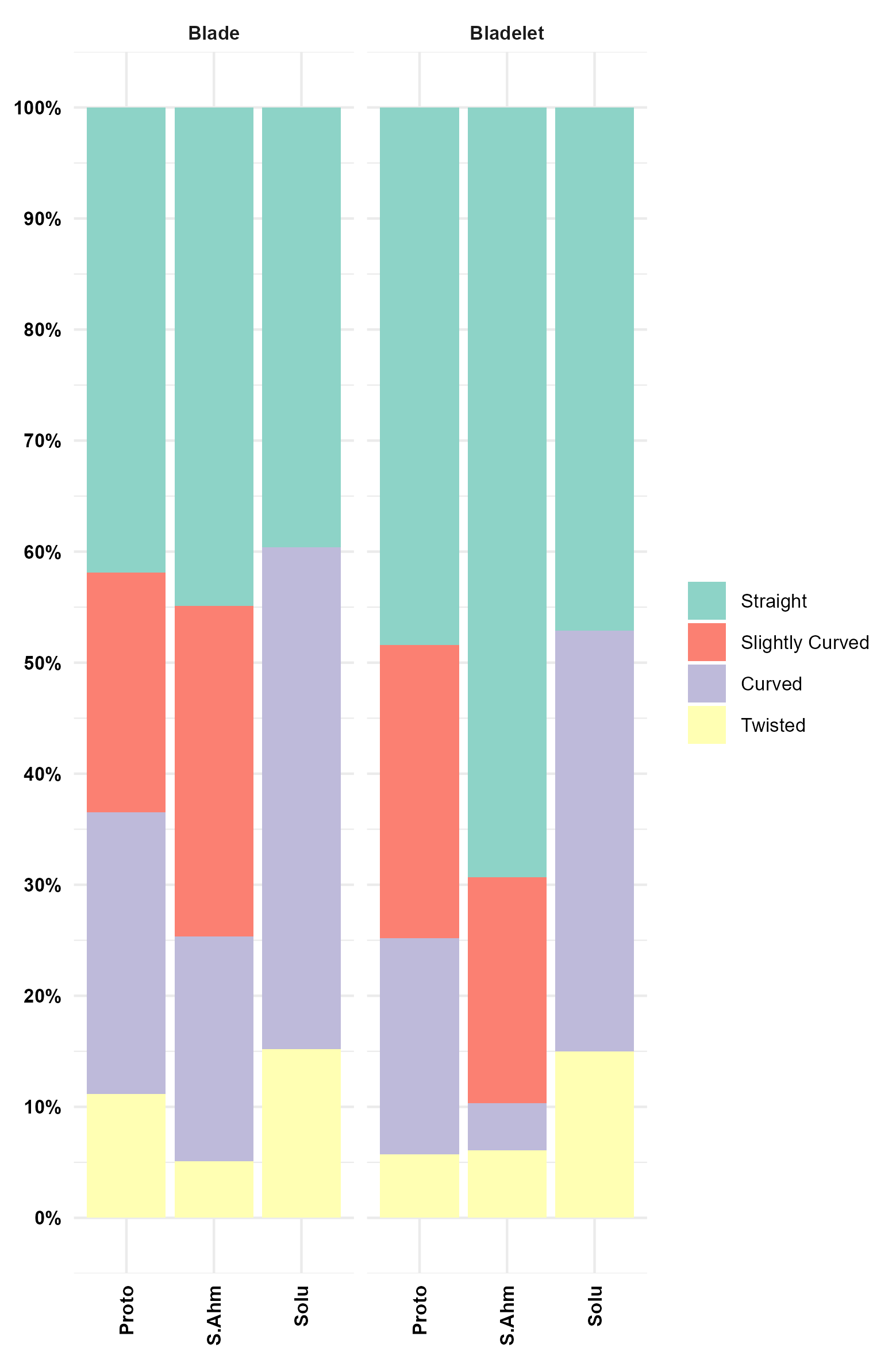

Supplement: S1 Fig — (ZIP) [file pone.0331393.s004.zip › Supporting_Information_Figures/SI_Figures_Exploratory-Plots/SIFig20_Profile EUP-Solu.tiff]

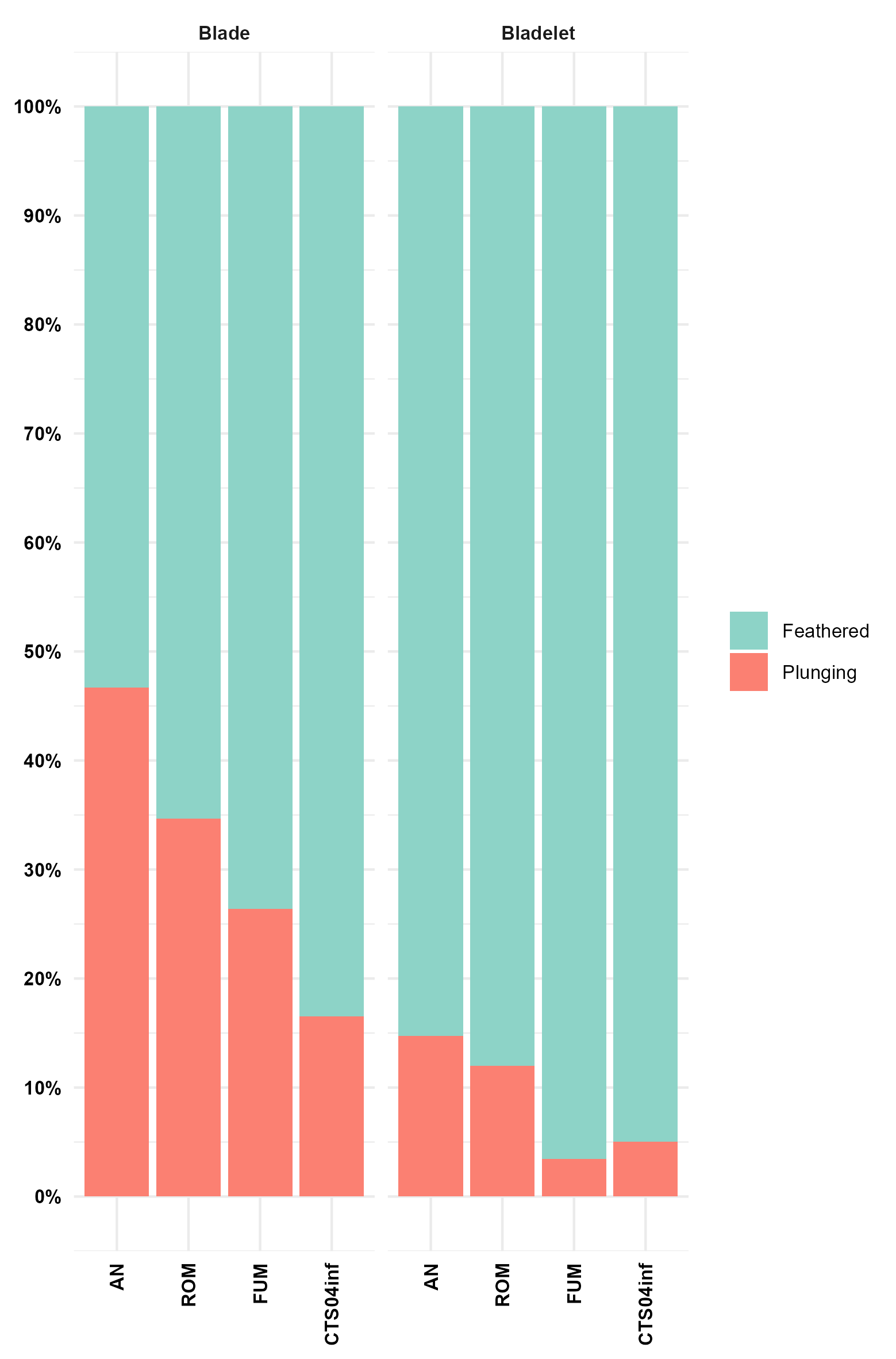

Supplement: S1 Fig — (ZIP) [file pone.0331393.s004.zip › Supporting_Information_Figures/SI_Figures_Exploratory-Plots/SIFig21_Distal edn morpho.tiff]

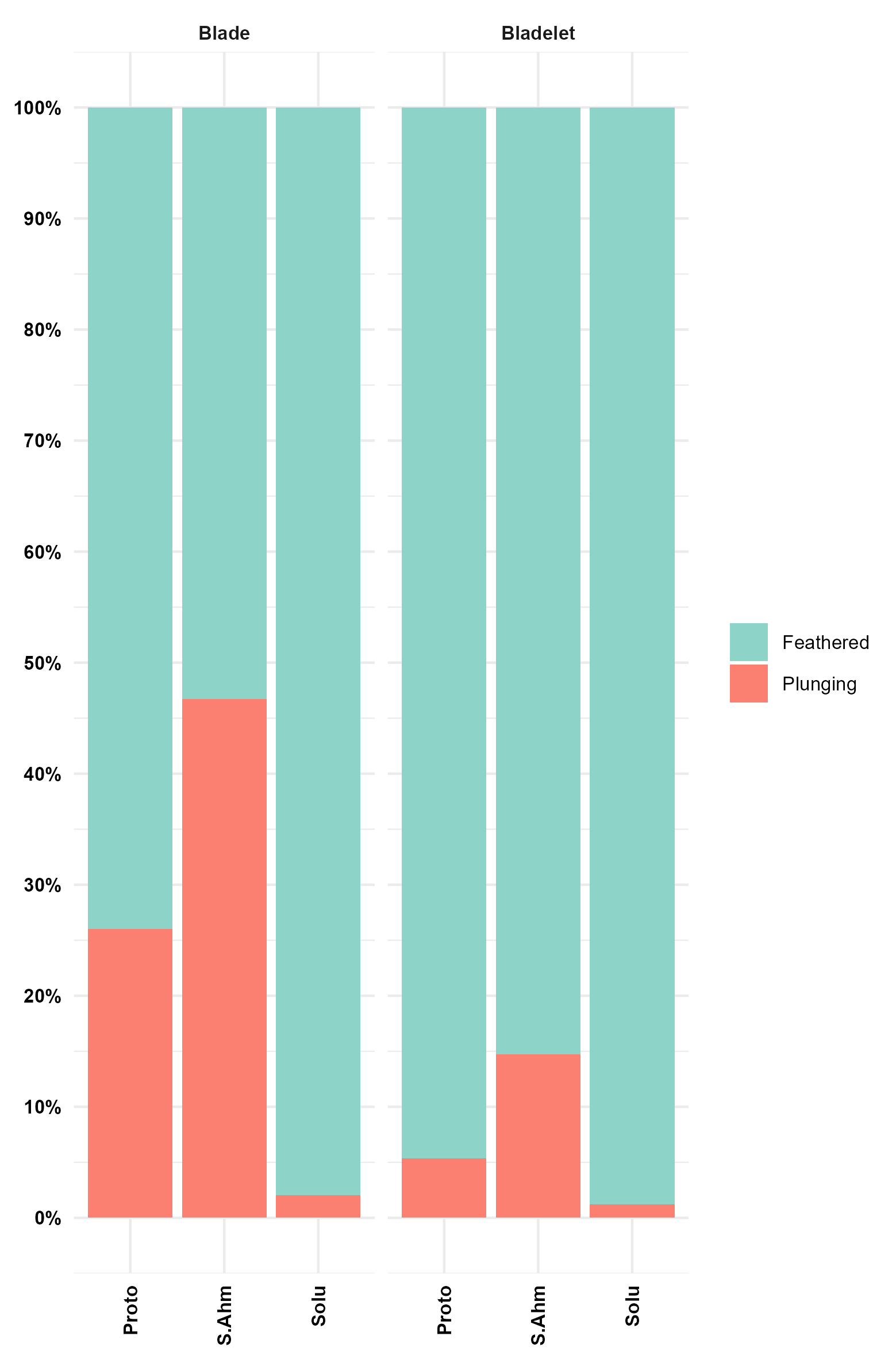

Supplement: S1 Fig — (ZIP) [file pone.0331393.s004.zip › Supporting_Information_Figures/SI_Figures_Exploratory-Plots/SIFig22_Distal end moprho EUP-Solu.tiff]

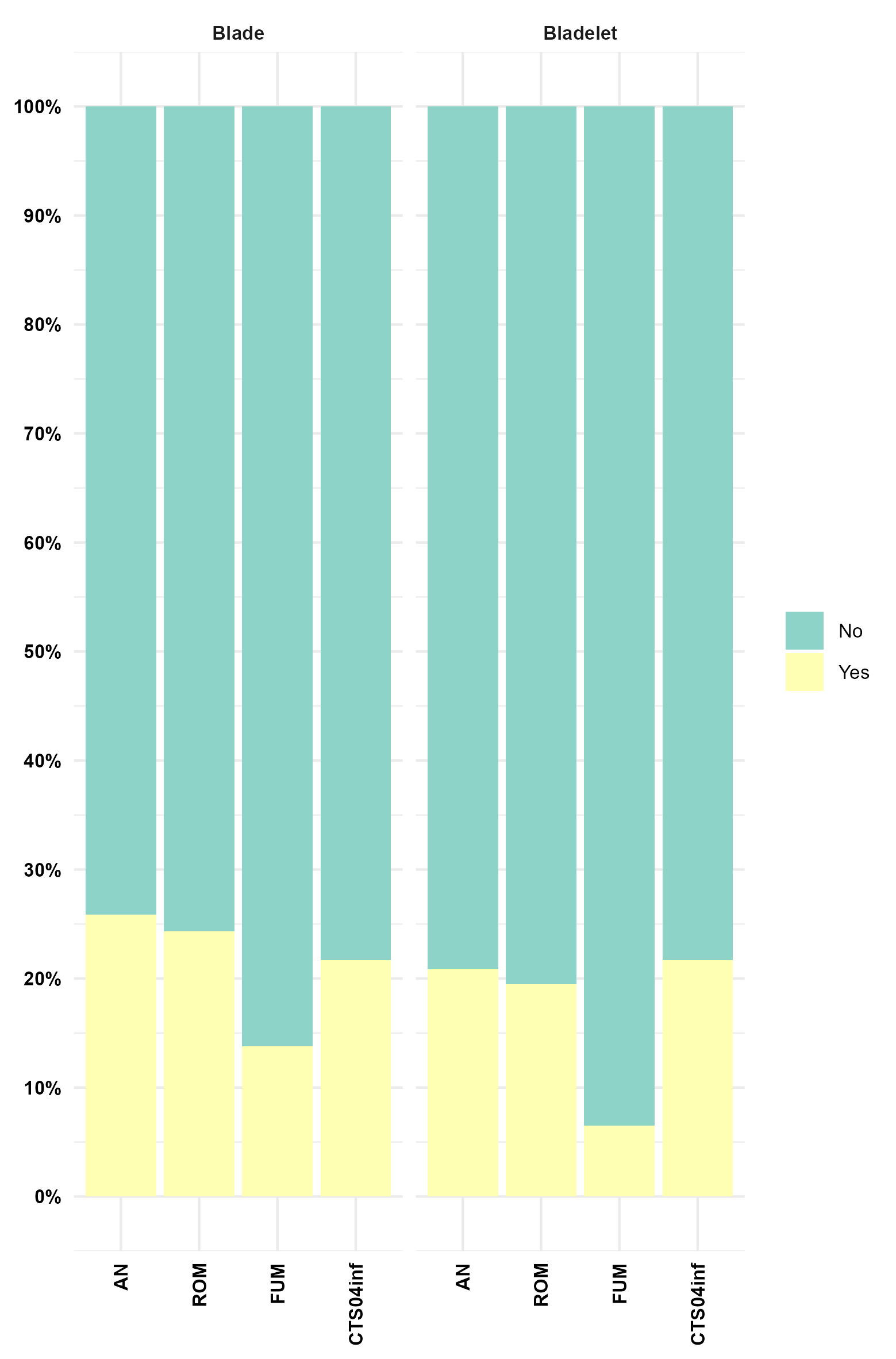

Supplement: S1 Fig — (ZIP) [file pone.0331393.s004.zip › Supporting_Information_Figures/SI_Figures_Exploratory-Plots/SIFig23_Torsion.tiff]

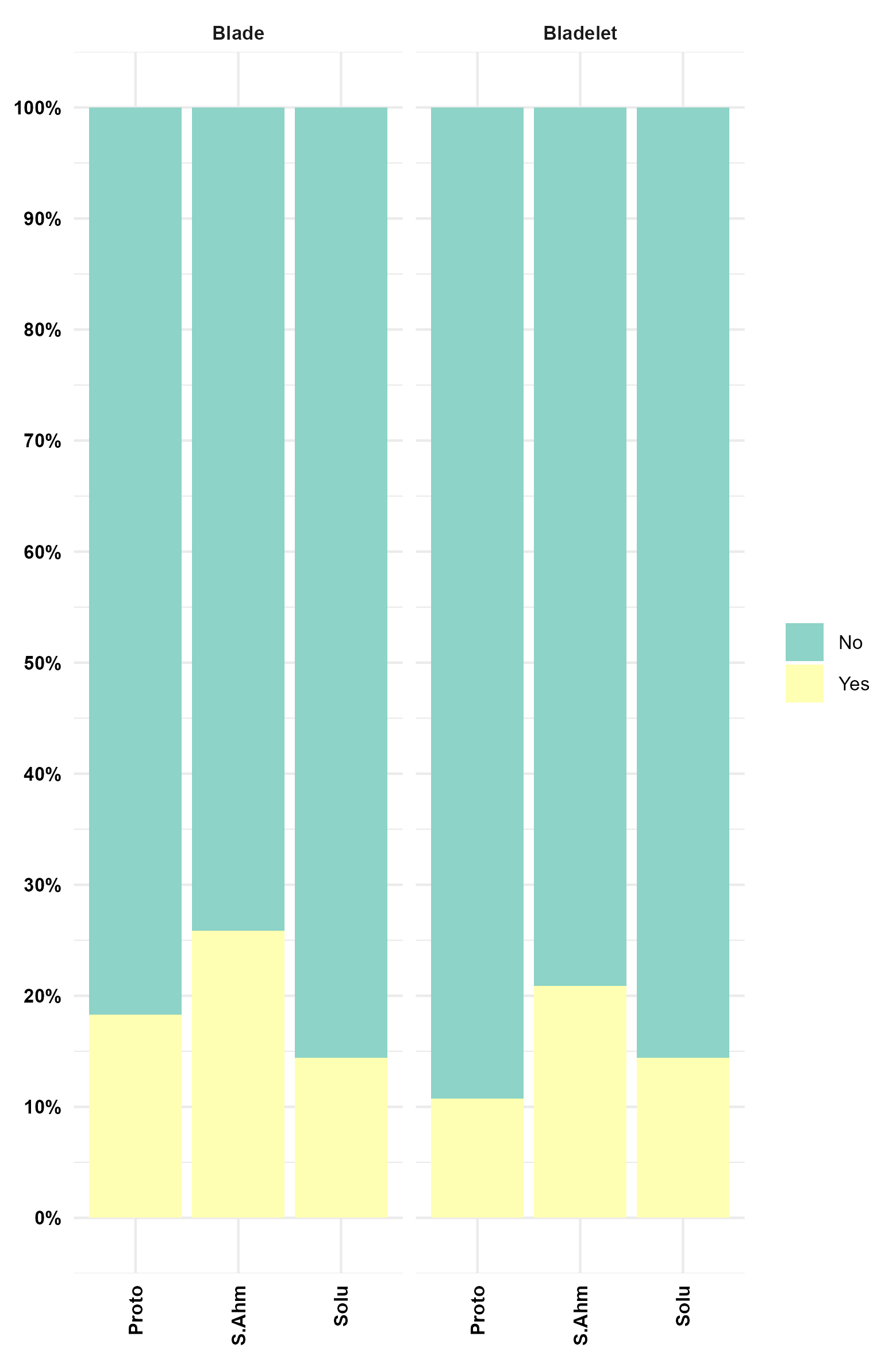

Supplement: S1 Fig — (ZIP) [file pone.0331393.s004.zip › Supporting_Information_Figures/SI_Figures_Exploratory-Plots/SIFig24_Torsion EUP-Solu.tiff]

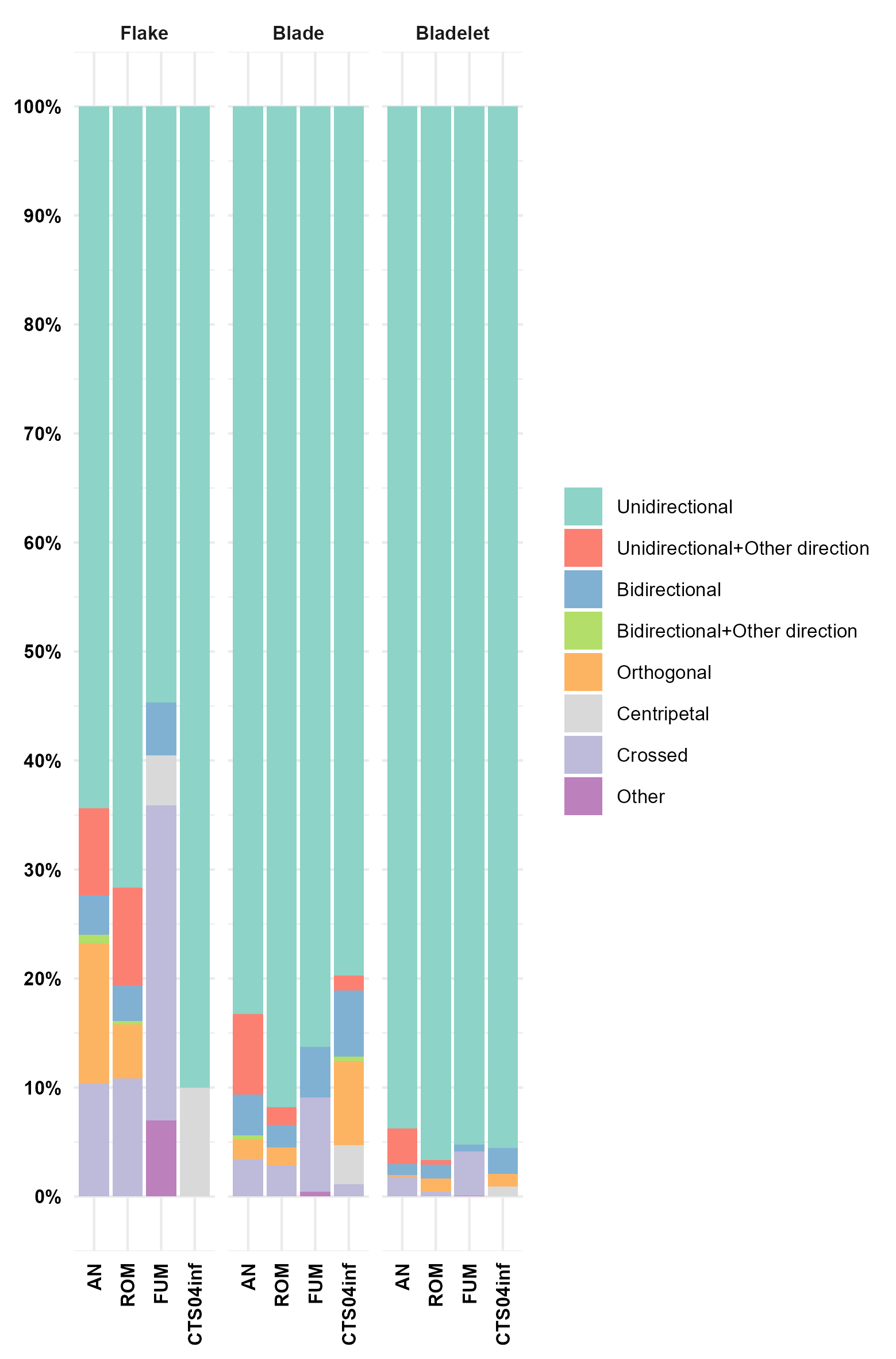

Supplement: S1 Fig — (ZIP) [file pone.0331393.s004.zip › Supporting_Information_Figures/SI_Figures_Exploratory-Plots/SIFig25_Dorsal scar.tiff]

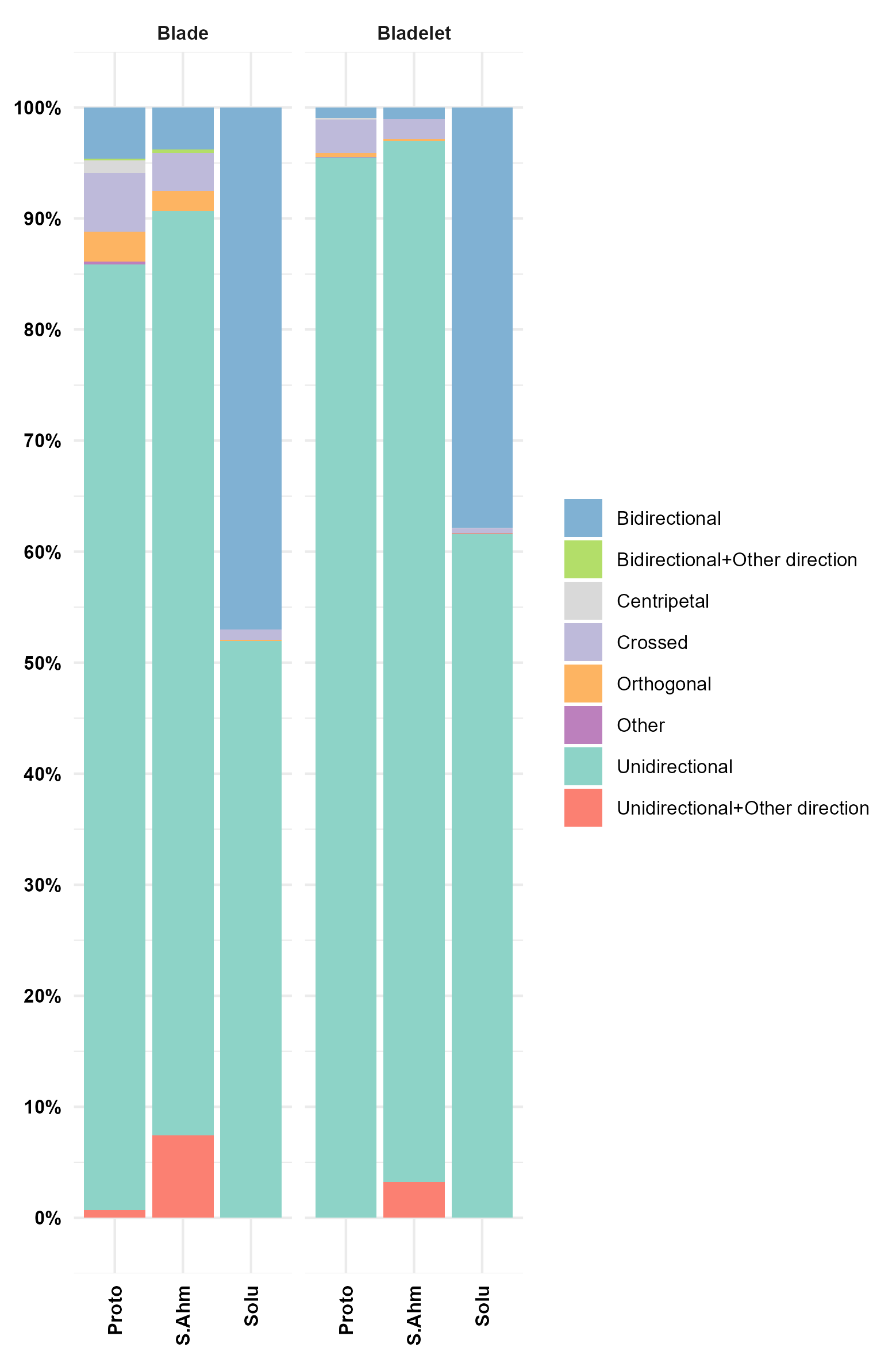

Supplement: S1 Fig — (ZIP) [file pone.0331393.s004.zip › Supporting_Information_Figures/SI_Figures_Exploratory-Plots/SIFig26_Dorsal scar EUP-Solu.tiff]

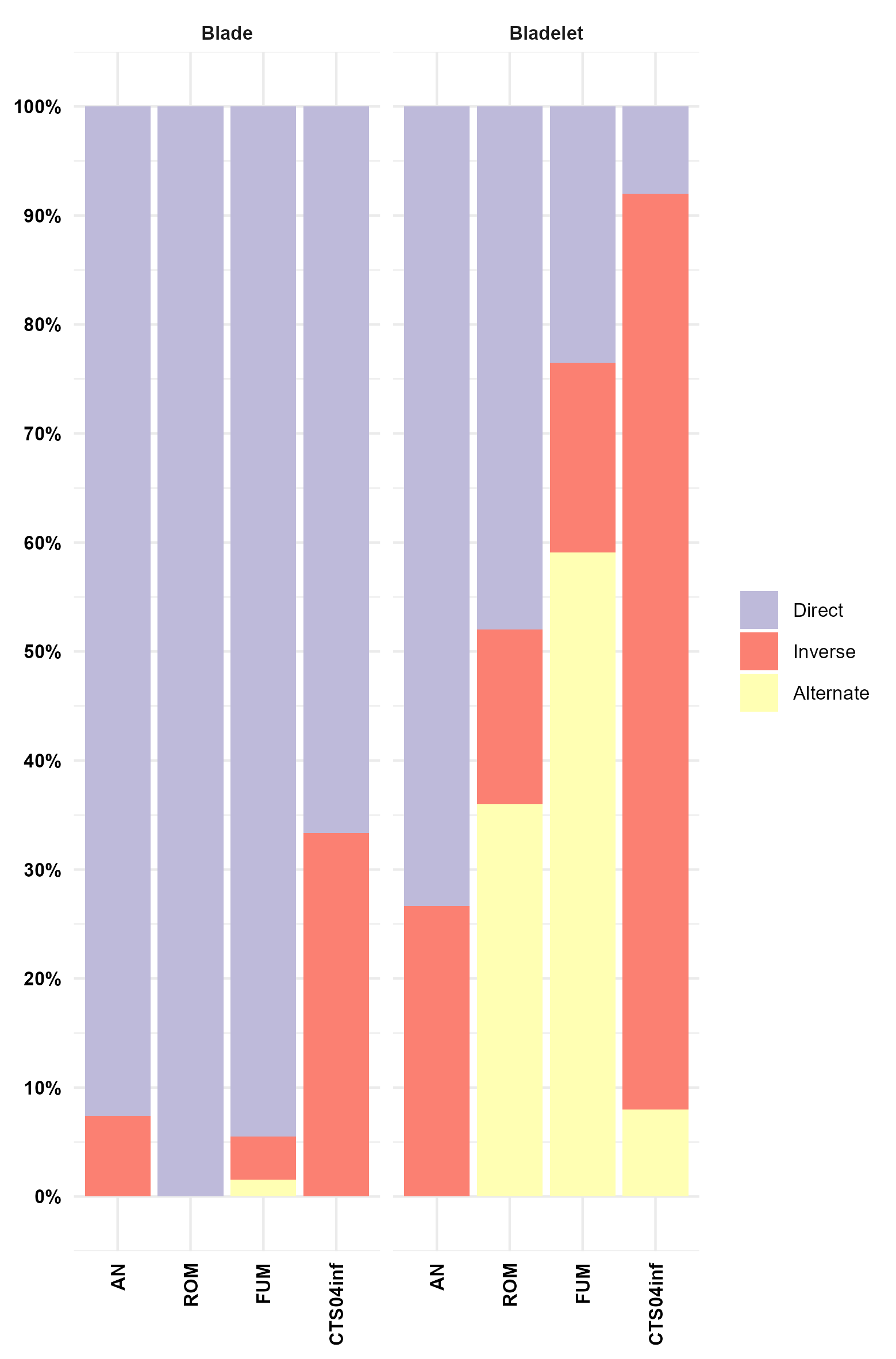

Supplement: S1 Fig — (ZIP) [file pone.0331393.s004.zip › Supporting_Information_Figures/SI_Figures_Exploratory-Plots/SIFig27_Retouch Position.tiff]

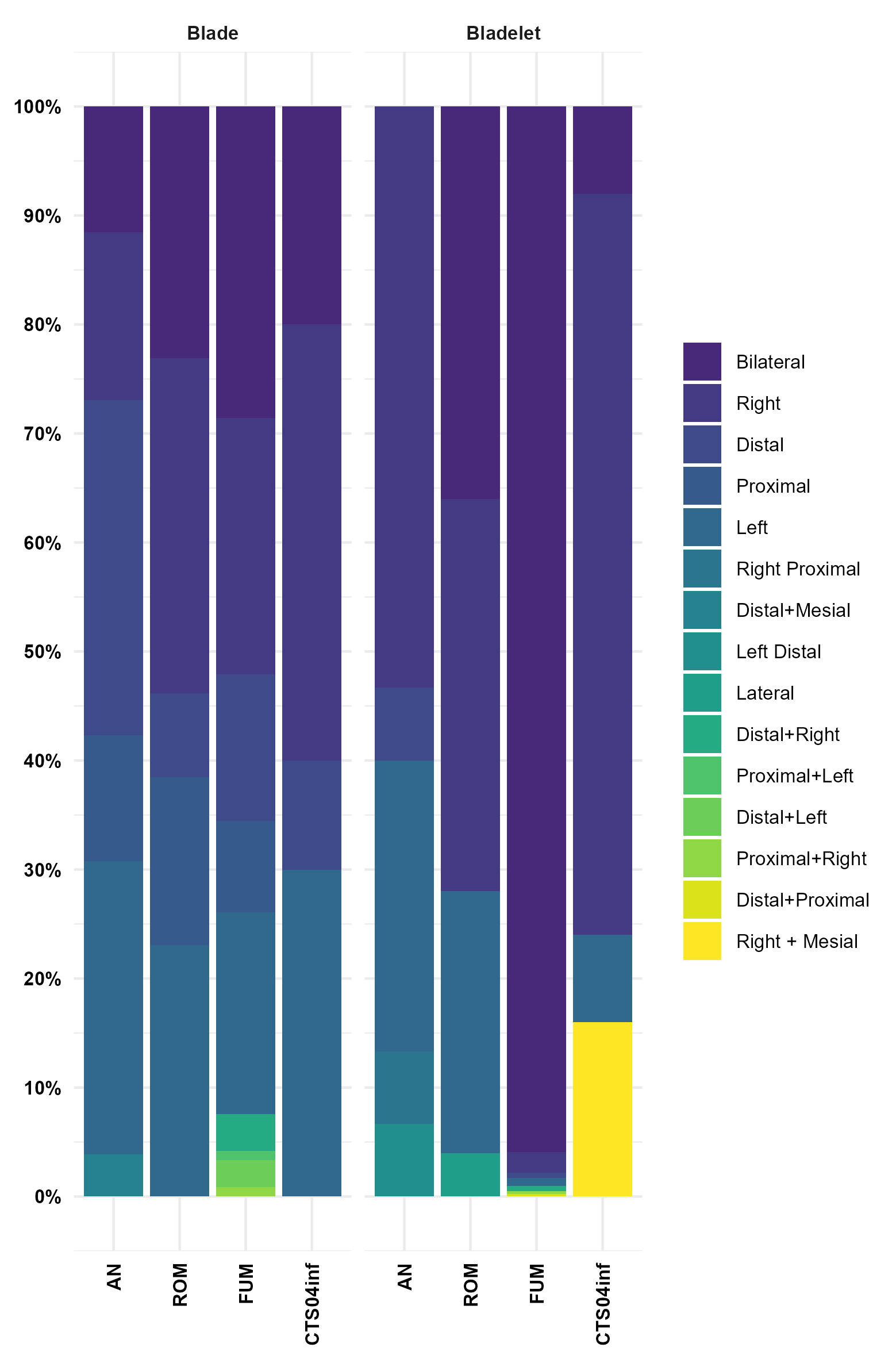

Supplement: S1 Fig — (ZIP) [file pone.0331393.s004.zip › Supporting_Information_Figures/SI_Figures_Exploratory-Plots/SIFig28_Retouch Location.tiff]

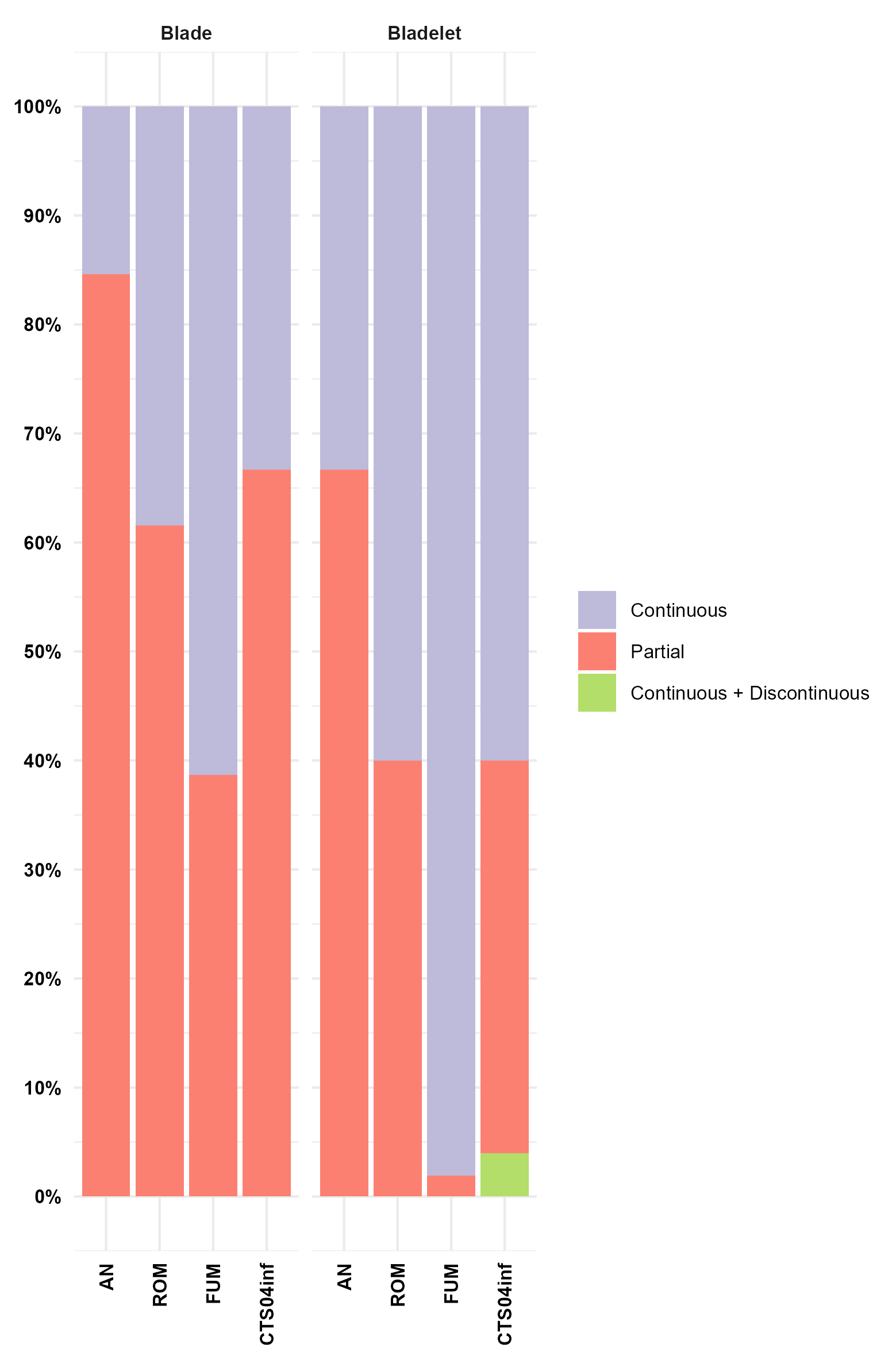

Supplement: S1 Fig — (ZIP) [file pone.0331393.s004.zip › Supporting_Information_Figures/SI_Figures_Exploratory-Plots/SIFig29_Retouch Distribution.tiff]

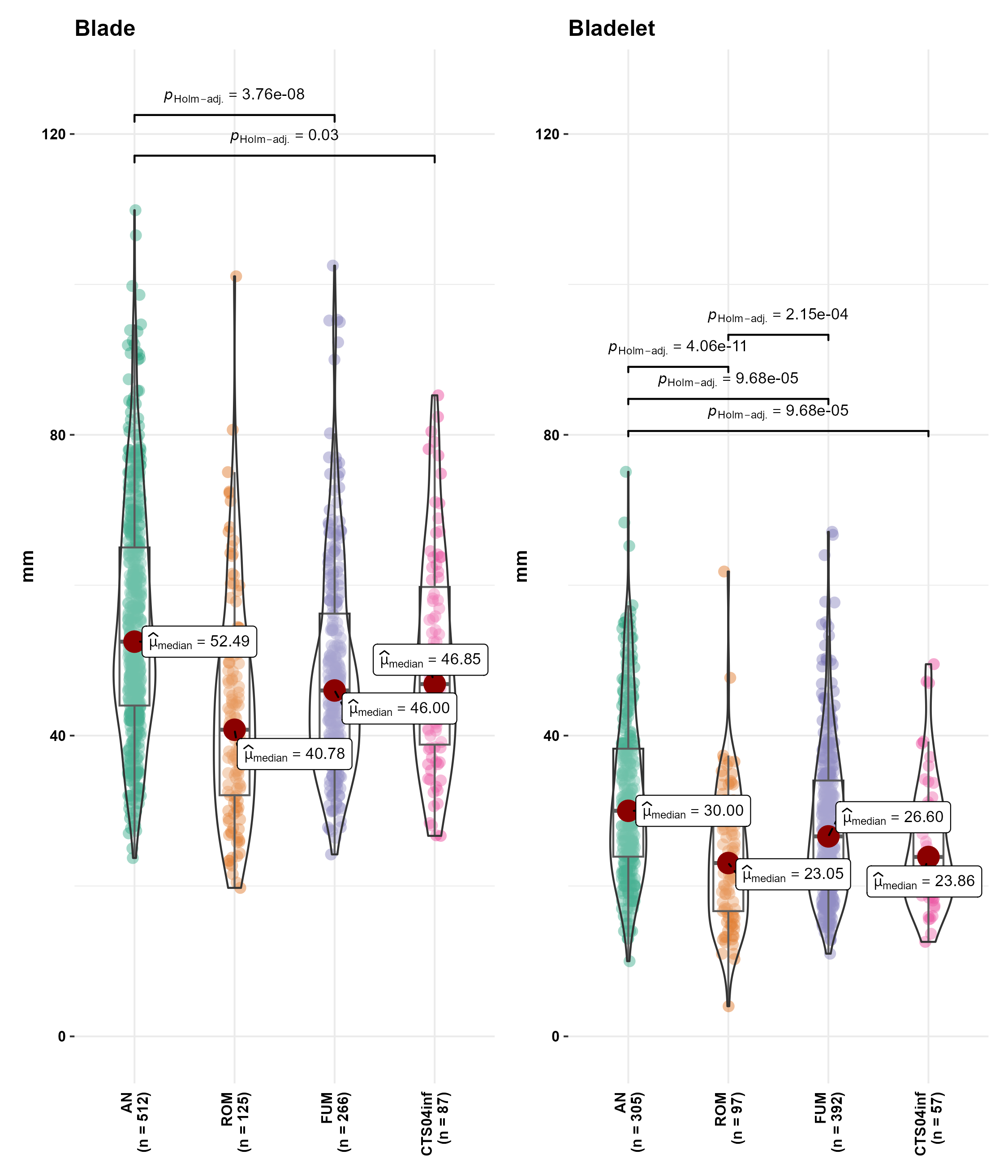

Supplement: S1 Fig — (ZIP) [file pone.0331393.s004.zip › Supporting_Information_Figures/SI_Figures_Exploratory-Plots/SIFig30_Length.tiff]

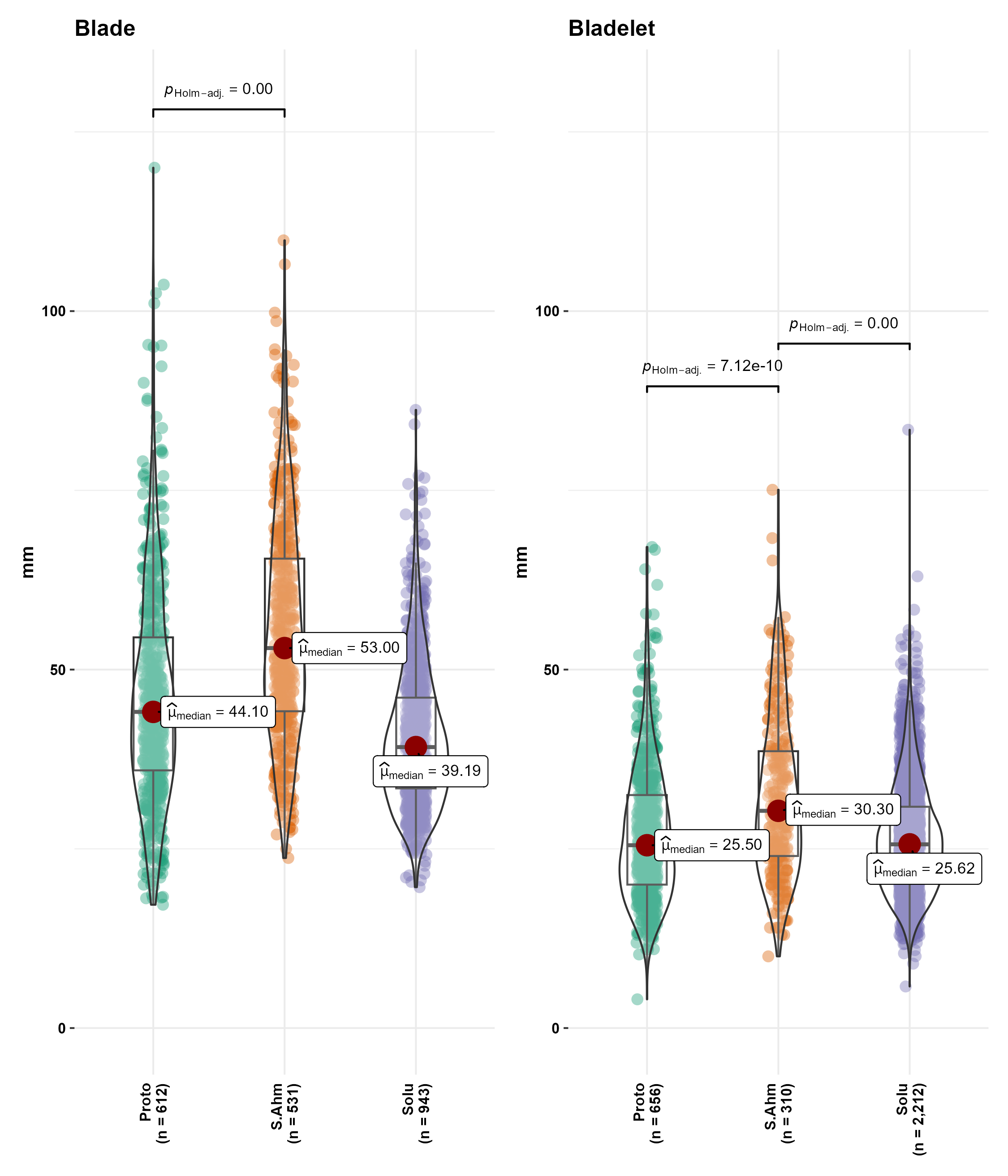

Supplement: S1 Fig — (ZIP) [file pone.0331393.s004.zip › Supporting_Information_Figures/SI_Figures_Exploratory-Plots/SIFig31_Length EUP-Solu.tiff]

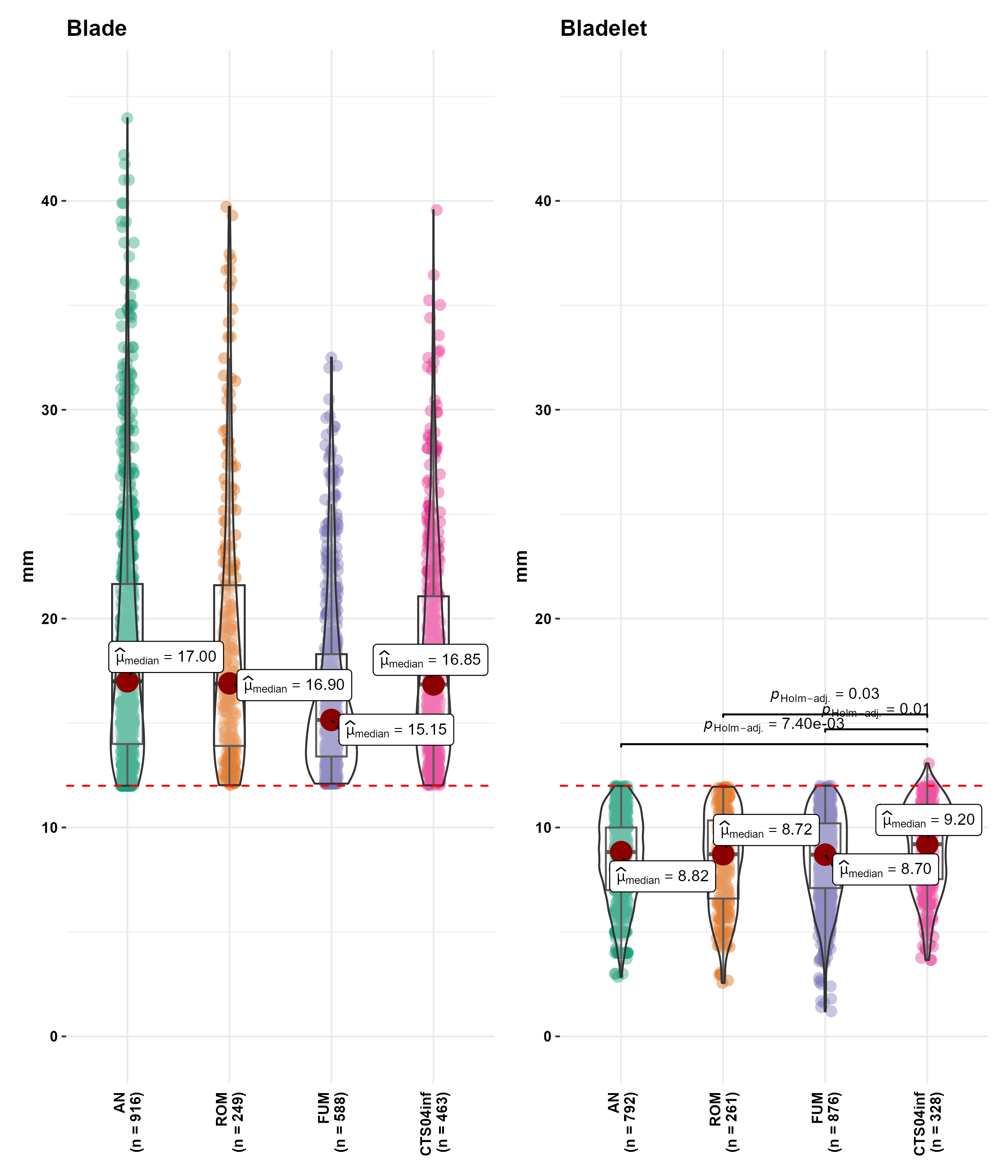

Supplement: S1 Fig — (ZIP) [file pone.0331393.s004.zip › Supporting_Information_Figures/SI_Figures_Exploratory-Plots/SIFig32_Width.tiff]

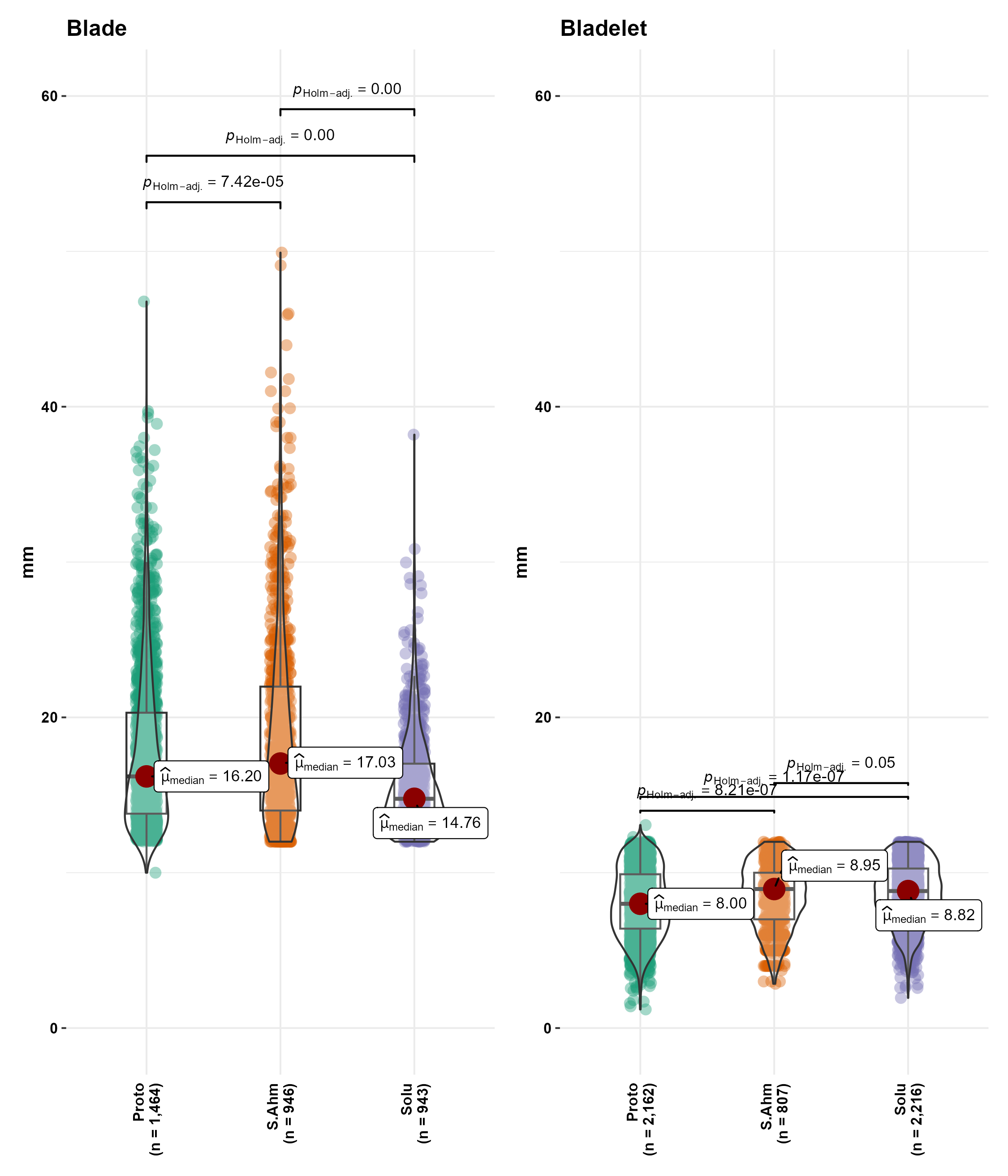

Supplement: S1 Fig — (ZIP) [file pone.0331393.s004.zip › Supporting_Information_Figures/SI_Figures_Exploratory-Plots/SIFig33_Width EUP-Solu.tiff]

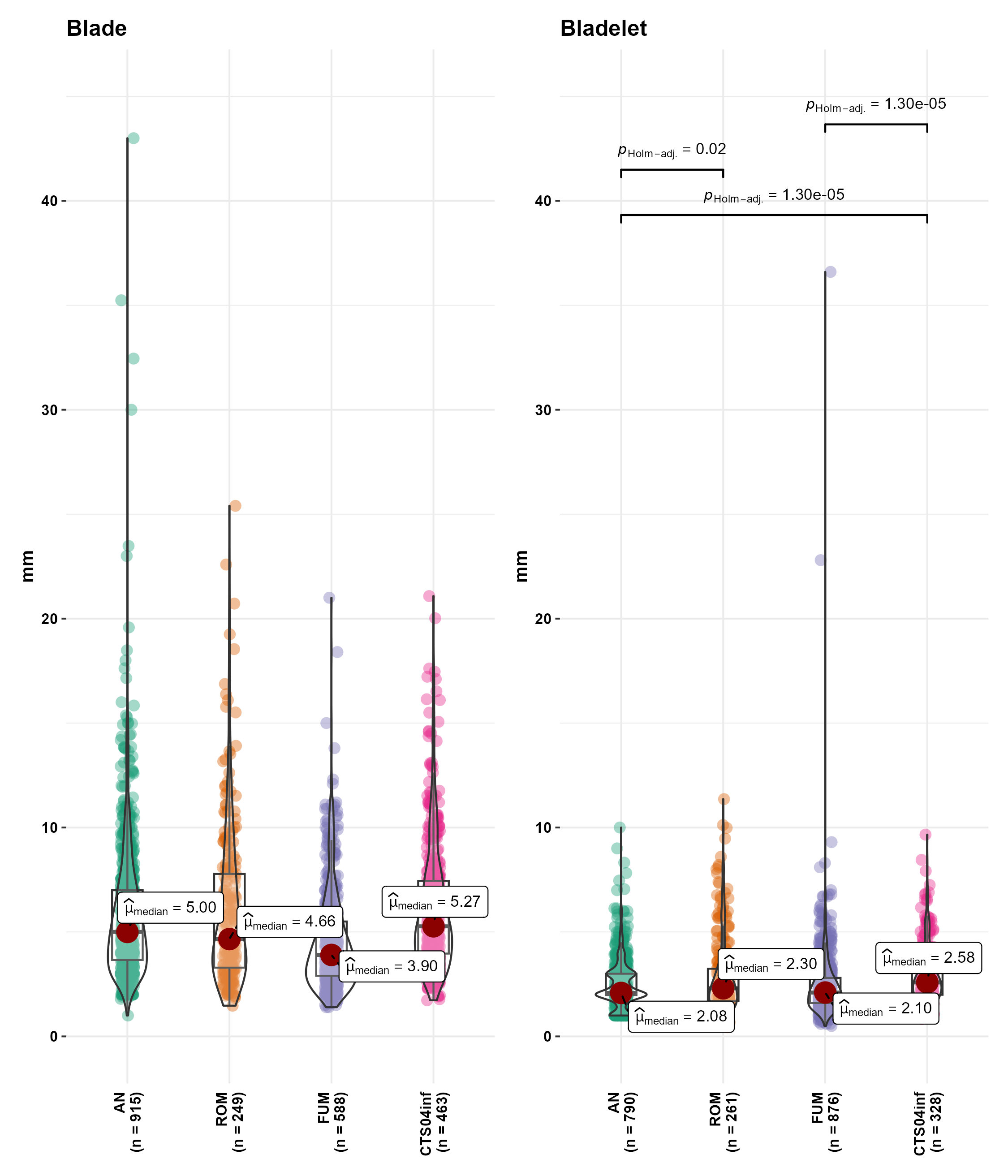

Supplement: S1 Fig — (ZIP) [file pone.0331393.s004.zip › Supporting_Information_Figures/SI_Figures_Exploratory-Plots/SIFig34_Thickness.tiff]

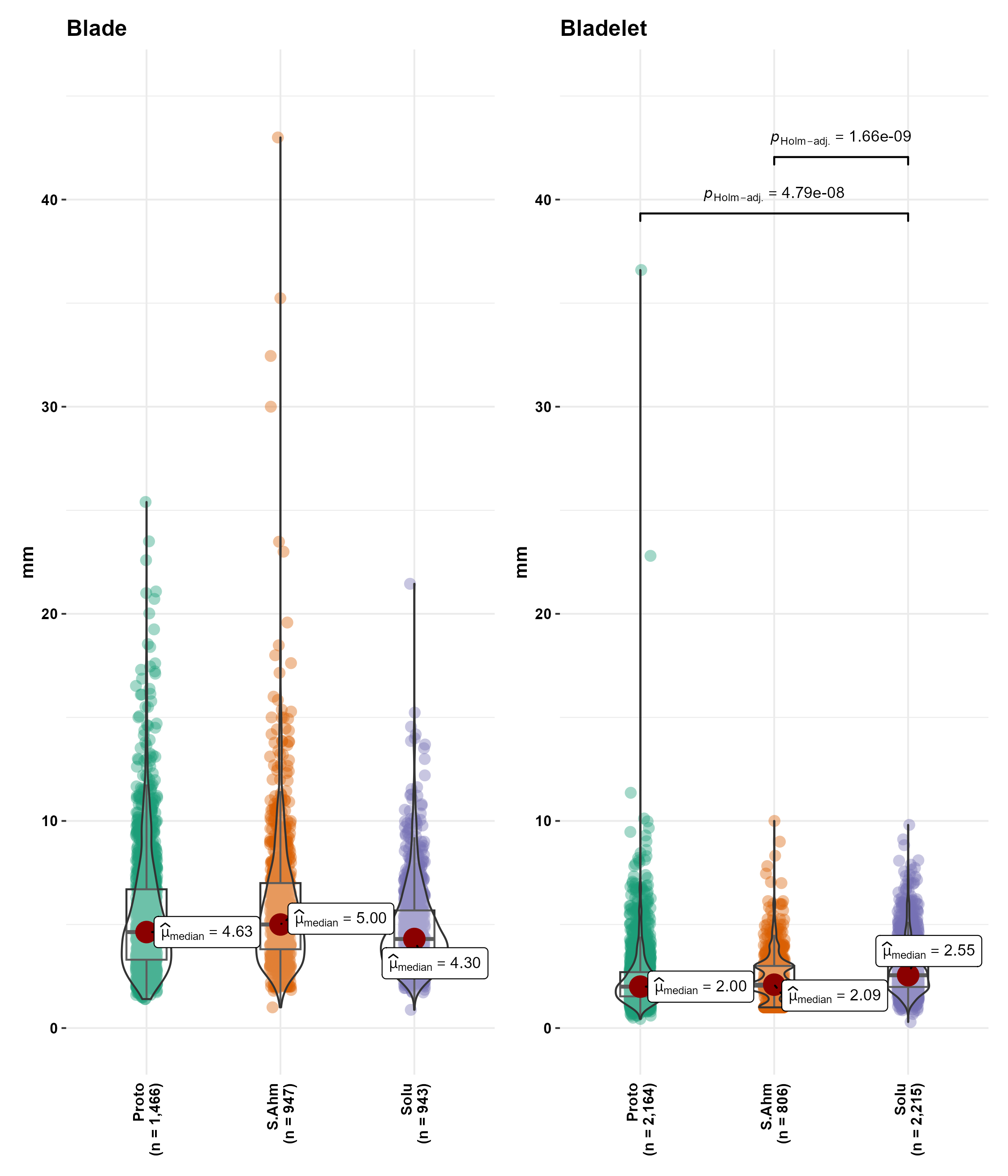

Supplement: S1 Fig — (ZIP) [file pone.0331393.s004.zip › Supporting_Information_Figures/SI_Figures_Exploratory-Plots/SIFig35_Thickness EUP-Solu.tiff]

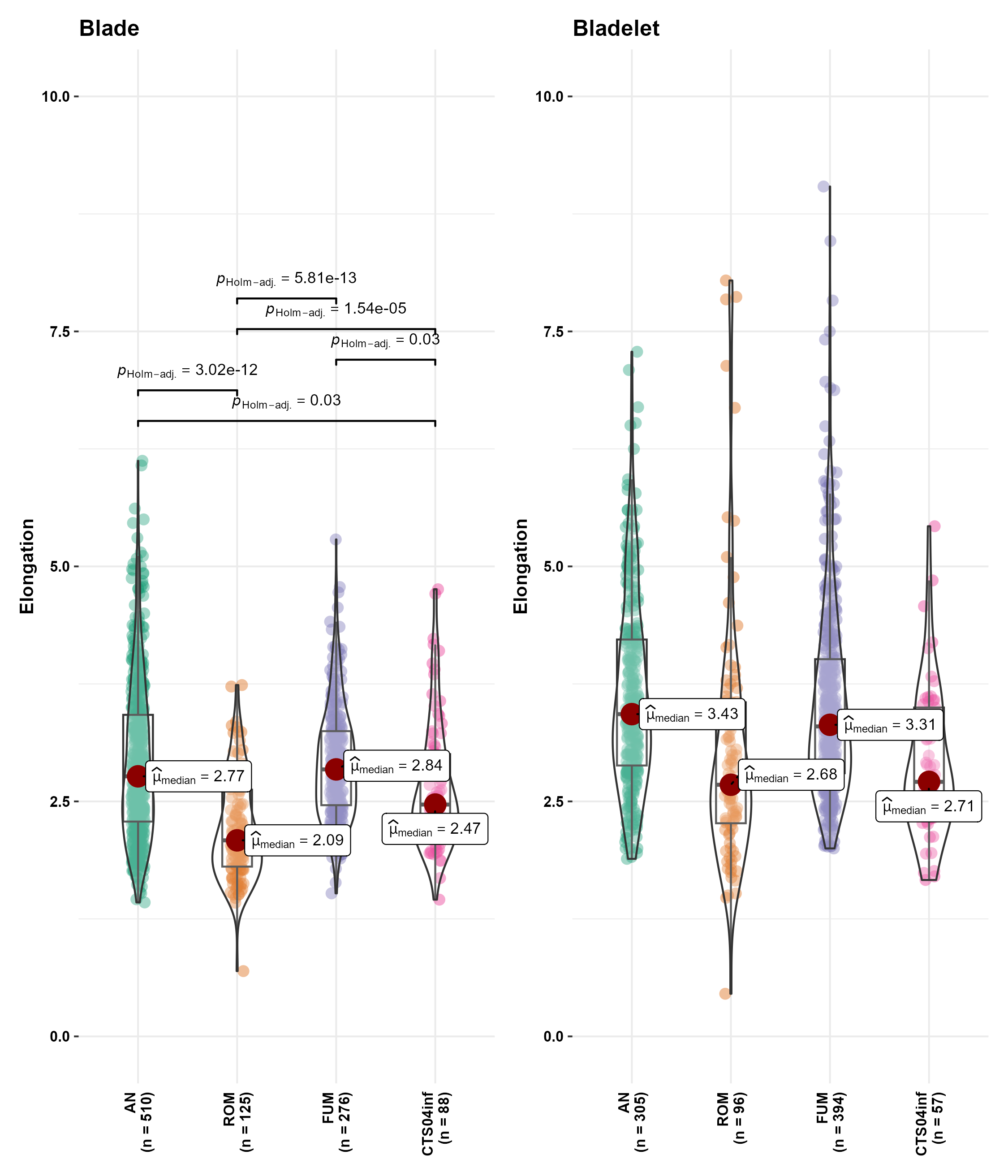

Supplement: S1 Fig — (ZIP) [file pone.0331393.s004.zip › Supporting_Information_Figures/SI_Figures_Exploratory-Plots/SIFig36_Elongation.tiff]

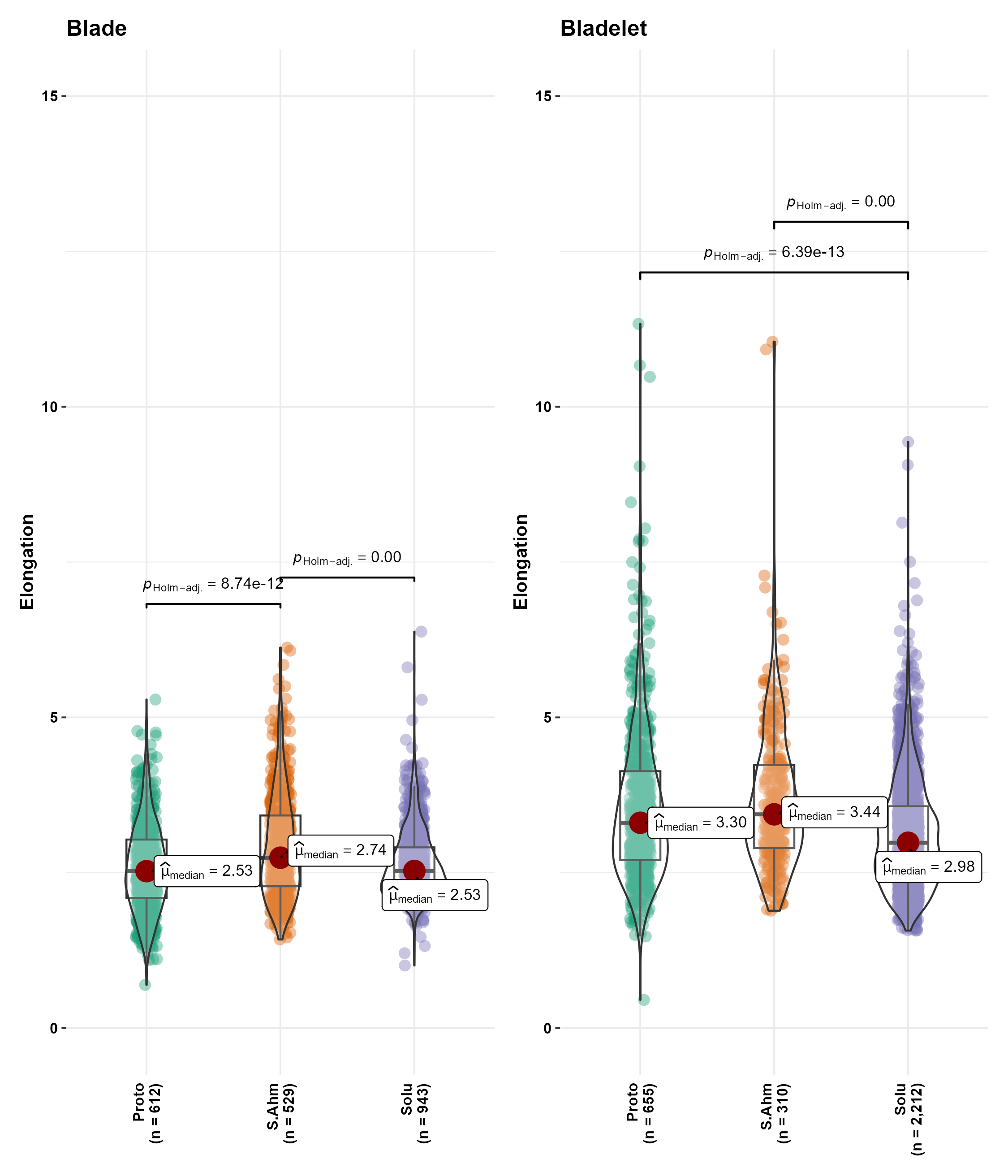

Supplement: S1 Fig — (ZIP) [file pone.0331393.s004.zip › Supporting_Information_Figures/SI_Figures_Exploratory-Plots/SIFig37_Elongation EUP-Solu.tiff]

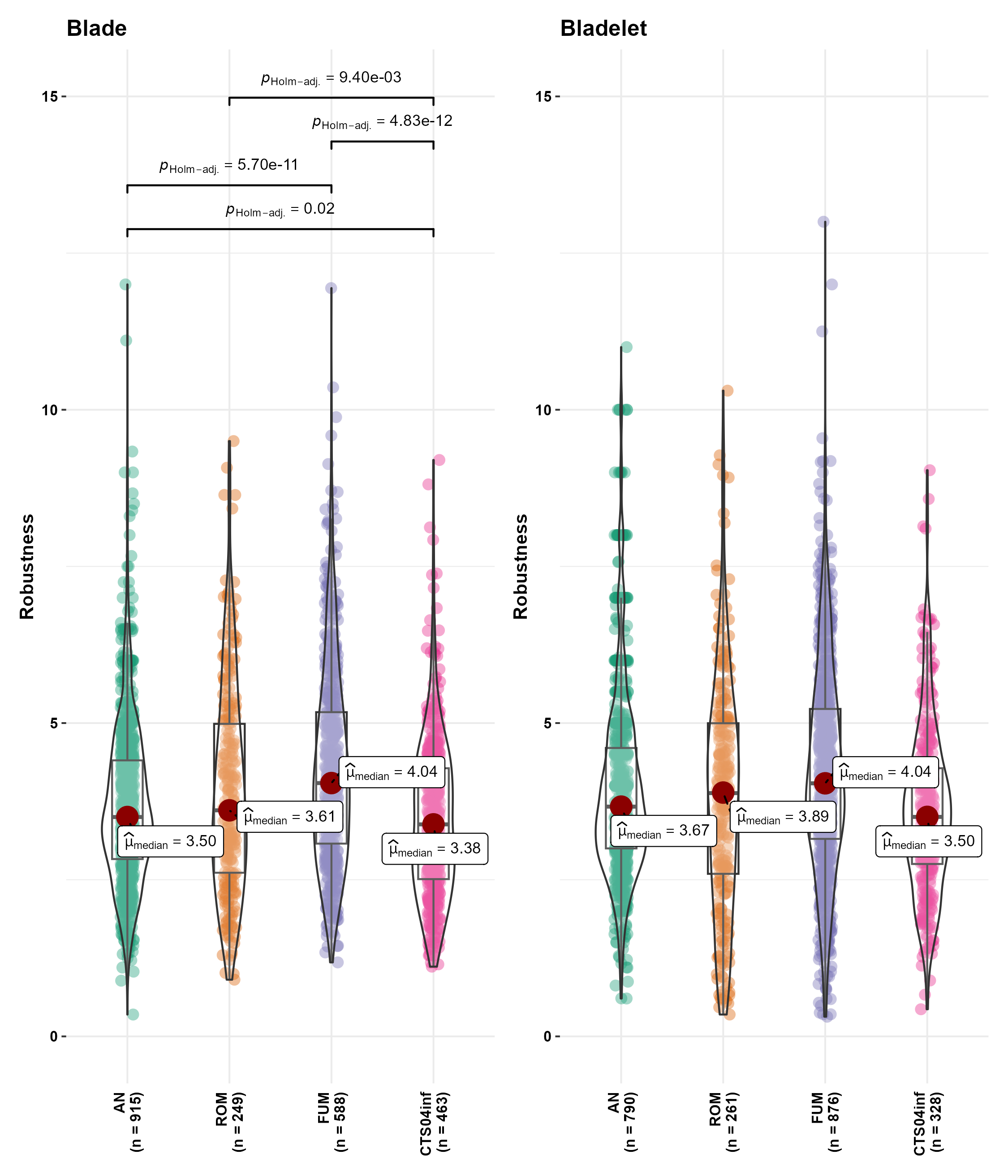

Supplement: S1 Fig — (ZIP) [file pone.0331393.s004.zip › Supporting_Information_Figures/SI_Figures_Exploratory-Plots/SIFig38_Robustness.tiff]

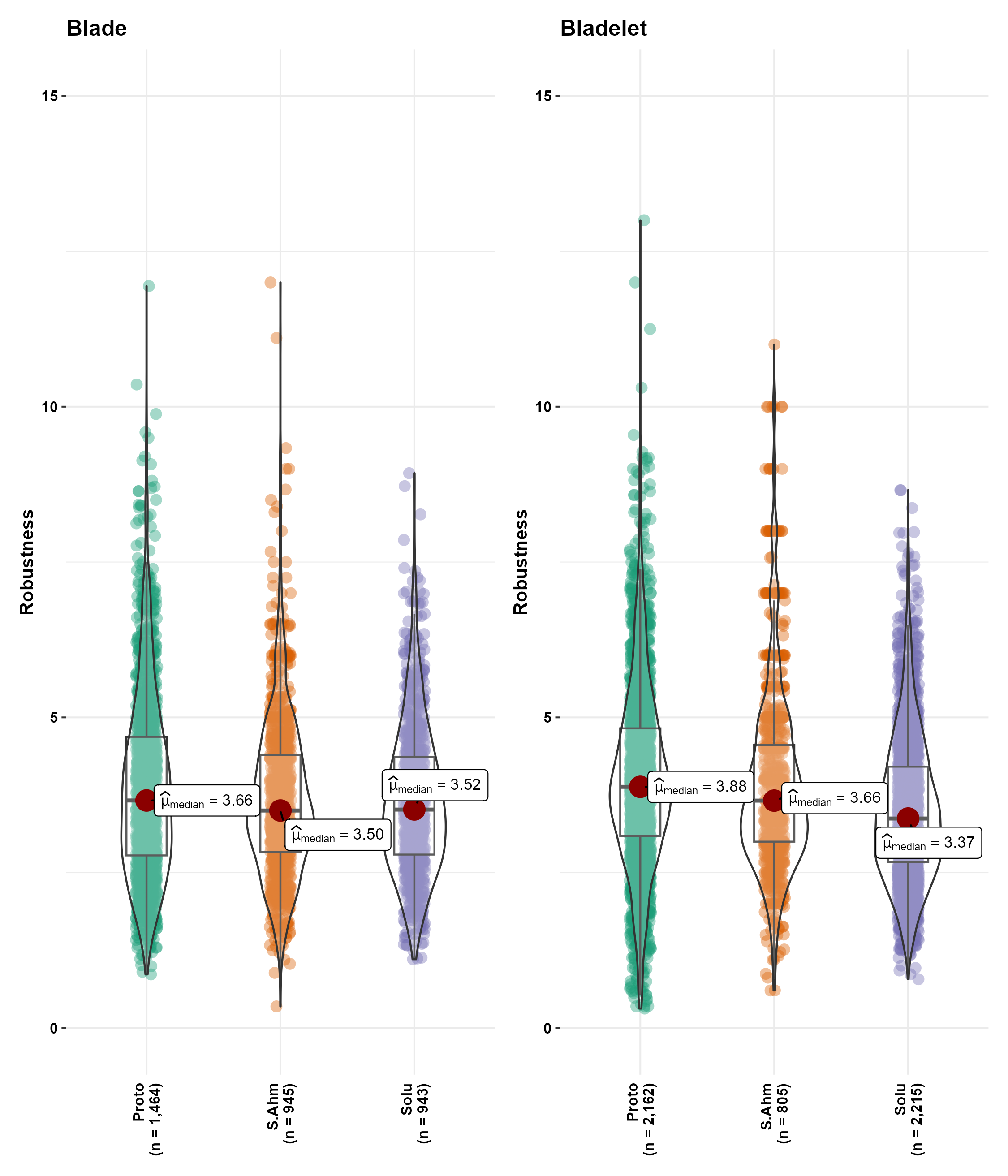

Supplement: S1 Fig — (ZIP) [file pone.0331393.s004.zip › Supporting_Information_Figures/SI_Figures_Exploratory-Plots/SIFig39_Robustness EUP-Solu.tiff]

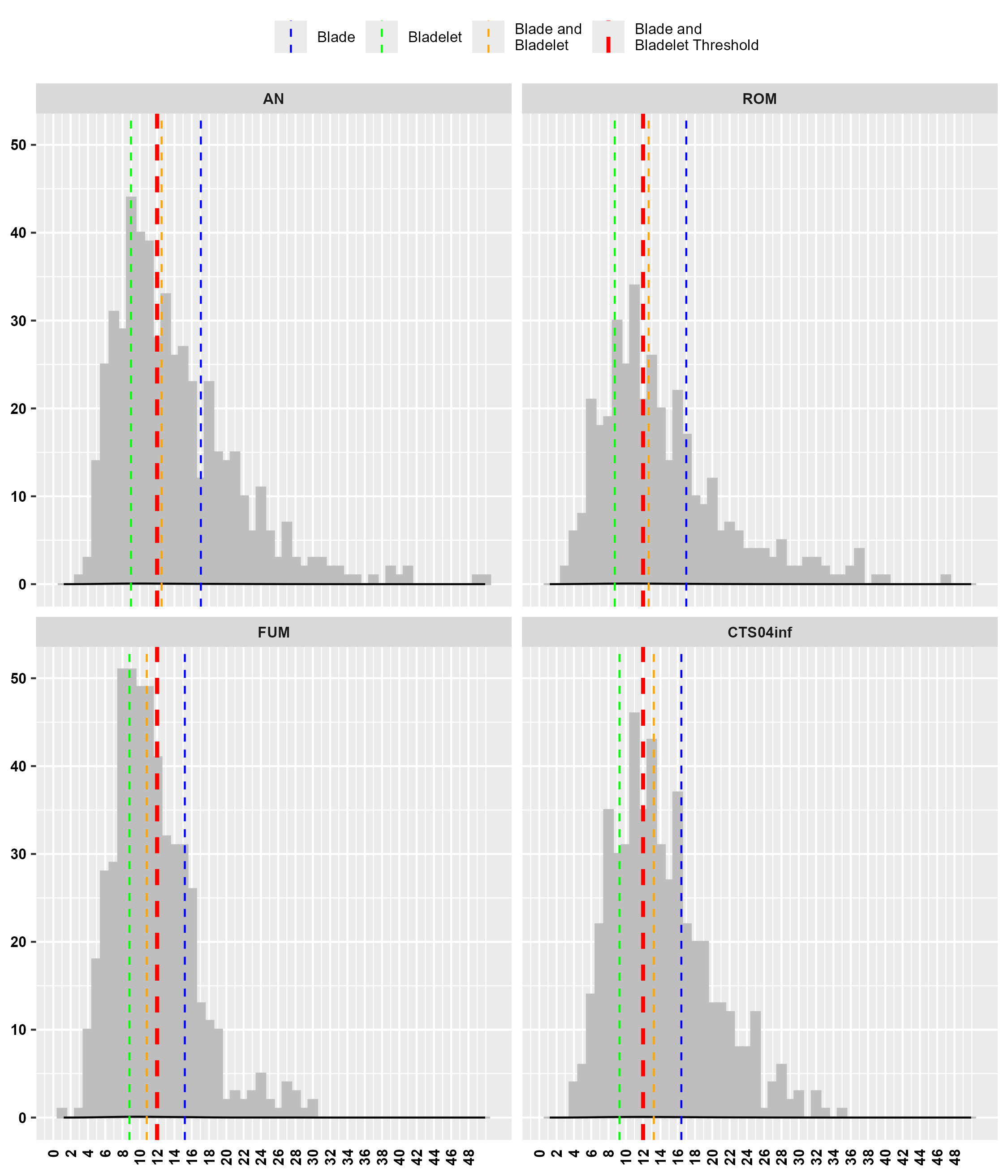

Supplement: S1 Fig — (ZIP) [file pone.0331393.s004.zip › Supporting_Information_Figures/SI_Figures_Exploratory-Plots/SIFig40_Width histogram MNF.tiff]

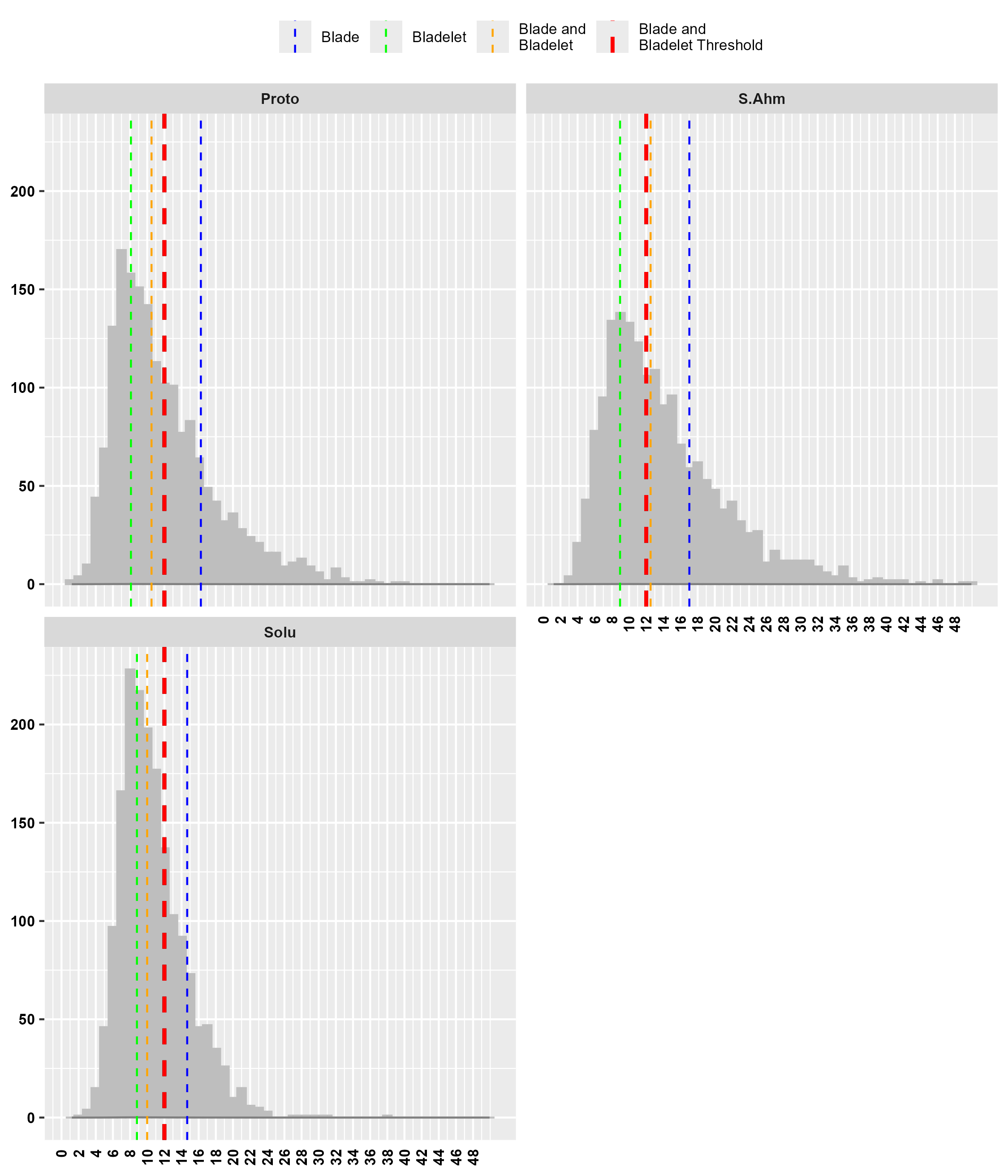

Supplement: S1 Fig — (ZIP) [file pone.0331393.s004.zip › Supporting_Information_Figures/SI_Figures_Exploratory-Plots/SIFig41_Width histogram EUP-Solu.tiff]

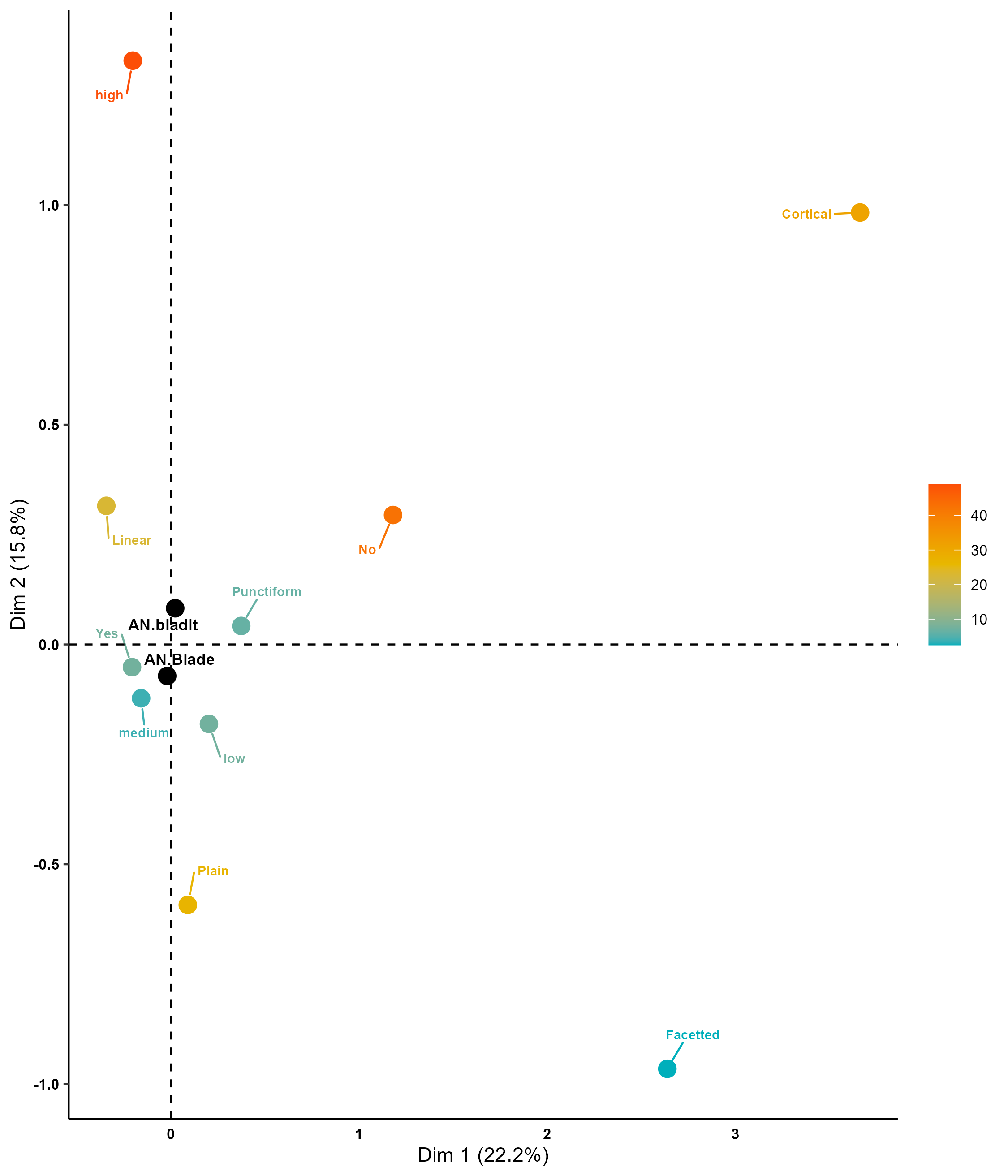

Supplement: S1 Fig — (ZIP) [file pone.0331393.s004.zip › Supporting_Information_Figures/SI_Figures_MCA-Biplots_Correlation-Plots/SIFig42_MCAPlatformAN.tiff]

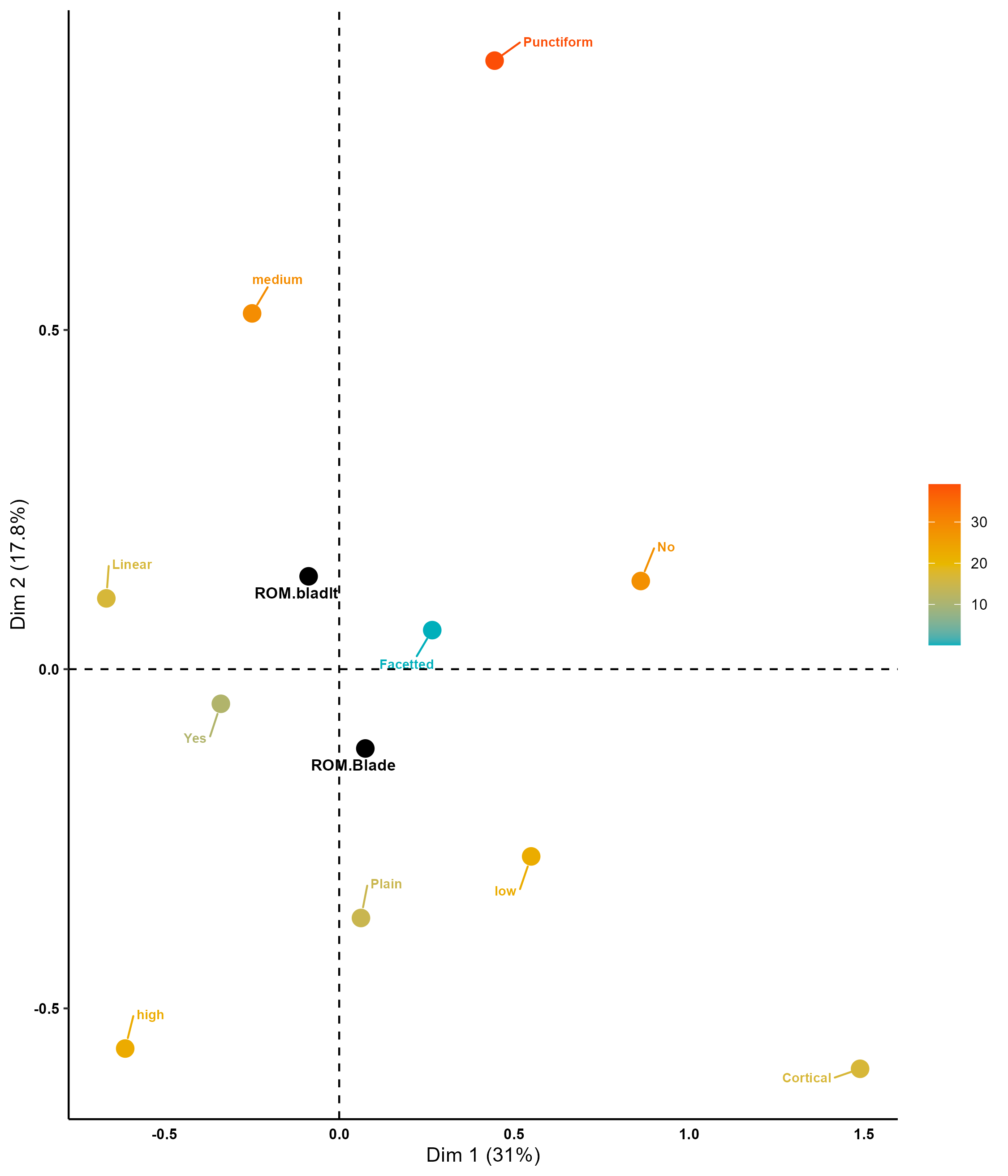

Supplement: S1 Fig — (ZIP) [file pone.0331393.s004.zip › Supporting_Information_Figures/SI_Figures_MCA-Biplots_Correlation-Plots/SIFig43_MCAPlatformROM.tiff]

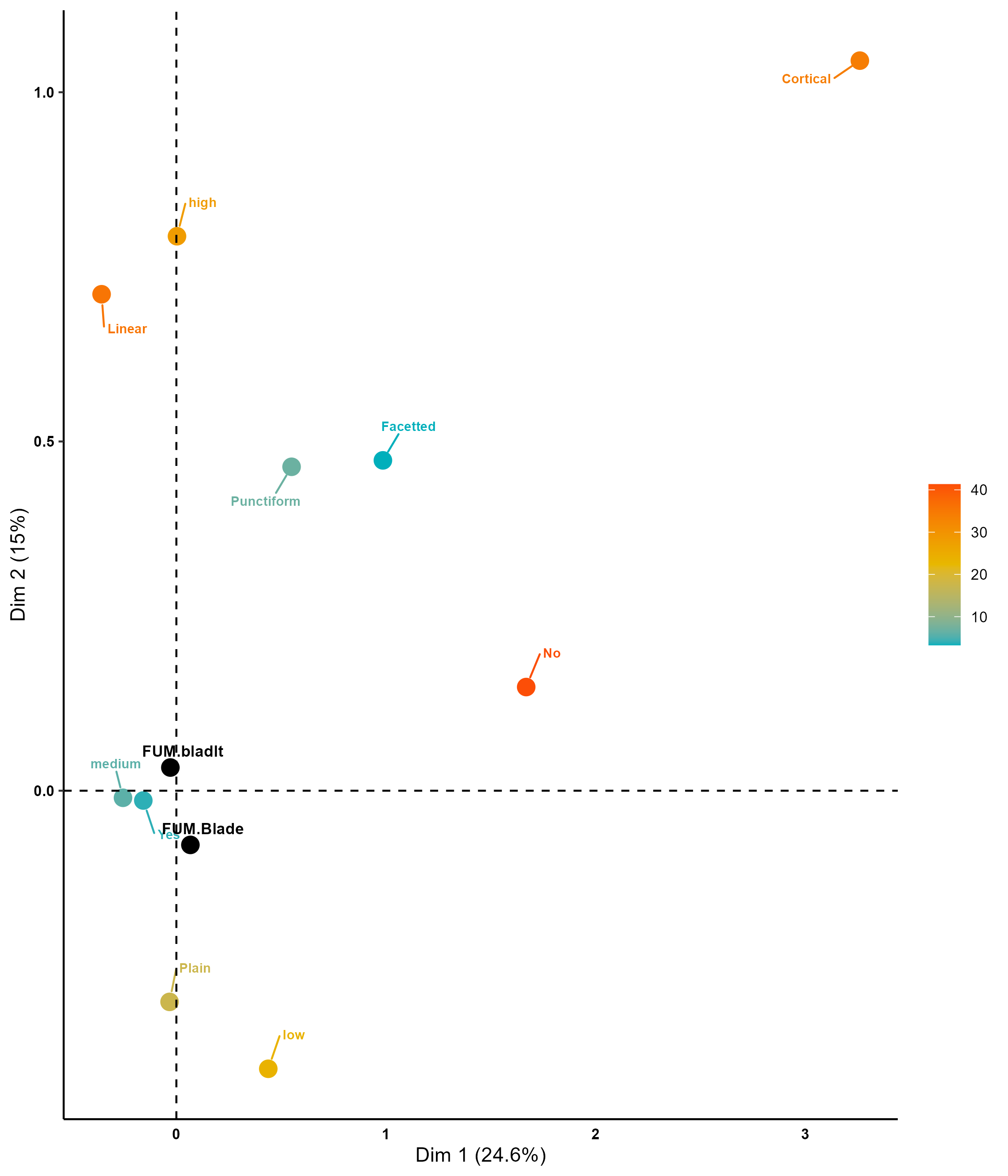

Supplement: S1 Fig — (ZIP) [file pone.0331393.s004.zip › Supporting_Information_Figures/SI_Figures_MCA-Biplots_Correlation-Plots/SIFig44_MCAPlatformFUM.tiff]

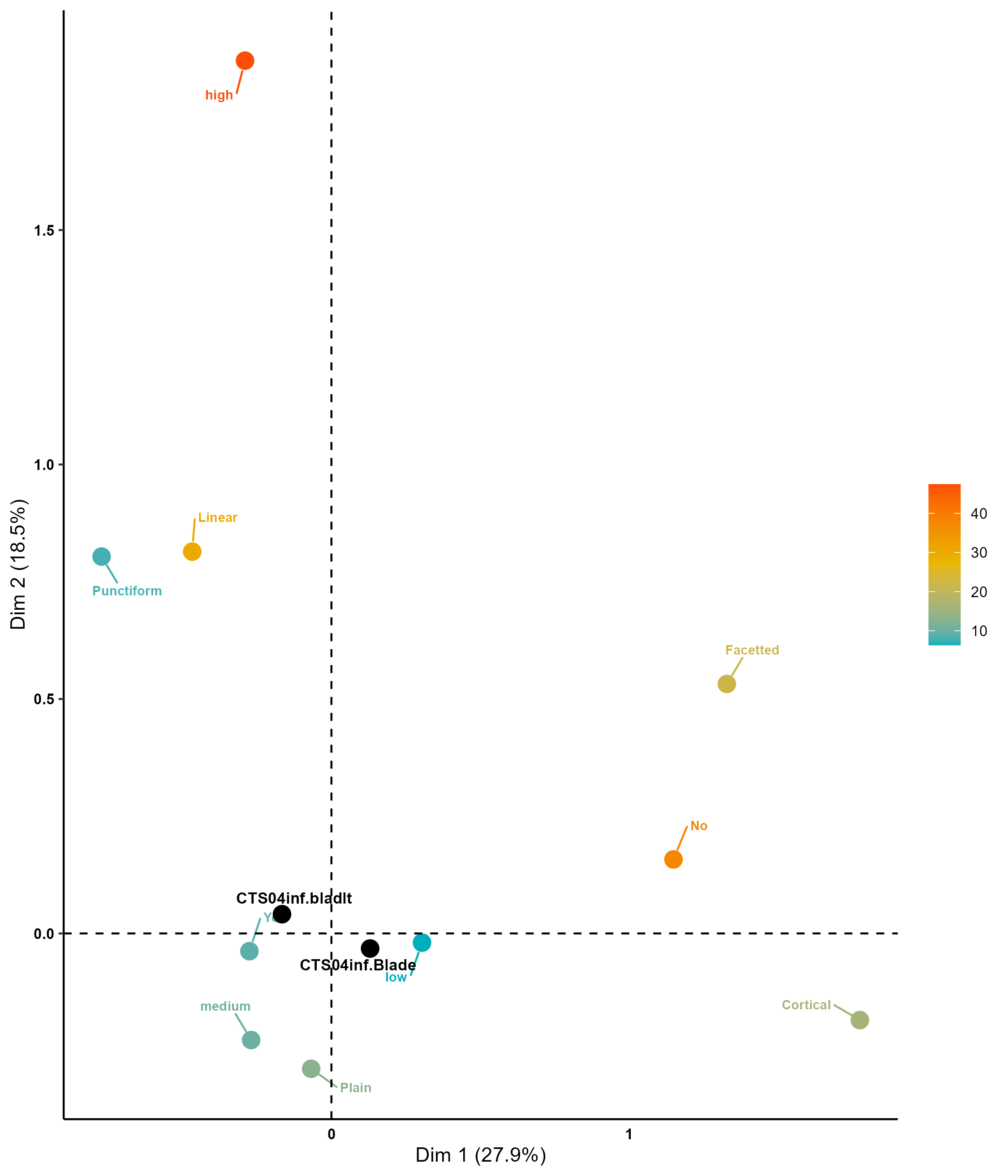

Supplement: S1 Fig — (ZIP) [file pone.0331393.s004.zip › Supporting_Information_Figures/SI_Figures_MCA-Biplots_Correlation-Plots/SIFig45_MCPlatformCTS.tiff]

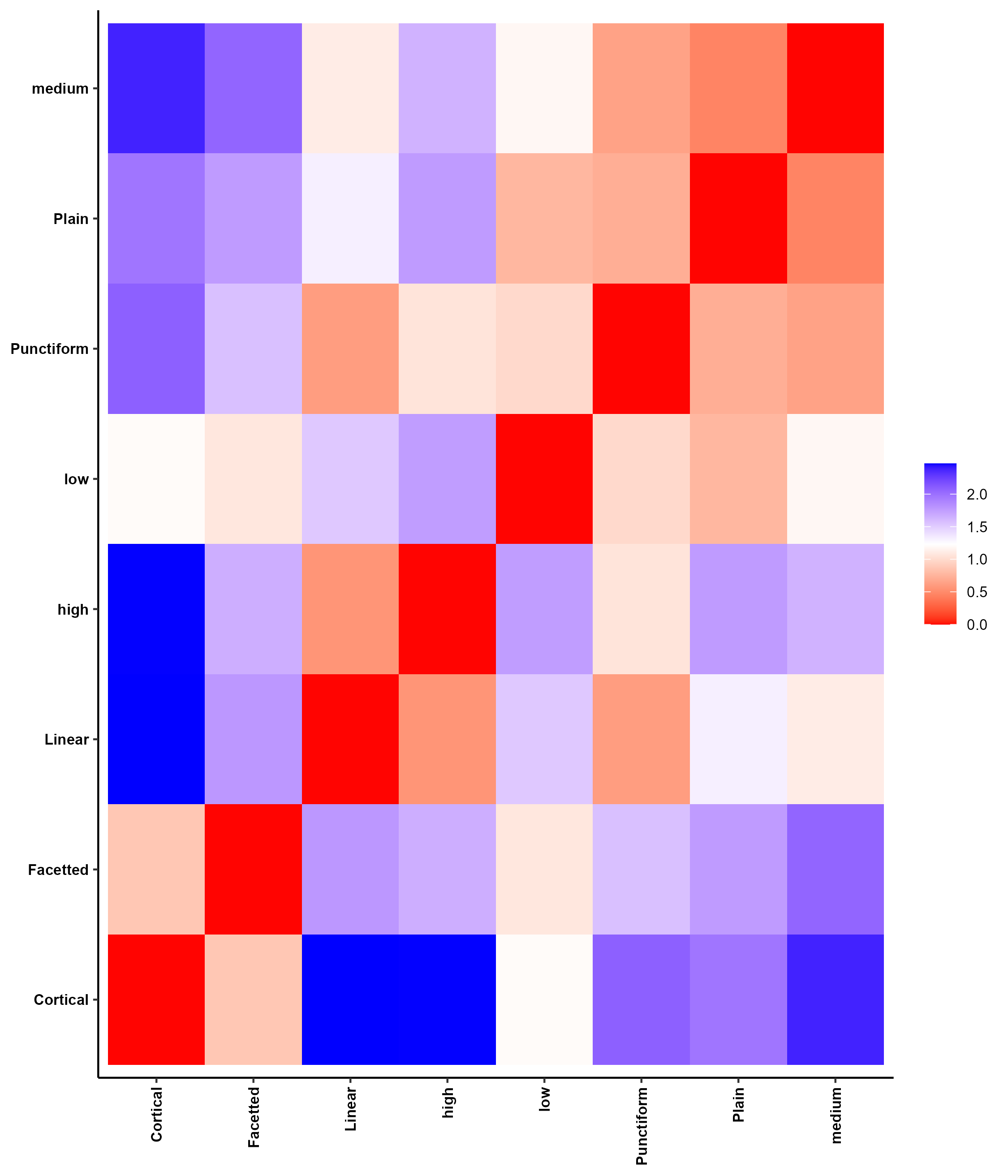

Supplement: S1 Fig — (ZIP) [file pone.0331393.s004.zip › Supporting_Information_Figures/SI_Figures_MCA-Biplots_Correlation-Plots/SIFig46_CORRPlatformCONTROL.tiff]

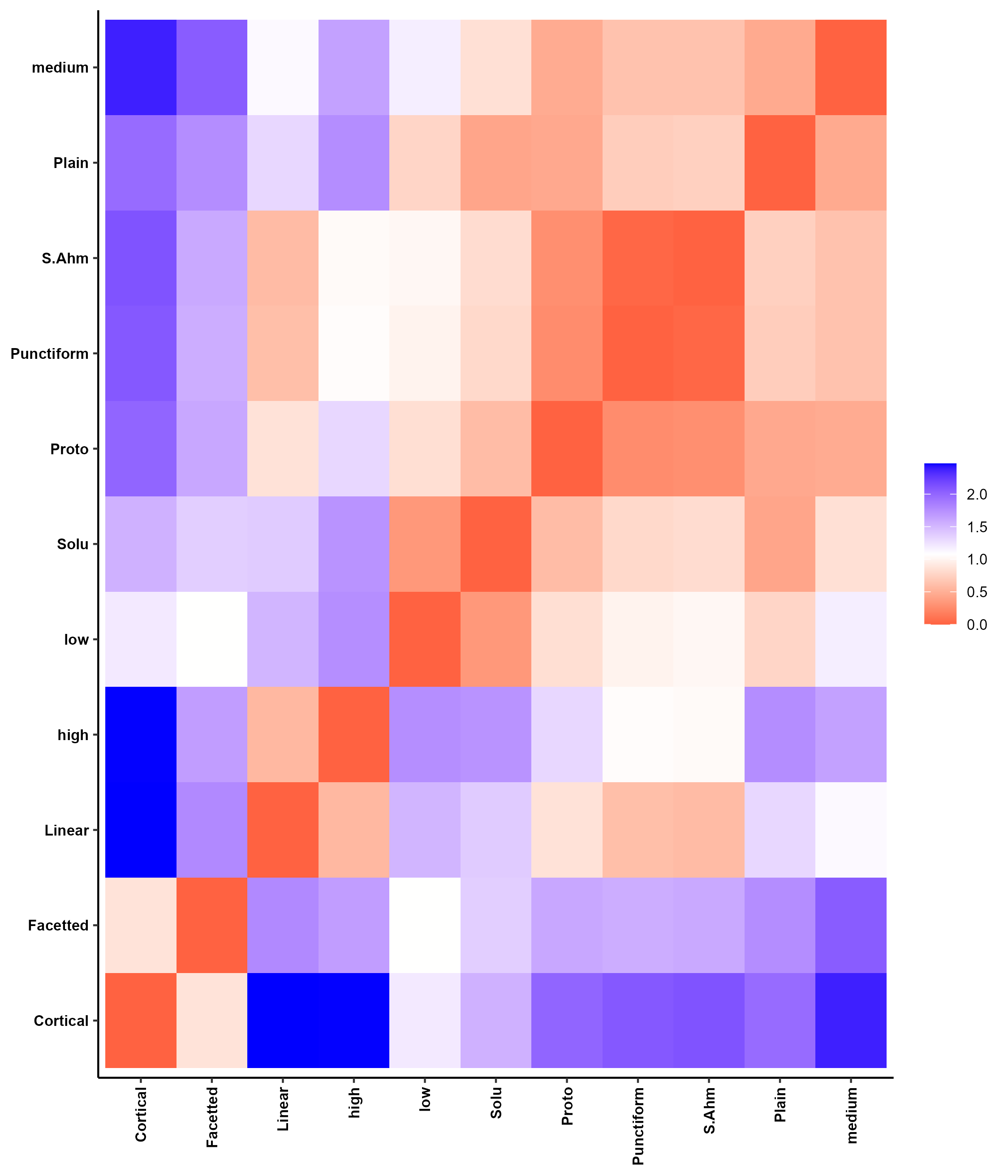

Supplement: S1 Fig — (ZIP) [file pone.0331393.s004.zip › Supporting_Information_Figures/SI_Figures_MCA-Biplots_Correlation-Plots/SIFig47_CORRPlatformCONTROL_withquali.tiff]

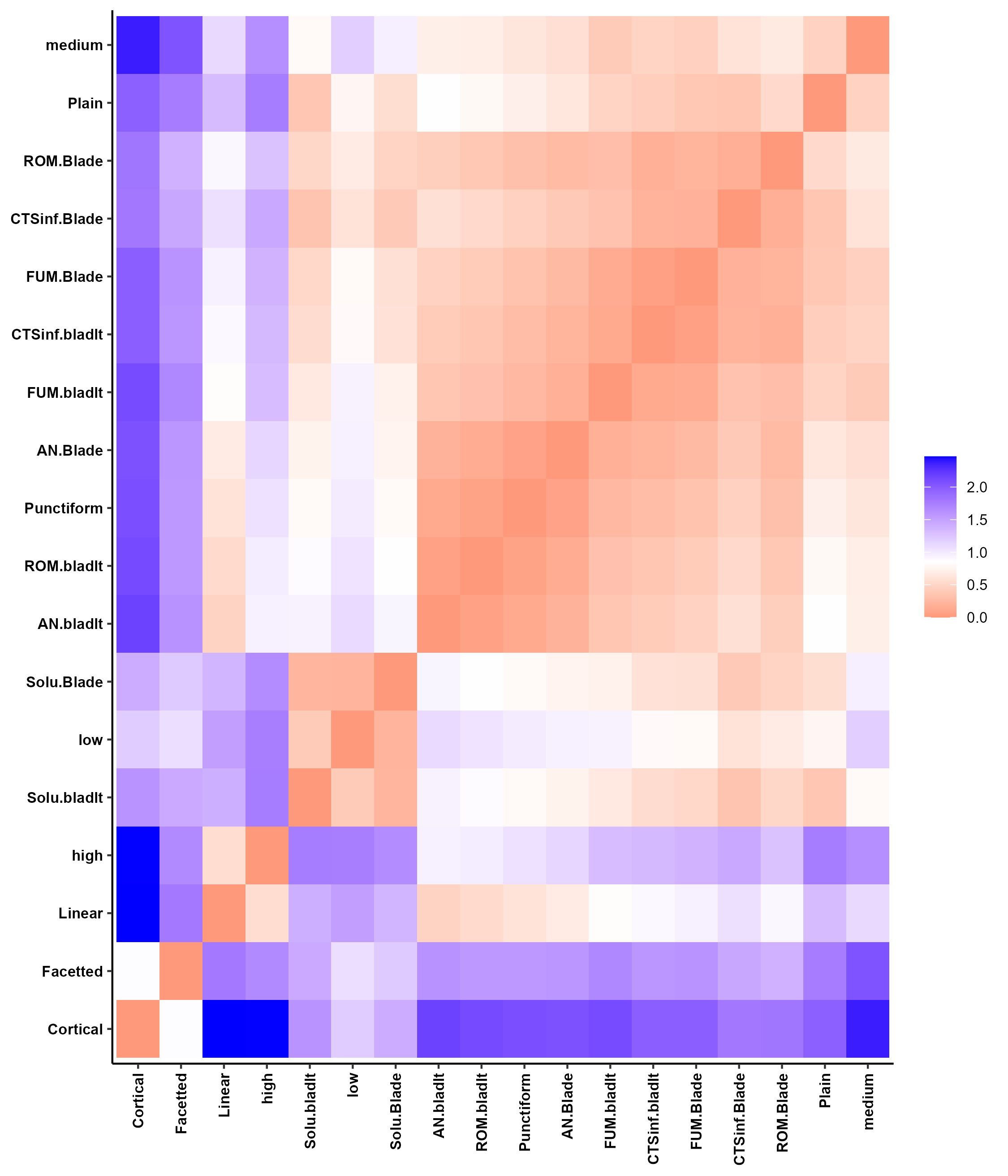

Supplement: S1 Fig — (ZIP) [file pone.0331393.s004.zip › Supporting_Information_Figures/SI_Figures_MCA-Biplots_Correlation-Plots/SIFig48_CORRPlatformCONTROL_withquali_2.tiff]

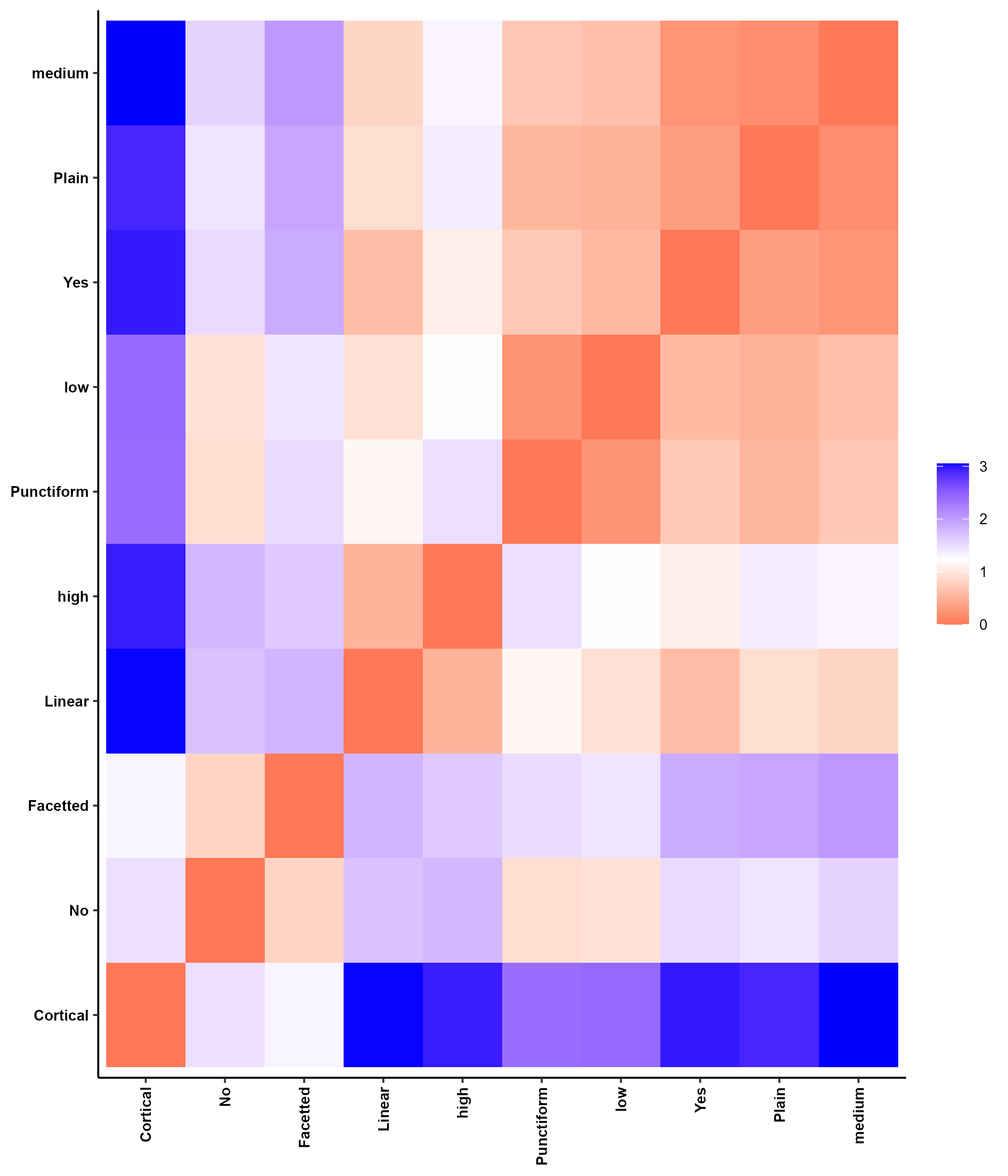

Supplement: S1 Fig — (ZIP) [file pone.0331393.s004.zip › Supporting_Information_Figures/SI_Figures_MCA-Biplots_Correlation-Plots/SIFig49_CORRPlatformALL.tiff]

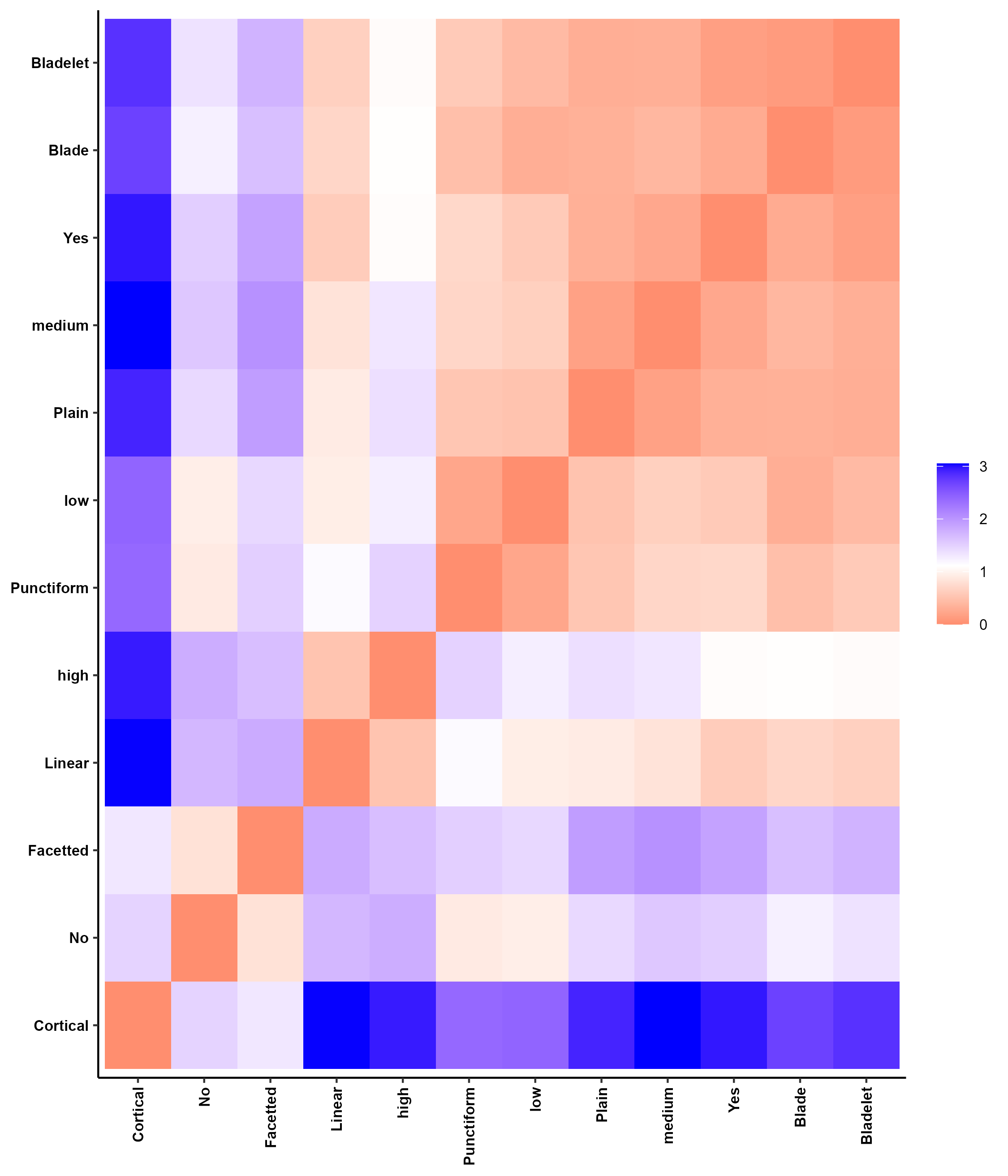

Supplement: S1 Fig — (ZIP) [file pone.0331393.s004.zip › Supporting_Information_Figures/SI_Figures_MCA-Biplots_Correlation-Plots/SIFig50_CORRPlatformALL_withquali.tiff]

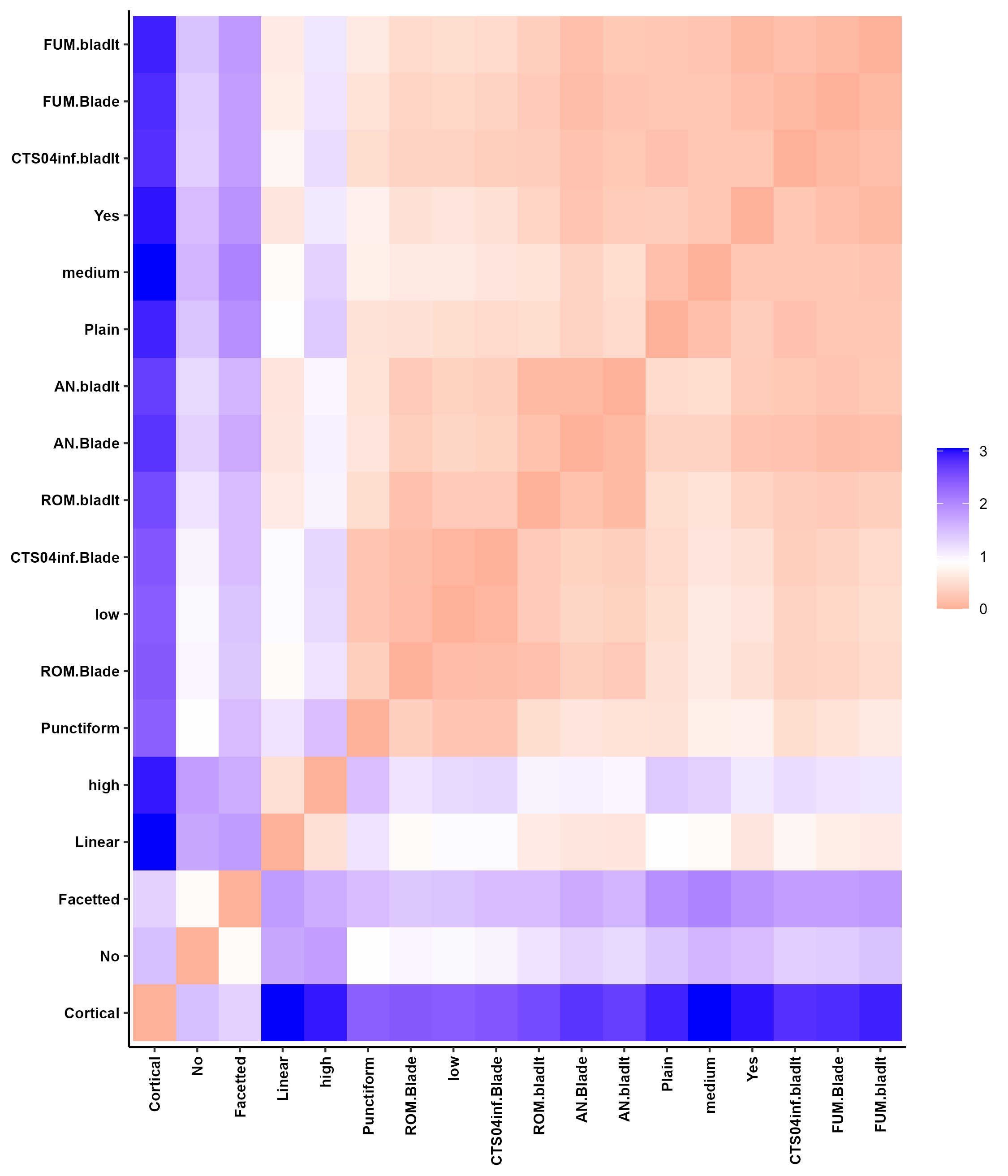

Supplement: S1 Fig — (ZIP) [file pone.0331393.s004.zip › Supporting_Information_Figures/SI_Figures_MCA-Biplots_Correlation-Plots/SIFig51_CORRPlatformALL_withquali_2.tiff]

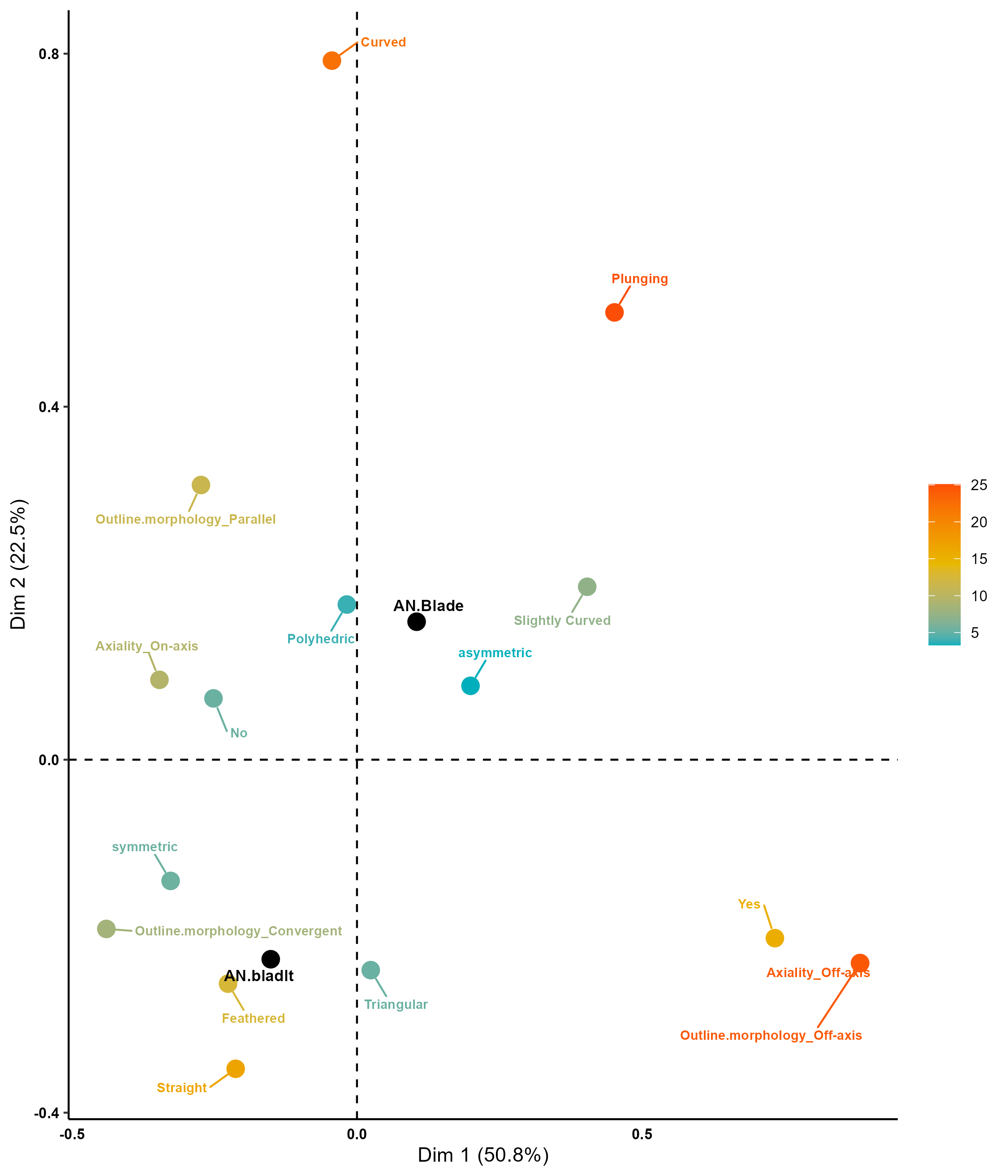

Supplement: S1 Fig — (ZIP) [file pone.0331393.s004.zip › Supporting_Information_Figures/SI_Figures_MCA-Biplots_Correlation-Plots/SIFig52_MCAConvexityAN.tiff]

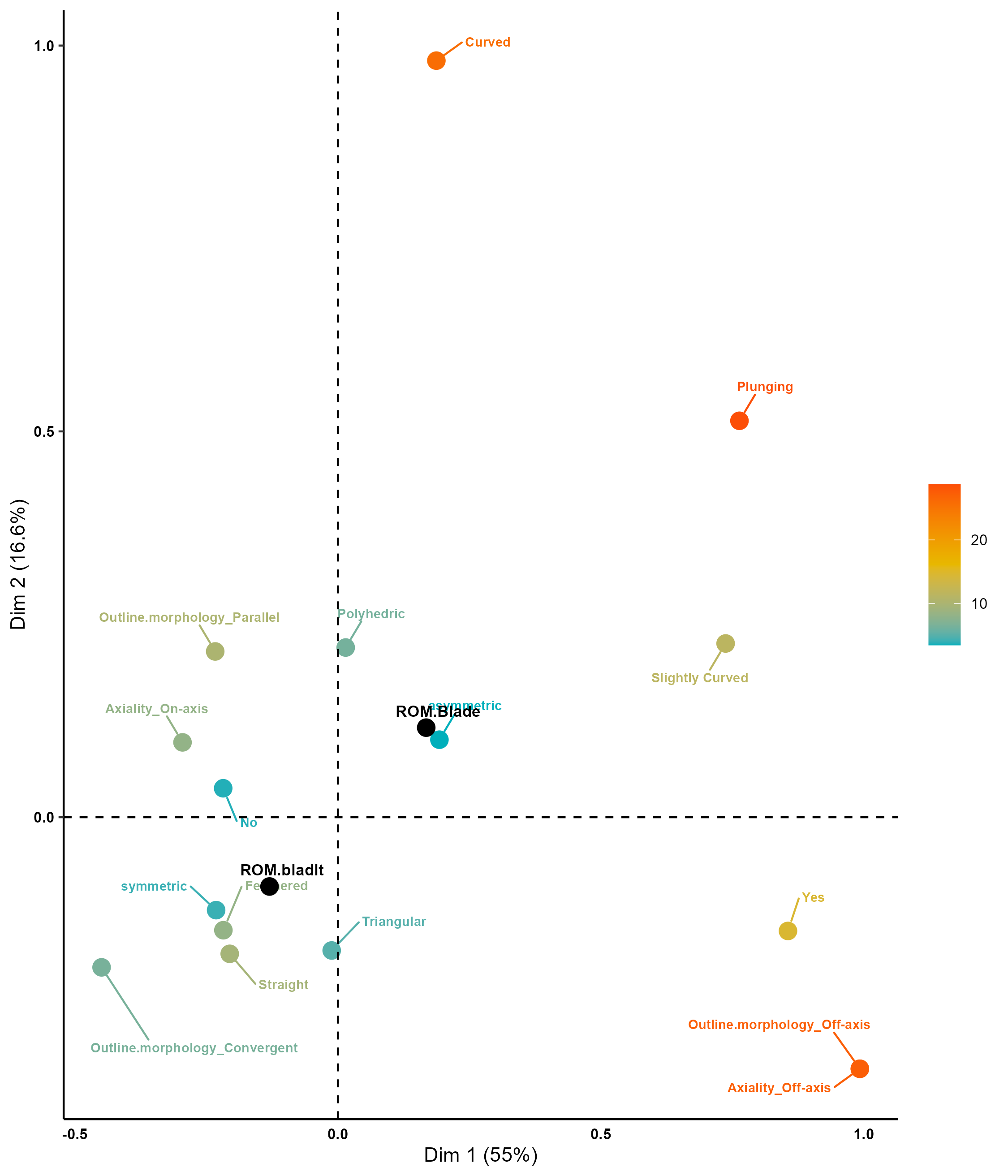

Supplement: S1 Fig — (ZIP) [file pone.0331393.s004.zip › Supporting_Information_Figures/SI_Figures_MCA-Biplots_Correlation-Plots/SIFig53_MCAConvexityROM.tiff]

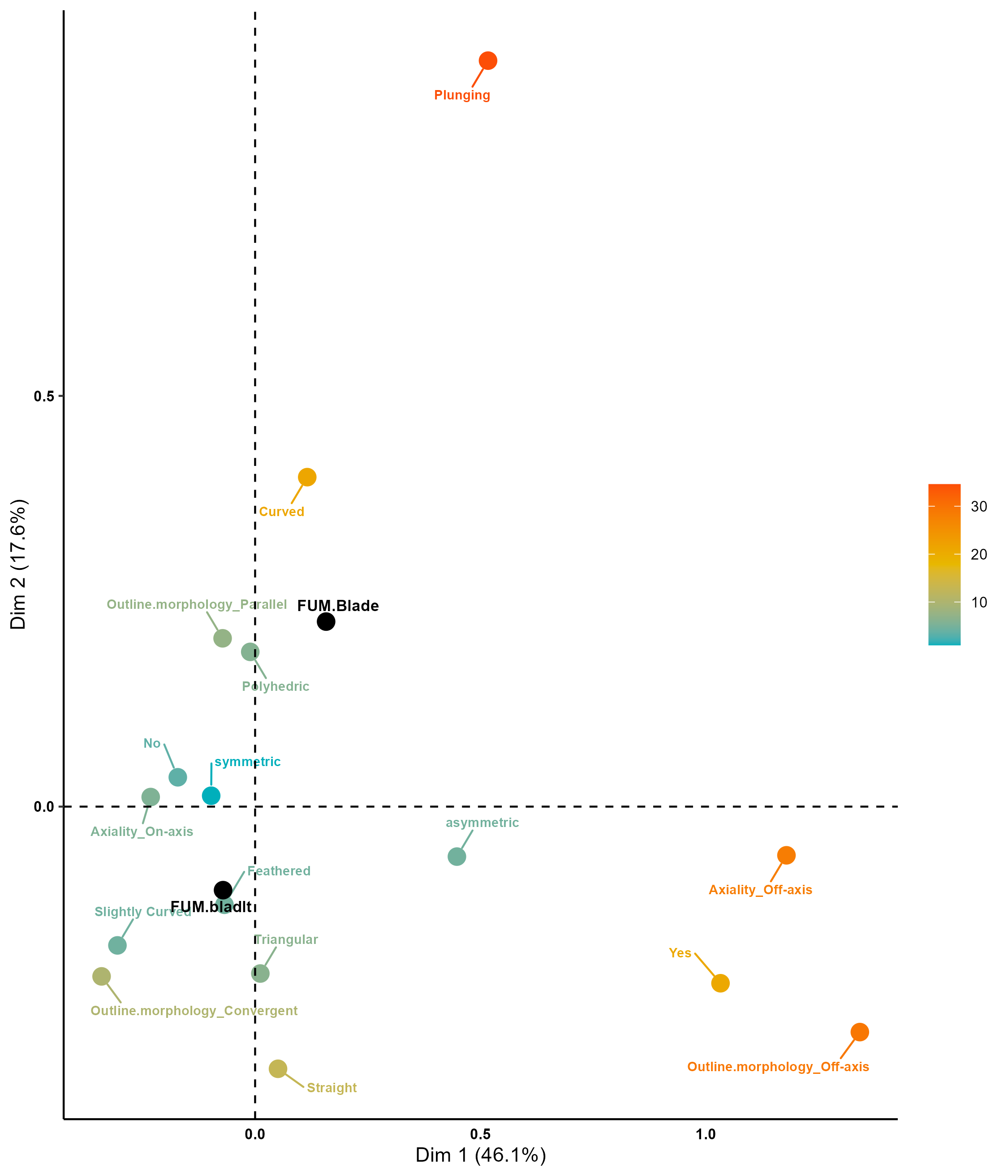

Supplement: S1 Fig — (ZIP) [file pone.0331393.s004.zip › Supporting_Information_Figures/SI_Figures_MCA-Biplots_Correlation-Plots/SIFig54_MCAConvexityFUM.tiff]

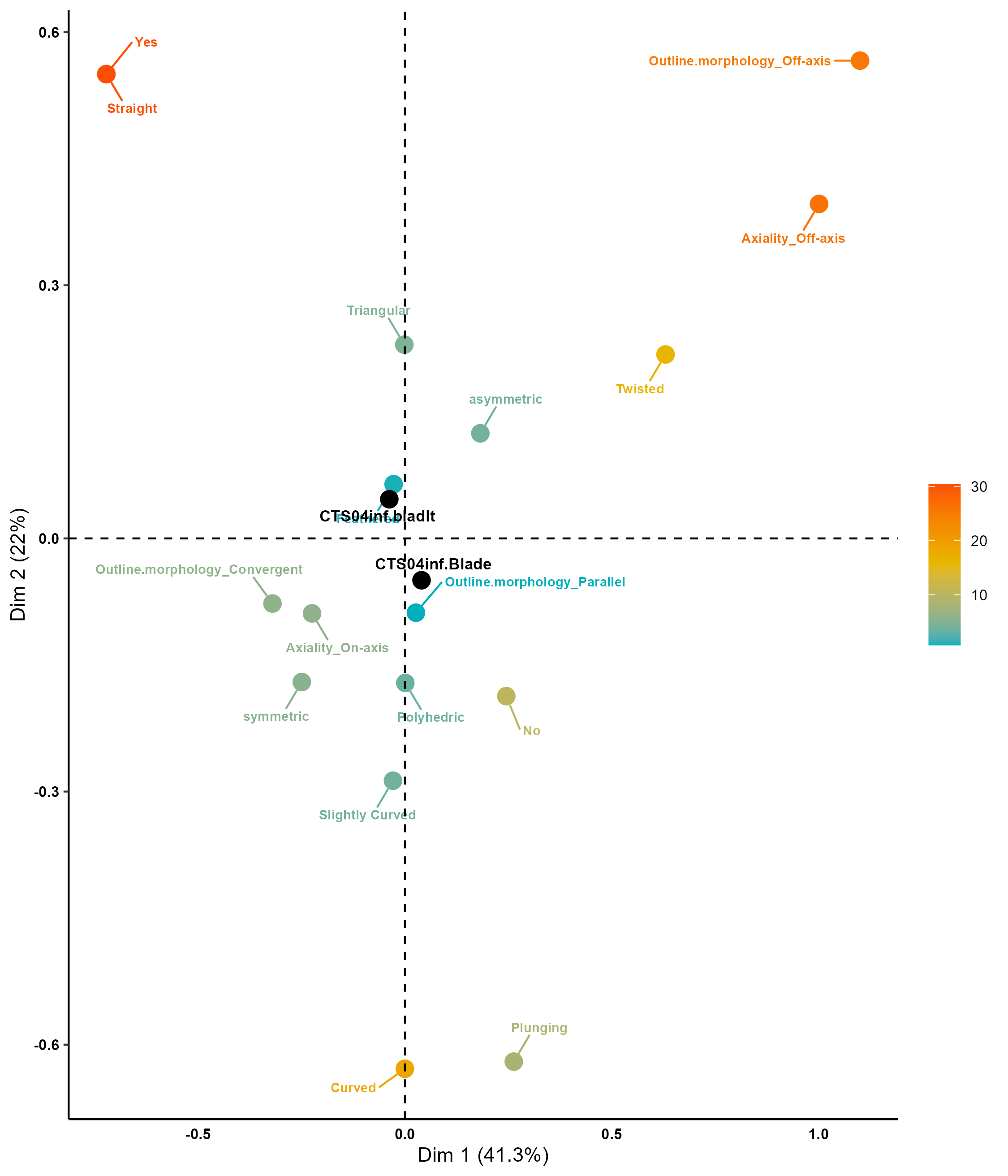

Supplement: S1 Fig — (ZIP) [file pone.0331393.s004.zip › Supporting_Information_Figures/SI_Figures_MCA-Biplots_Correlation-Plots/SIFig55_MCAConvexityCTS.tiff]

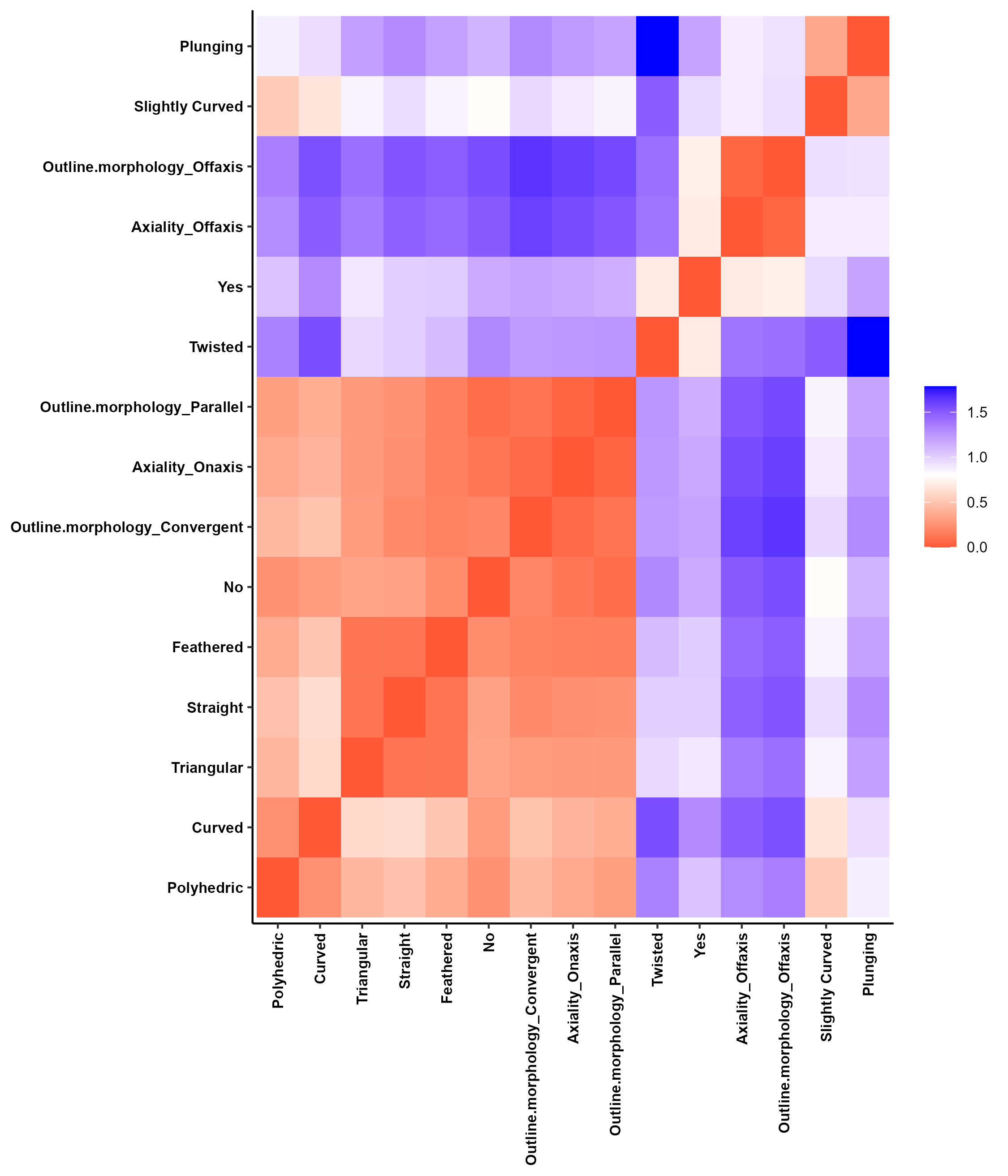

Supplement: S1 Fig — (ZIP) [file pone.0331393.s004.zip › Supporting_Information_Figures/SI_Figures_MCA-Biplots_Correlation-Plots/SIFig56_CORRConvexityCONTROL.tiff]

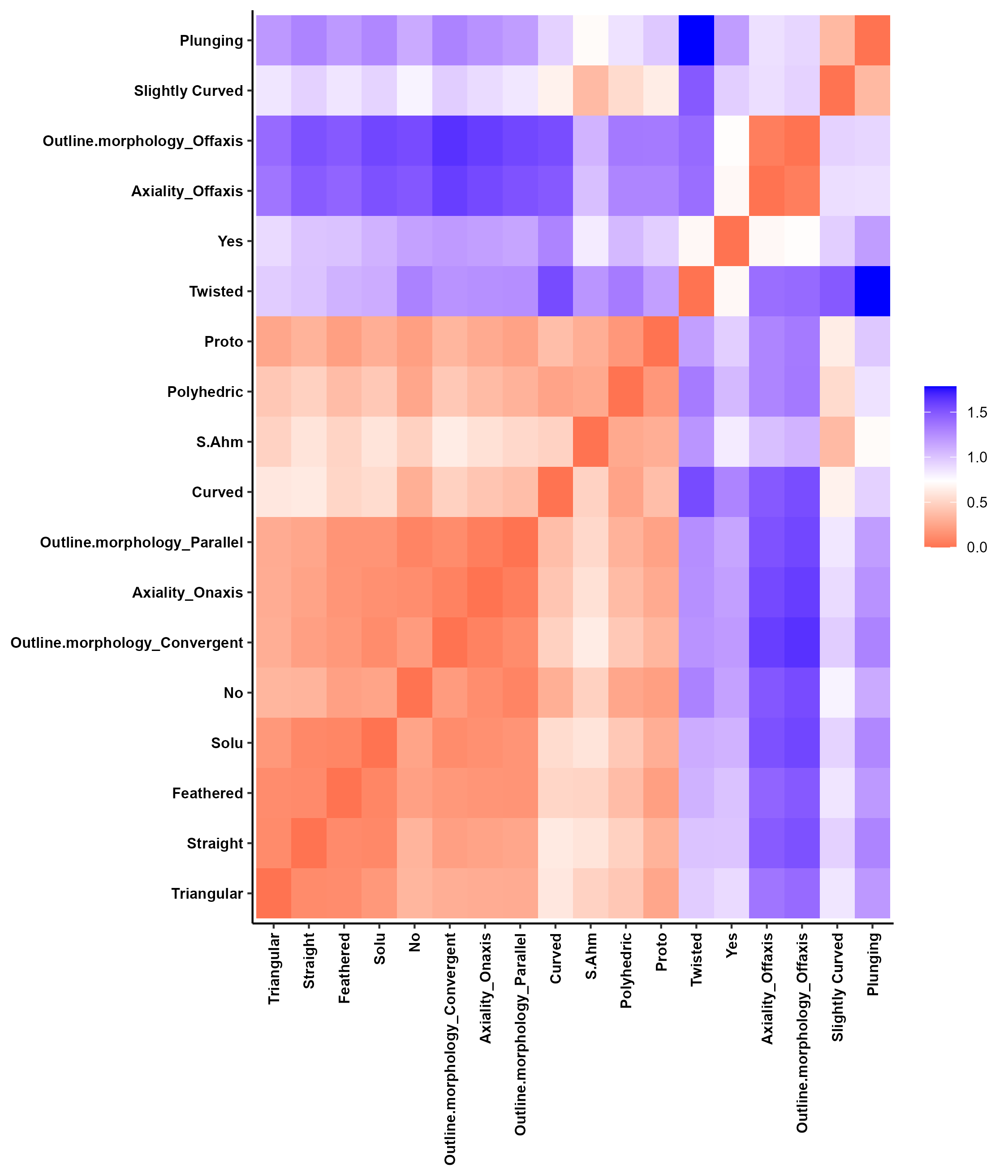

Supplement: S1 Fig — (ZIP) [file pone.0331393.s004.zip › Supporting_Information_Figures/SI_Figures_MCA-Biplots_Correlation-Plots/SIFig57_CORRConvexityCONTROL_withquali.tiff]

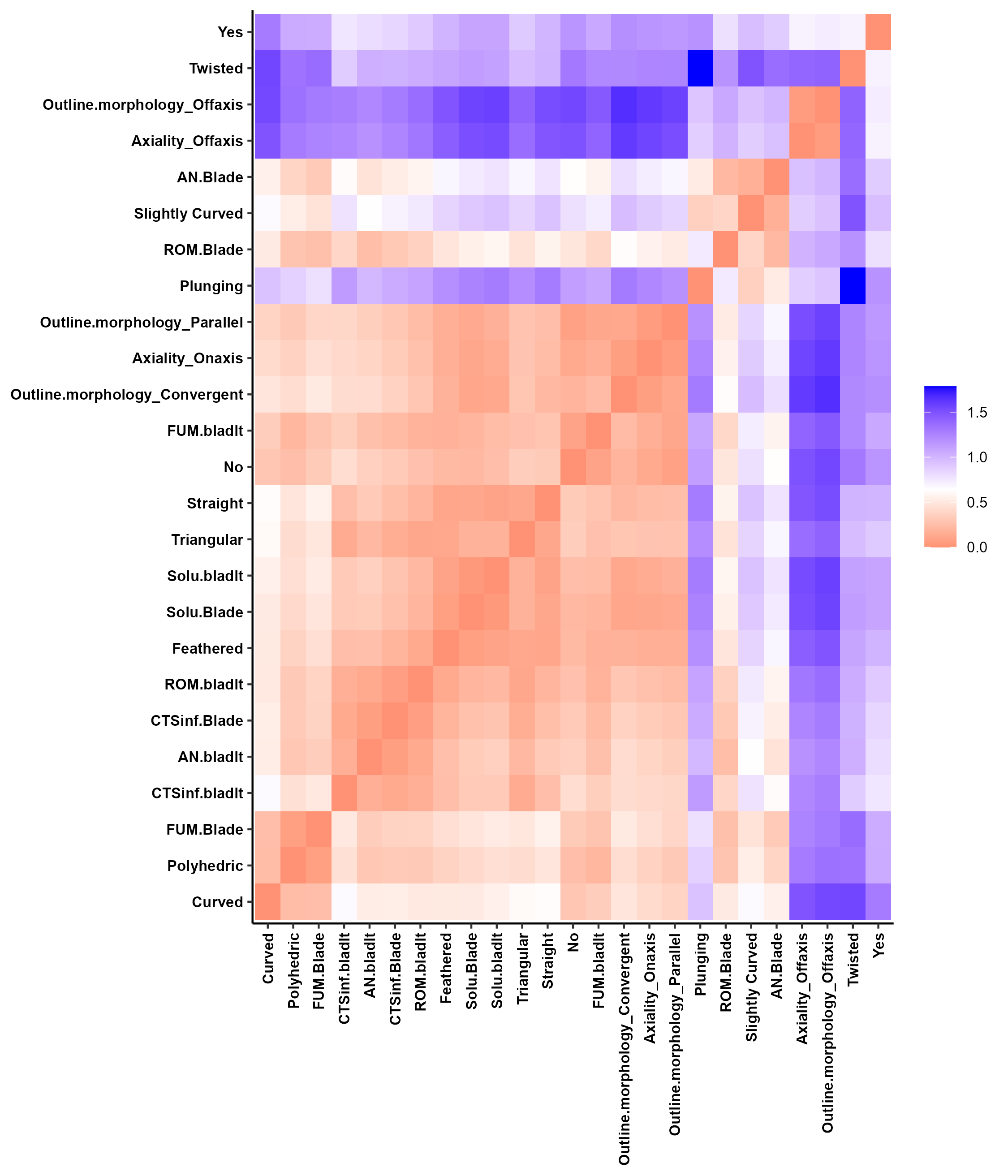

Supplement: S1 Fig — (ZIP) [file pone.0331393.s004.zip › Supporting_Information_Figures/SI_Figures_MCA-Biplots_Correlation-Plots/SIFig58_CORRConvexityCONTROL_withquali_2.tiff]

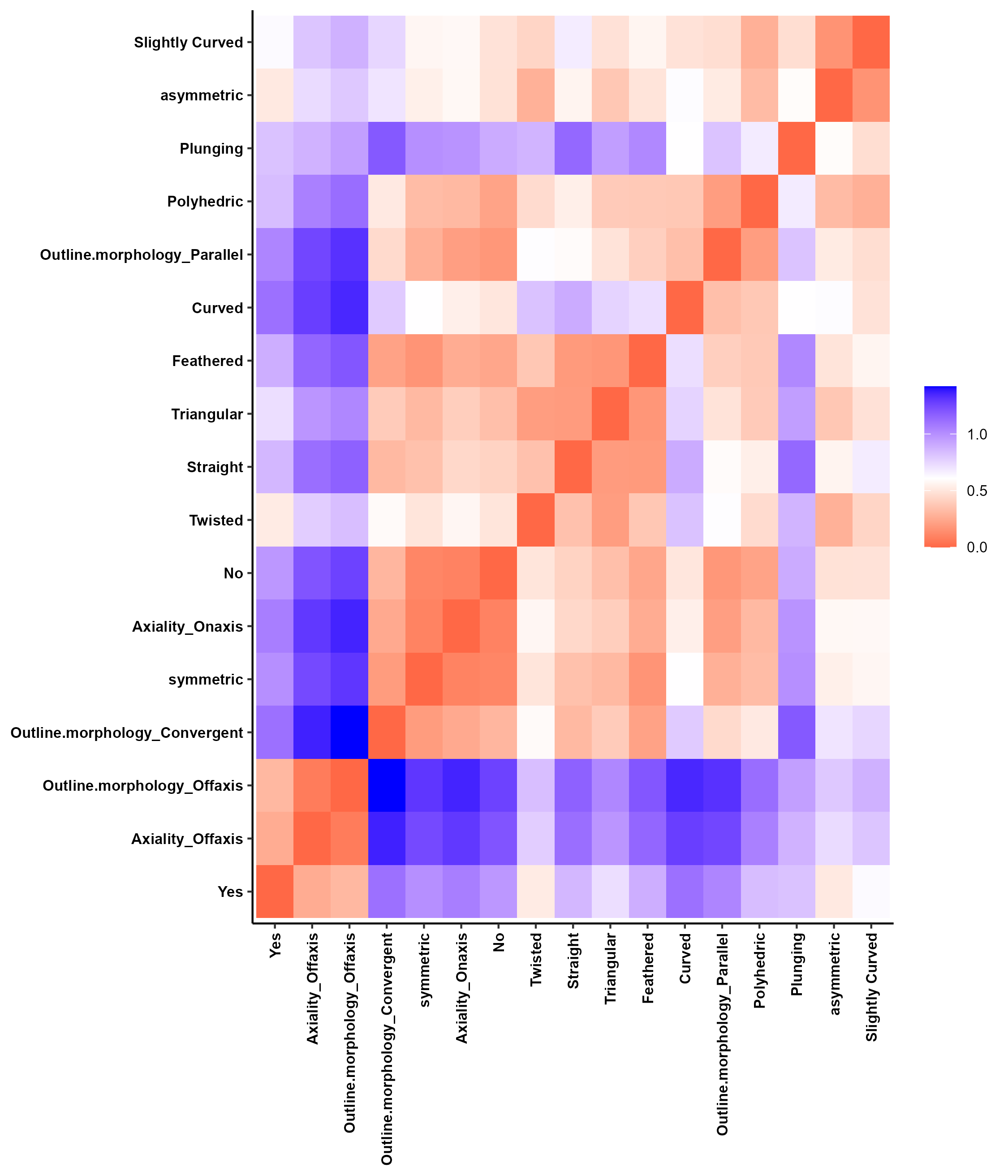

Supplement: S1 Fig — (ZIP) [file pone.0331393.s004.zip › Supporting_Information_Figures/SI_Figures_MCA-Biplots_Correlation-Plots/SIFig59_CORRConvexityALL.tiff]

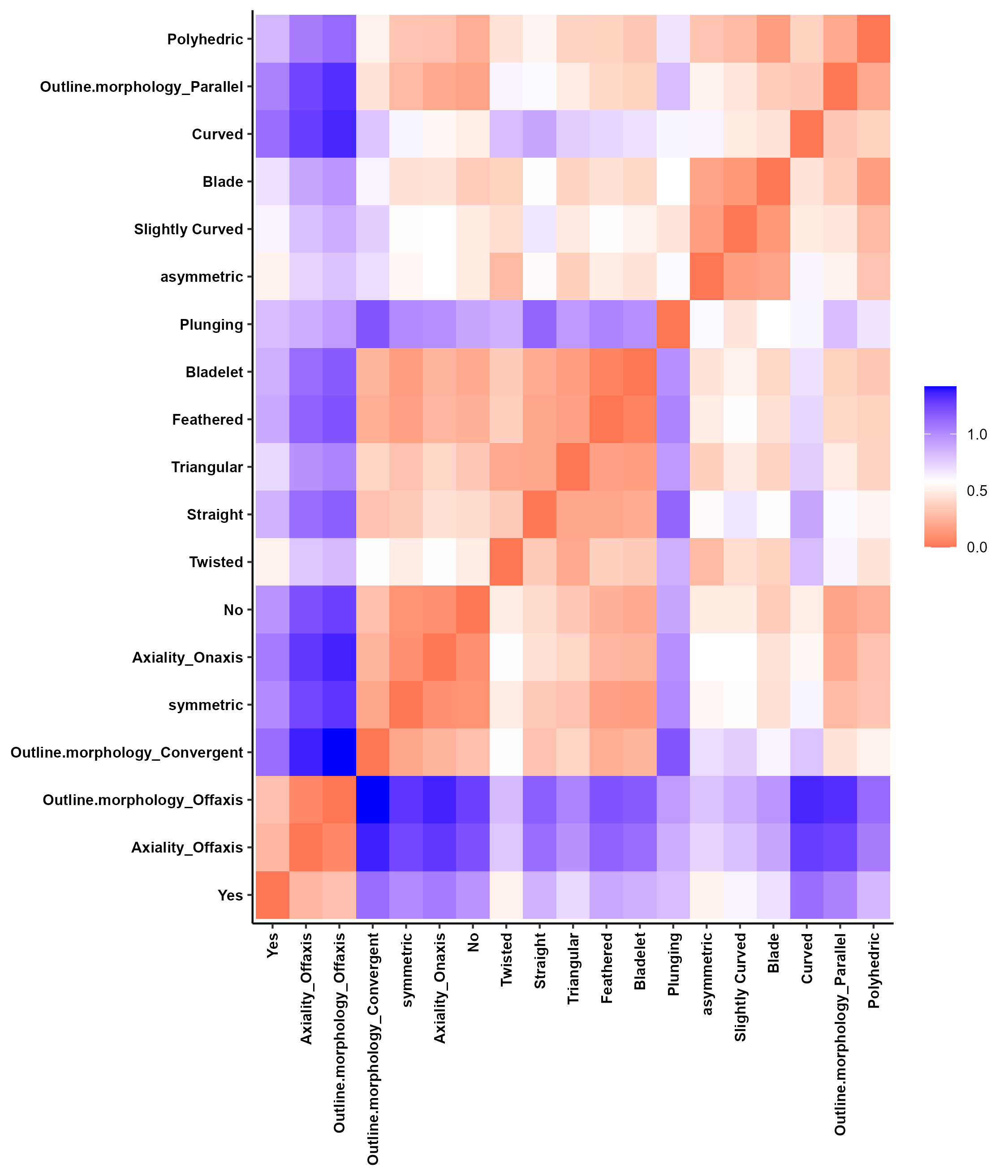

Supplement: S1 Fig — (ZIP) [file pone.0331393.s004.zip › Supporting_Information_Figures/SI_Figures_MCA-Biplots_Correlation-Plots/SIFig60_CORRConvexityALL_withquali.tiff]

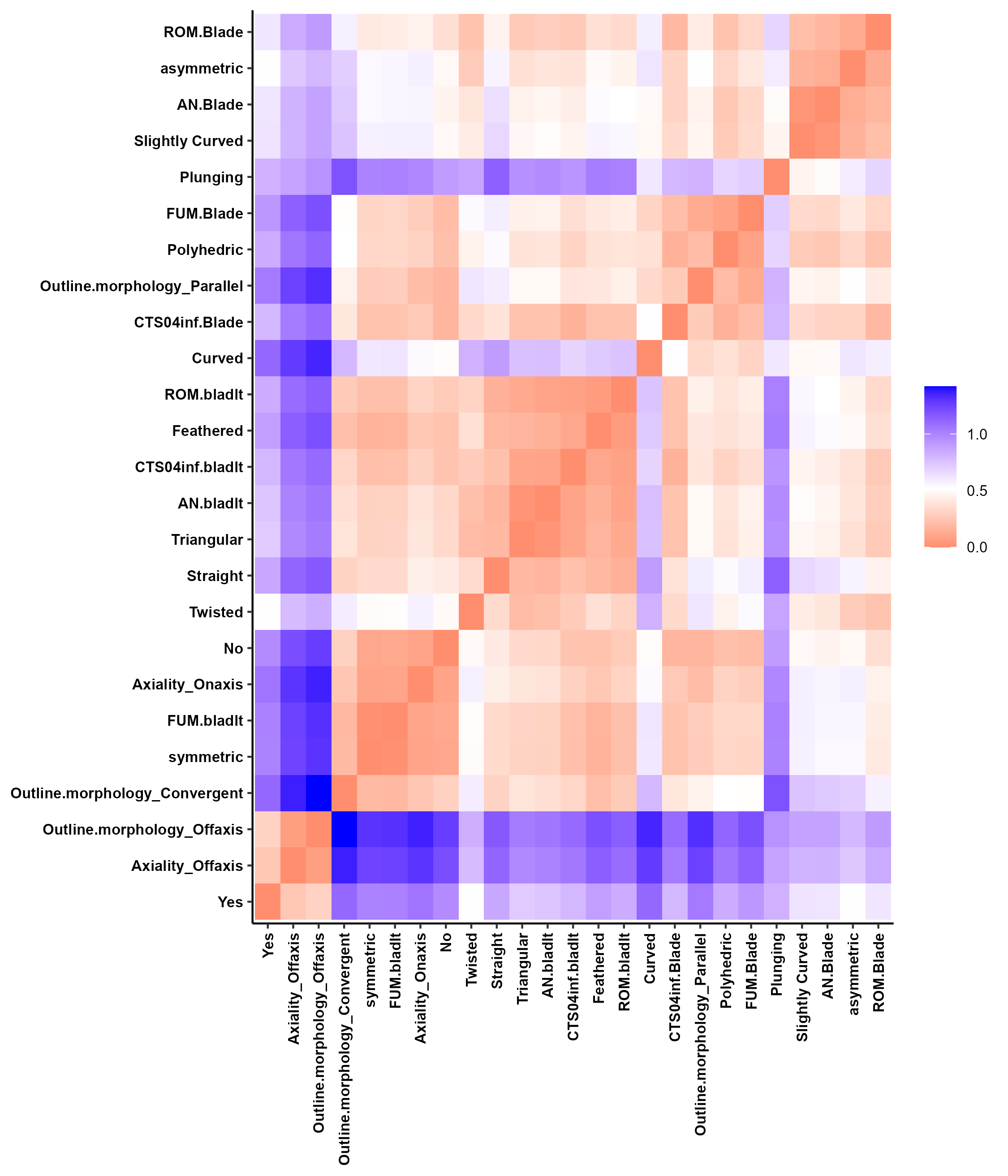

Supplement: S1 Fig — (ZIP) [file pone.0331393.s004.zip › Supporting_Information_Figures/SI_Figures_MCA-Biplots_Correlation-Plots/SIFig61_CORRConvexityALL_withquali_2.tiff]

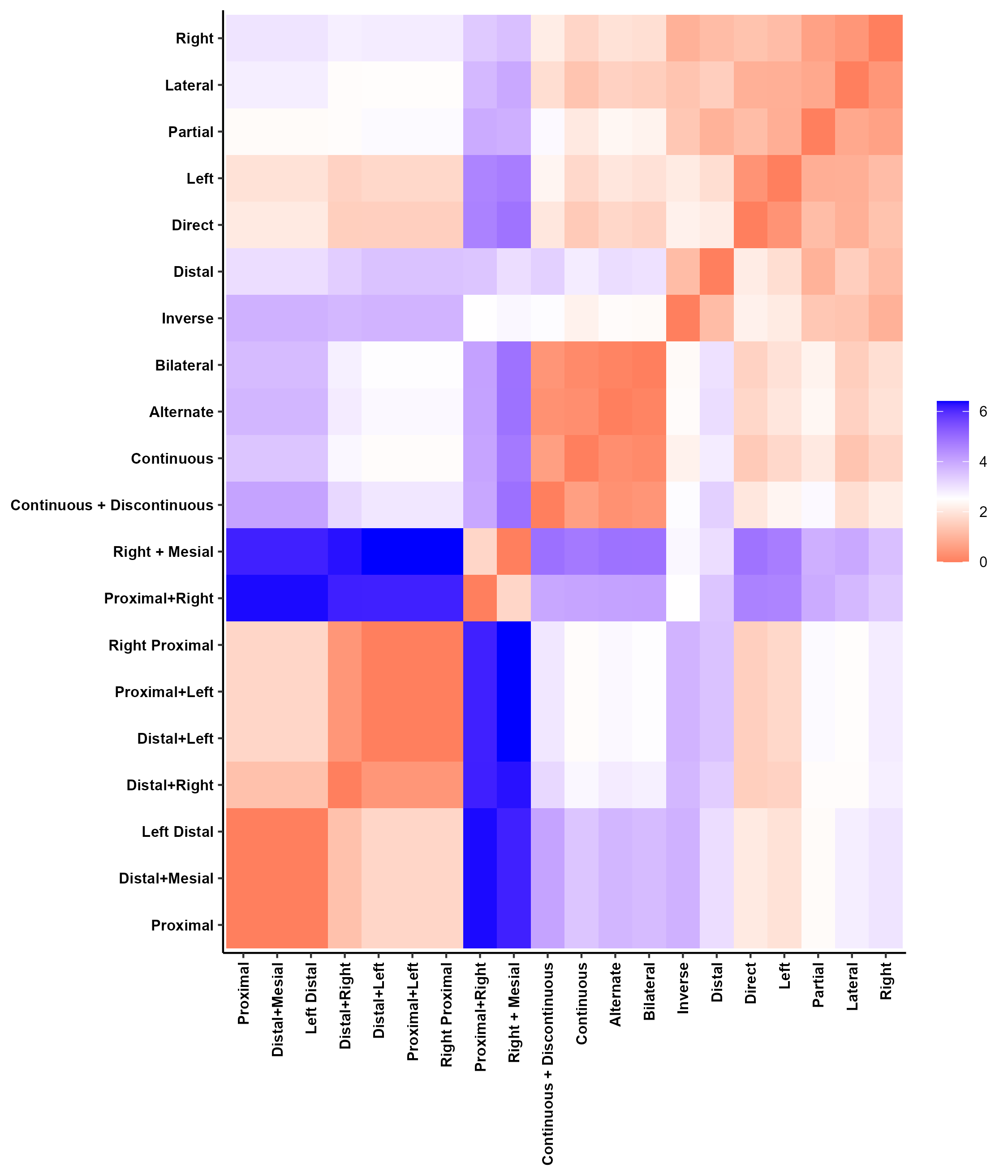

Supplement: S1 Fig — (ZIP) [file pone.0331393.s004.zip › Supporting_Information_Figures/SI_Figures_MCA-Biplots_Correlation-Plots/SIFig62_CORRretouchingALL.tiff]

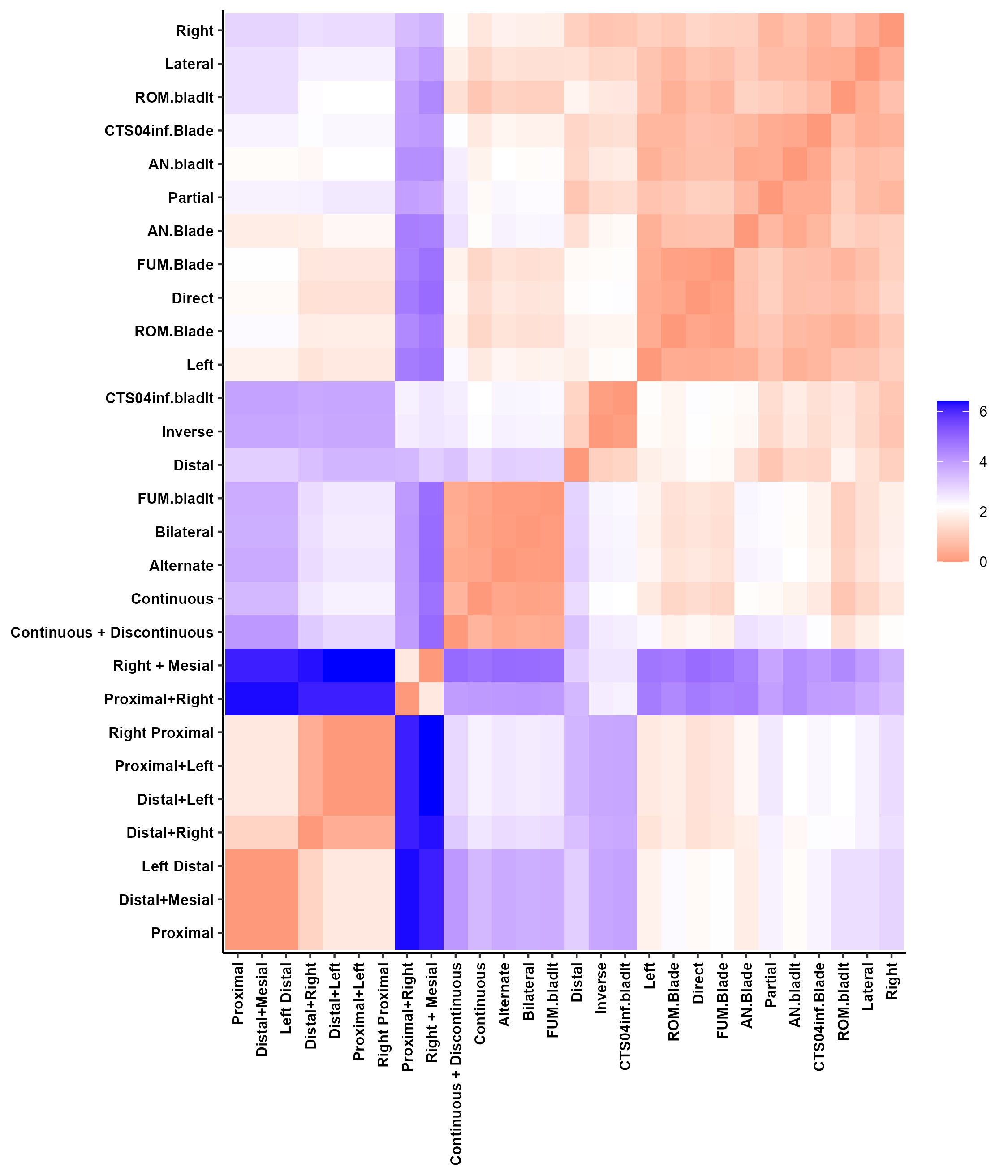

Supplement: S1 Fig — (ZIP) [file pone.0331393.s004.zip › Supporting_Information_Figures/SI_Figures_MCA-Biplots_Correlation-Plots/SIFig63_CORRretouchingALL_withquali.tiff]
